# GLP-1 and glucagon receptor dual agonism ameliorates kidney allograft fibrosis by improving lipid metabolism

**DOI:** 10.3389/fimmu.2025.1551136

**Published:** 2025-03-31

**Authors:** Linjie Peng, Weijie Lai, Shuangjin Yu, Qihao Li, Xianxin Jiang, Guodong Chen

**Affiliations:** ^1^ Organ Transplant Center, The First Affiliated Hospital of Sun Yat-sen University, Guangzhou, China; ^2^ Kidney Transplantation Department II, Shenzhen Third People’s Hospital, Shenzhen, China; ^3^ Guangdong Provincial Key Laboratory of Organ Medicine, Guangzhou, China; ^4^ Guangdong Provincial International Cooperation Base of Science and Technology (Organ Transplantation), Guangzhou, China; ^5^ School of Pharmaceutical Sciences, Sun Yat-sen University, Guangzhou, China

**Keywords:** GLP-1r, GCGR, TB001, allograft fibrosis, lipid metabolism

## Abstract

**Introduction:**

Kidney allograft fibrosis accelerates the progression of chronic kidney disease (CKD), leads to allograft failure, and increases patient mortality. Emerging evidence suggests that metabolic syndrome in transplant recipients is associated with fibrosis development. However, it remains unclear whether targeting metabolic pathways can mitigate allograft fibrosis. This study aimed to explore the potential of targeting metabolic pathways using the GLP-1R/GCGR dual agonist TB001 for the treatment of kidney allograft fibrosis.

**Methods:**

Kidney allograft fibrosis was induced in rat kidney transplant models. Histological analysis, transcriptome sequencing, and *in vitro* experiments were performed to investigate the efficacy of TB001 and its underlying mechanisms.

**Results:**

Compared with the control group, TB001-treated recipients had significantly improved kidney allograft function, as evidenced by lower creatinine and 24-hour urine protein levels. Moreover, TB001 treatment decreased the body weight and serum total cholesterol, LDL-cholesterol, and TNF-α levels in transplant recipients, indicating metabolic improvements. Pathological analysis demonstrated that TB001 treatment reduced inflammatory cell infiltration and downregulated the expression of fibrosis markers, including TGF-β1, α-SMA, COL1A1, and Vimentin. Further transcriptome sequencing of kidney grafts revealed that TB001-treated group had a gene expression pattern similar to that of the syngeneic control group and showed significant enhancement of lipid metabolism-related pathways, particularly the PPAR pathway. *In vivo* and *in vitro* experiments further demonstrated that TB001 upregulated the expression of CPT1A, a key molecule involved in lipid metabolism, and inhibited TGF-β1/Smad2/3/Twist and PKC-α/PKC-β pathways.

**Conclusion:**

Targeting metabolic pathways using the GLP-1R/GCGR dual agonist TB001 shows potential for managing kidney allograft fibrosis.

## 1 Introduction

Kidney transplantation, the preferred treatment for stage 4 chronic kidney disease (CKD), achieves 1-year, 5-year, and 10-year survival rates of 91%, 77%, and 56%, respectively (1). Although short-term survival rates of transplant recipients have significantly improved, long-term survival remains challenging. Kidney allograft failure accounts for 12% of the transplant waiting list, with repeat transplantations comprising 10.4% of all kidney transplants (2). Chronic kidney allograft dysfunction (CAD) is considered a major impediment to long-term survival, characterized by gradual deterioration of graft function that occurs months to years post-transplant (3). The primary pathological features of CAD include interstitial fibrosis and tubular atrophy (IF/TA), or inflammation with interstitial fibrosis and tubular atrophy (i-IFTA), both of which currently lack effective therapeutic interventions (4).

The pathogenesis of kidney allograft fibrosis is complex, involving both immune-mediated and non-immune factors (5). Among these factors, epithelial-mesenchymal transition (EMT) has been recognized as a critical mechanism driving kidney allograft fibrosis (6, 7). Notably, metabolic syndrome (MS), characterized by hypertension, abnormal blood glucose or diabetes, dyslipidemia, and abdominal obesity, affects 20% to 65% of kidney transplant recipients (8, 9). MS is strongly associated with the onset and progression of chronic kidney disease (CKD) (10, 11). In kidney transplant recipients, MS not only increases the risk of postoperative infections and mortality but also compromises graft survival and the quality of life (12, 13). Additionally, hyperlipidemia has been proved that facilitating chronic interstitial fibrosis in transplanted kidneys (14). Furthermore, MS is prevalent among expanded criteria donors (ECDs) and is strongly associated with delayed graft function (DGF) (15–18). Consequently, therapeutic approaches targeting MS are hypothesized to help mitigate kidney allograft fibrosis.

Insulin resistance is the central pathophysiological feature of MS (19). Activation of the glucagon-like peptide-1 receptor (GLP-1R) has been shown to reduce body weight and food intake while enhancing glucose control. Concurrent activation of the glucagon receptor (GCGR) can reduce hepatic lipid content, enhance glycogen flux, and improve mitochondrial function (20, 21). Whether activation of GLP-1R and GCGR can ameliorate transplanted kidney fibrosis calls for experimental explorations. Recently, the GLP-1R agonist liraglutide has been shown to alleviate kidney fibrosis in models of unilateral ureteral obstruction (UUO) and angiotensin II-induced kidney fibrosis (22–24). Additionally, accumulating evidence suggests that GLP-1 exerts protective effects on kidney function among CKD patients (25, 26). However, up to date, the role of GLP-1R or GCGR in the study of transplanted kidney fibrosis could not be found in literatures.

TB001, a dual agonist of GLP-1R and GCGR, synthesized by School of Pharmaceutical Sciences of Sun Yat-sen University, effectively regulates glucose levels and body weight. Previous work by our laboratory has demonstrated the significant anti-fibrotic effects of TB001 in models of liver fibrosis (27). And our previous work has revealed a critical correlation between dysregulated lipid metabolism and kidney allograft fibrosis (7). Based on these findings, TB001 is hypothesized to be effective in mitigating kidney allograft fibrosis, and supposed to work associated with lipid metabolism related pathway.

## 2 Materials and methods

### 2.1 Experimental animals and ethical statements

Outbred rats (Sprague-Dawley [SD] and Wistar) and inbred rats (F344 and Lewis) were obtained from Beijing Vital River Laboratory Animal Technology Co., Ltd. (Beijing, China). All experimental procedures were conducted in strict accordance with the guidelines of the Laboratory Animal Management and Use Committee of Sun Yat-sen University and were approved by the Laboratory Animal Ethics Committee of Sun Yat-sen University (approval numbers: SYSU-IACUC-2022-000133 and SYSU-IACUC-2023-000443).

### 2.2 Kidney transplantation in rats

Following previously established protocols (7, 28), rat kidney transplantation was performed via *in situ* end-to-end anastomosis on the left side of recipients. To facilitate transplanted kidney fibrosis, the harvested kidney was storage in 4°C Heparin saline before transplantation. Tacrolimus (0.1 mg/kg/day) was administered intraperitoneally for the first 7 postoperative days to prevent acute rejection. Right kidney nephrectomy of recipient rats was performed on the 5th post-transplant day. The experimental animals were randomly assigned to the vehicle group and TB001 group after transplantation. The TB001 group (SD to Wistar, or F344 to Lewis) received intraperitoneal injections of TB001 (56 μg/kg/day, Shenzhen Turier Biotech Co., Ltd., Shenzhen, China). The vehicle group (SD to Wistar, or F344 to Lewis) received the same volume of saline and served as the allogenic control. The syngeneic control (Wistar to Wistar, or Lewis to Lewis) was also included as a normal control. The inbred Lewis-F344 rat kidney transplant models were used for a 14-weeks observation and subsequent mechanistic investigations, while the outbred SD-Wistar rat kidney transplant models were used for a 26-weeks survival analysis.

### 2.3 Renal function and urinary protein measurements

Blood samples were collected from the tail vein and analyzed using an Abbott i-STAT 1 portable clinical analyzer (Abbott Laboratories, Chicago, USA). Creatinine and blood urea nitrogen (BUN) levels were measured weekly post-transpant from week 1 through week 14. 24-hour urine samples were collected on days 30th, 60th, and 90th post-transplant day. A urinary protein quantitation kit was obtained from Nanjing Jiancheng Bioengineering Institute (Nanjing, China). Urinary protein quantitation was performed following the manufacturer’s instructions.

### 2.4 Serum lipid assay

Test kits for triglycerides, total cholesterol, low-density lipoprotein (LDL) cholesterol, and high-density lipoprotein (HDL) cholesterol were purchased from Nanjing Jiancheng Bioengineering Institute (Nanjing, China). All assays were performed in strict accordance with the manufacturer’s protocol.

### 2.5 Enzyme-linked immunosorbent assay

Enzyme-linked immunosorbent assay (ELISA) kits for rat TNF-α, TGF-β, IL-1β, IL-4, and IL-10 were obtained from Meimian Science Inc. (Jiangsu, China). The levels of these inflammatory factors were quantified according to the manufacturer’s instructions.

### 2.6 Transcriptome sequencing

Eukaryotic transcriptome sequencing of kidney grafts was conducted by Sangon Biotechnology (Shanghai, China). All kidney samples from the F344-Lewis kidney transplantation models were qualified for sequencing analysis, comprising 6 samples from the syngeneic group, 7 samples from vehicle-treated group, and 8 samples from TB001-treated group. Expression level analysis, differential expression analysis (log2FoldChange > 1 and P-value < 0.05), and Kyoto Encyclopedia of Genes and Genomes (KEGG) pathway enrichment analysis were performed.

### 2.7 Quantitative real-time polymerase chain reaction

All qPCR reagents were obtained from Accurate Biotechnology (Hunan, China). Briefly, total RNA was extracted from kidney tissue using the SteadyPure Universal RNA Extraction Kit. Complementary DNA (cDNA) was synthesized using the Evo M-MLV RT Premix for qPCR. Quantitative PCR was performed using the SYBR^®^ Green Premix Pro Taq HS qPCR Kit II (Rox Plus) on a BIORAD qPCR system. The expression of each target gene was normalized to β-actin using the 2^-ΔΔCt^ method. The primers for target genes are listed in **Supplementary Table 1**.

### 2.8 Immunohistochemistry assay

Paraffin-embedded transplanted kidney tissues were sectioned at 5 μm thickness. Following deparaffinization, rehydration, and antigen retrieval, endogenous peroxidase activity was quenched with 3% hydrogen peroxide, and non-specific binding was blocked with 10% goat serum. Sections were incubated with primary antibodies at 4°C overnight. HRP-conjugated secondary antibodies were applied and incubated at room temperature for 45 minutes. Color development was achieved using 3,3’-diaminobenzidine (DAB) staining, and images were captured using the OLYMPUS microscope (Tokyo, Japan). The information of antibody used in the IHC assay was listed in the **Supplementary Table 1**.

### 2.9 Cell culture

HK-2 cells (Cat. No.: TCH-C400) was purchased from Haixing Biosciences (Hangzhou, China). DMEM supplemented with 10% fetal bovine serum (FBS) and 1% penicillin-streptomycin was used for culture. TGF-β1 (ABclonal, 10ng/mL), protein kinase C (PKC) agonist phorbol 12-myristate 13-acetate (PMA, MedChemExpress, 11.7nM), PKC inhibitor GO6983 (MedChemExpress, 7nM) and the MEK inhibitor PD98059 (MedChemExpress, 10μM), and TB001 (10μM) were used to testify.

For the scratch assay, confluent HK-2 cell monolayers were serum-starved for 24 hours. A vertical scratch was made in the center of each well using a sterile 200 μL pipette tip. After washing twice with PBS, cells were incubated with intervention-containing media for 24 hours. Wound closure was measured at 0 and 24 hours post-scratch. For the transwell migration assay, HK-2 cells in the logarithmic growth phase were trypsinized and washed three times with serum-free medium to ensure complete removal of FBS. Cells were then resuspended in serum-free medium containing the respective interventions at a concentration of 1-10 × 10^5^ cells/mL. 700 μL of complete medium was firstly added to the lower chamber, and 200 μL of cell suspension was then carefully added to the upper chamber. After 24 hours, cell migration was quantified.

### 2.10 Immunofluorescence assay

Cells were fixed with 4% paraformaldehyde for 15 minutes, permeabilized with 0.5% Triton X-100 for 20 minutes, and blocked with 10% goat serum for 30 minutes. Cells were incubated with primary antibodies (rabbit anti-rat IgG) overnight at 4°C. Cells were washed three times with TBST. Goat anti-rabbit IgG fluorescent secondary antibodies were applied and incubated at room temperature for 1 hour in the dark. After washing, slides were mounted with antifade solution containing DAPI. Images were immediately captured using fluorescence microscope (OLYMPUS, Tokyo, Japan). The information of antibody used in the IF assay was listed in the **Supplementary Table 1**.

### 2.11 Western blot assay

Cellular proteins were extracted using RIPA Buffer supplemented with protease inhibitor cocktail (both from Beyotime, Shanghai, China). Protein concentrations were determined using the BCA assay, and samples were adjusted to equal concentrations before denaturation. Proteins were separated by SDS-PAGE and transferred to 2.5 μm PVDF membranes (Millipore, Burlington, MA, USA). Membranes were blocked with 2.5% skim milk for 75 minutes at room temperature with gentle shaking. Membranes were incubated with primary antibodies overnight at 4°C, followed by HRP-conjugated secondary antibodies for 75 minutes at room temperature. Protein bands were visualized using SuperKine™ West Femto Maximum Sensitivity Substrate (Abbkine, Wuhan, China) for chemiluminescence detection. The information of antibody used in the WB assay was listed in the **Supplementary Table 1**.

### 2.12 Statistical analysis

Semi-quantitative image analysis was performed using ImageJ software (NIH, Bethesda, MD, USA). Statistical analyses were performed using GraphPad Prism 9.5 (GraphPad Software, San Diego, CA, USA). Results are presented as mean ± standard deviation. In the present study, comparisons were all between multiple groups, One-way ANOVA followed by Tukey’s *post-hoc* test was used when equal variance was confirmed, otherwise Kruskal-Wallis would be used. A p-value < 0.05 was considered statistically significant.

## 3 Result

### 3.1 GLP-1R/GCGR dual agonist TB001 treatment significantly improved kidney allograft function

**Figure 1:**
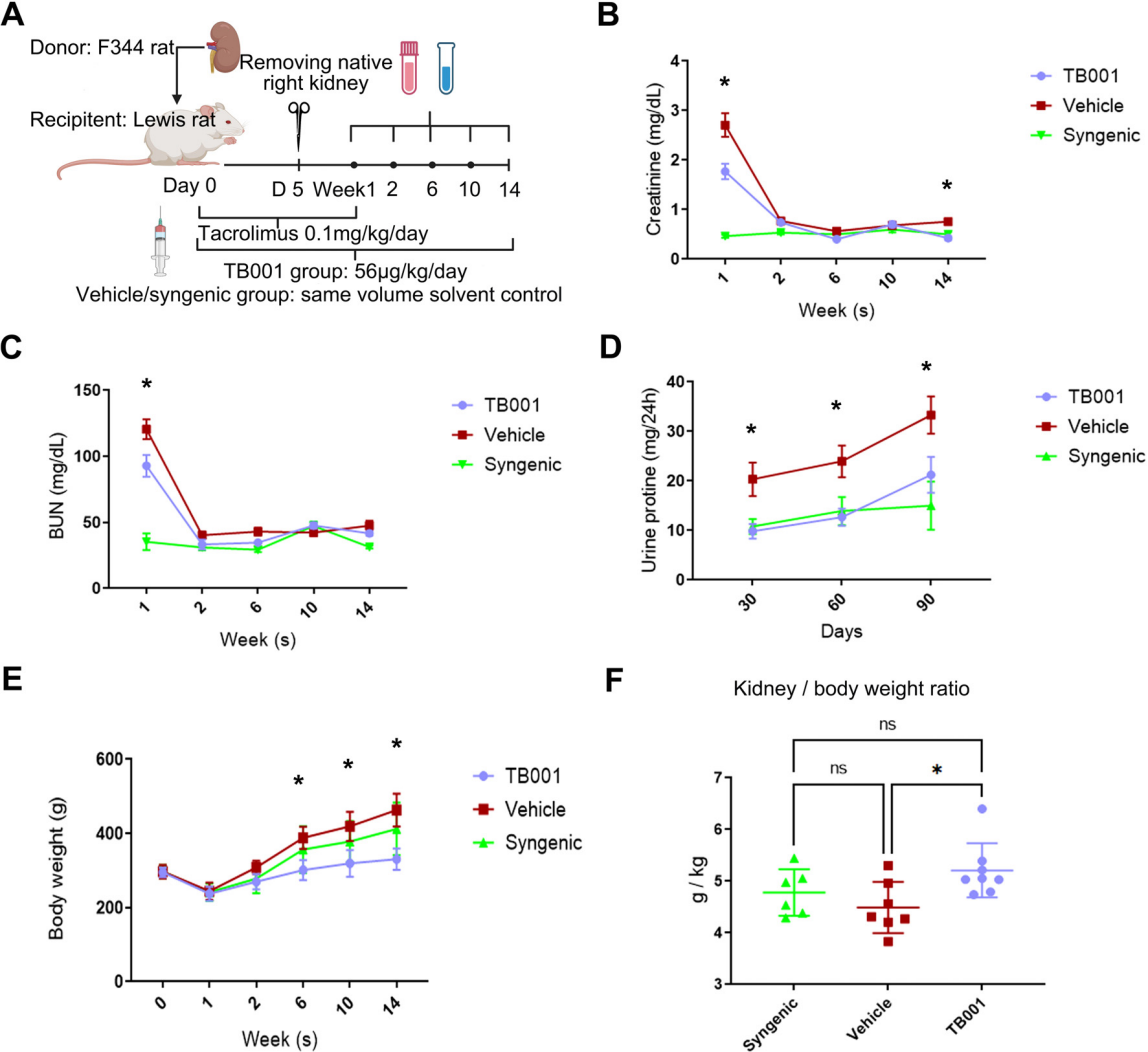
TB001 improved outcomes of rat kidney transplantation. **(A)** The study protocol of TB001 in rat kidney transplantation using F344 and Lewis rats. Levels of blood creatinine **(B)**, blood urea nitrogen (BUN) **(C)**, 24-hour urinary protein **(D)**, and body weight **(E)** of transplant recipients at different time points of the three groups were shown. **(F)** bar graph shows transplanted kidney-to-recipient weight ratio at the study endpoint. ^*^ Difference shown between the vehicle group VS. the TB001 group, One-way ANOVA followed by Tukey’s *post-hoc* test.

The primary study protocol of TB001 treatment in rat kidney transplantation models is illustrated in **Figure 1A**. Compared with the vehicle treated group, TB001-treated recipients demonstrated significantly lower levels of creatinine at the first week (*P* < 0.0001, **Figure 1B**) and the 14^th^ week (*P* = 0.0001, **Figure 1B**) post-transplantation, as well as a significantly lowere level of BUN at the first week (P <0.0001, **Figure 1C**), indicating improved graft function. Moreover, TB001 treatment significantly decreased urinary protein excretion at days 30, 60, and 90 post-transplant compared with the vehicle treated group (*P* < 0.05, **Figure 1D**). TB001 treatment also reduced the body weight of recipients (*P* = 0.0002, **Figure 1E**) without affecting the weight of the transplanted kidneys, as evidenced by a higher graft-to-body weight ratio in the TB001 group (*P* = 0.037, **Figure 1F**). No mortality was observed during the 14-week study period, demonstrating the safety of TB001 with current therapeutic dose. We then extended the observation time to 26 weeks in SD-Wistar rat kidney transplantation models. Similarly, TB001 significantly reduced the recipients’ body weight (*P* = 0.006, **Supplementary Figure 1A**) during the 26-weeks observation. TB001-treated group exhibited lower mortality compared to the control group, although the difference was not statistically significant (3/10 vs. 1/7, *P* = 0.31, **Supplementary Figure 1B**).

### 3.2 TB001 exerted anti-inflammatory and cholesterol-lowering effects in kidney transplant recipients

**Figure 2:**
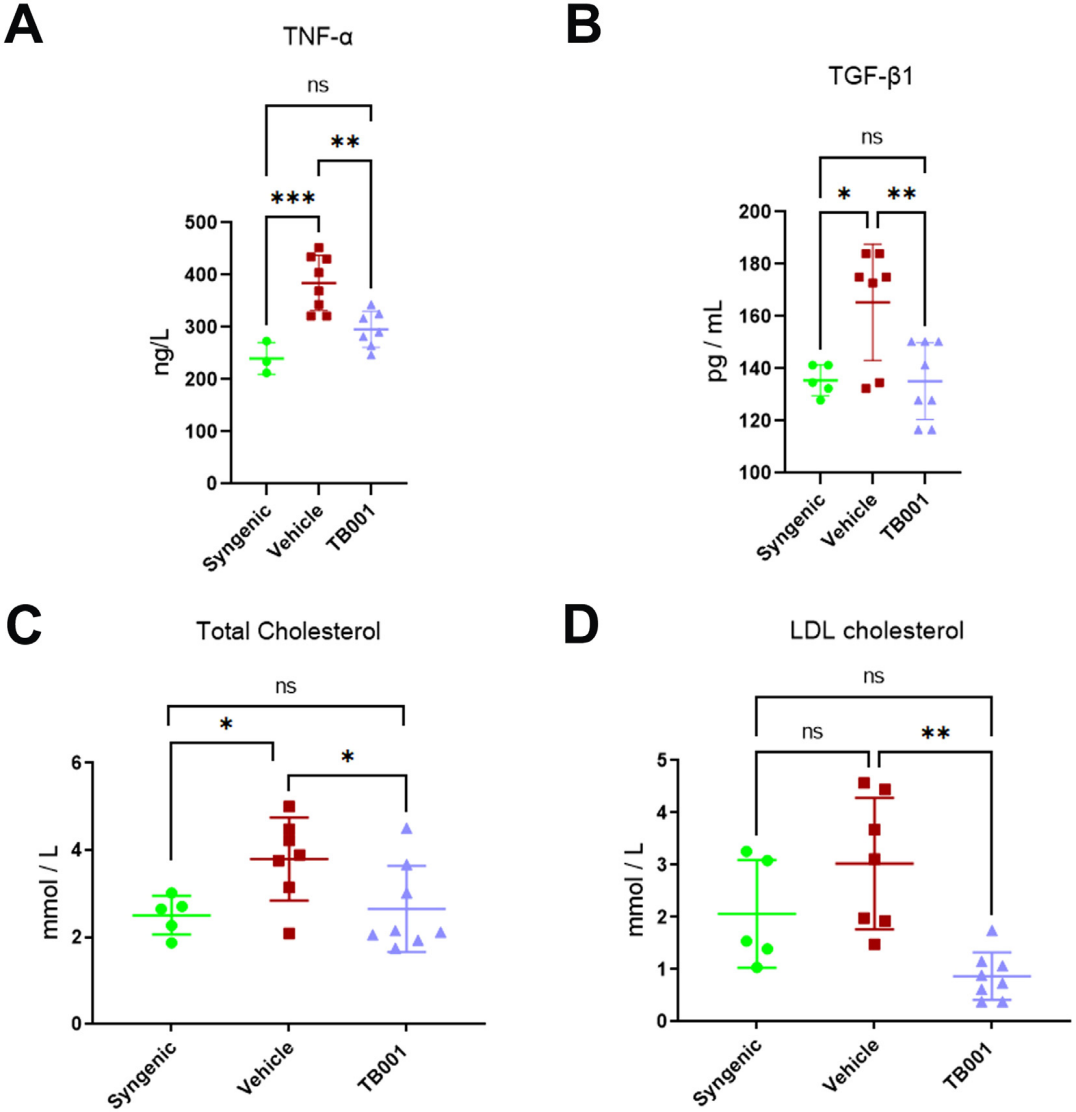
TB001 treatment decreased serum inflammatory cytokines and cholesterol levels at 14^th^ week post-kidney transplant. **(A)** Levels of TNF-α among different groups. **(B)** Levels of TNF-β1 among different groups. **(C)** Levels of total cholesterol among different groups. **(D)** Levels of LDL cholesterol among different groups. ns *P >*0.05, *P <0.05, **P <0.01, ***P < 0.001, One-way ANOVA followed by Tukey’s *post-hoc* test.

Despite the undetermined mechanisms, chronic inflammation and metabolic syndrome are often associated (29). To determine the potential effects of TB001 in inflammatory responses and lipid regulation in transplant recipients, serum inflammatory cytokines and lipid profiles were measured at 14^th^ week post-transplant. Compared with the control group, TB001-treated recipients showed decreased serum TNF-α (*P* = 0.004, **Figure 2A**) and TGF-β1 levels (*P* = 0.007, **Figure 2B**). No significant differences were observed in IL-1β, IL-4, and IL-6 levels between these two groups (**Supplementary Figures 2A–C**). Strikingly, TB001 significantly decreased serum total cholesterol (*P* = 0.04, **Figure 2C**) and LDL cholesterol levels (*P* = 0.001, **Figure 2D**). No significant differences were observed in triglyceride and HDL cholesterol levels (**Supplementary Figures 2D, E**). These data demonstrate the anti-inflammatory and cholesterol-lowering effects of TB001.

### 3.3 TB001 mitigated kidney allograft fibrosis and diminished inflammatory cell infiltration

**Figure 3:**
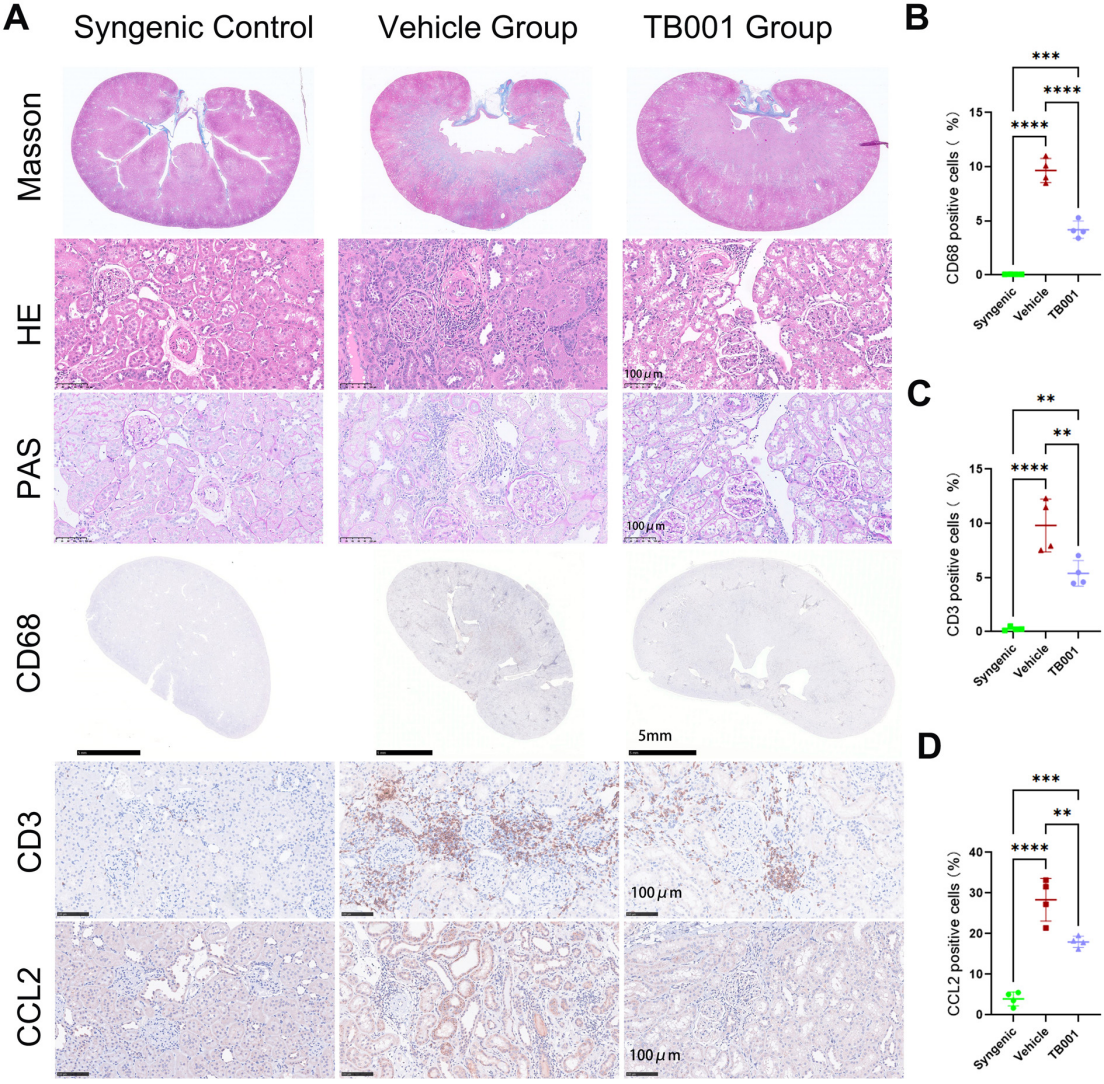
TB001 mitigated kidney allograft fibrosis and diminished inflammatory cell infiltration. **(A)** Representative histological images of kidney grafts from three different groups. **(B)** Quantitative analysis of the ratio of CD68 positive cells. **(C)** Quantitative analysis of the ratio of CD3 positive cells. **(D)** Quantitative analysis of the ratio of CCL2 positive cells. **P <0.01, ***P < 0.001, ****P < 0.0001, One-way ANOVA followed by Tukey’s *post-hoc* test.

Next, histological analysis of kidney grafts was performed to determine the anti-fibrotic effects of TB001. As showed in **Figure 3A**, the vehicle group exhibited extensive multiple and focal inflammatory cell infiltration (by H&E staining), multiple atrophic tubules (by PAS staining), and multiple fibrotic areas (by Masson’s trichrome staining). The pathological characteristics of the vehicle group were consistent with the clinical definitions of IF/TA and i-IFTA based on the Banff classification. In contrast to the vehicle group, the TB001-treated group exhibited markedly reduced infiltration of inflammatory cells and fibrotic areas. IHC staining further confirmed a decrease in CD68+ (macrophages) and CD3+ (T lymphocytes) cell infiltration in the TB001-treated group compared to the vehicle group (**Figures 3B, C**). Moreover, TB001 treatment decreased the expression of the chemokine CCL2 in local tubular epithelial cells (TECs), demonstrating its anti-inflammatory potential (**Figure 3D**).

### 3.4 Transcriptome sequencing of kidney grafts revealed that TB001 treatment improved lipid metabolism pathways

**Figure 4:**
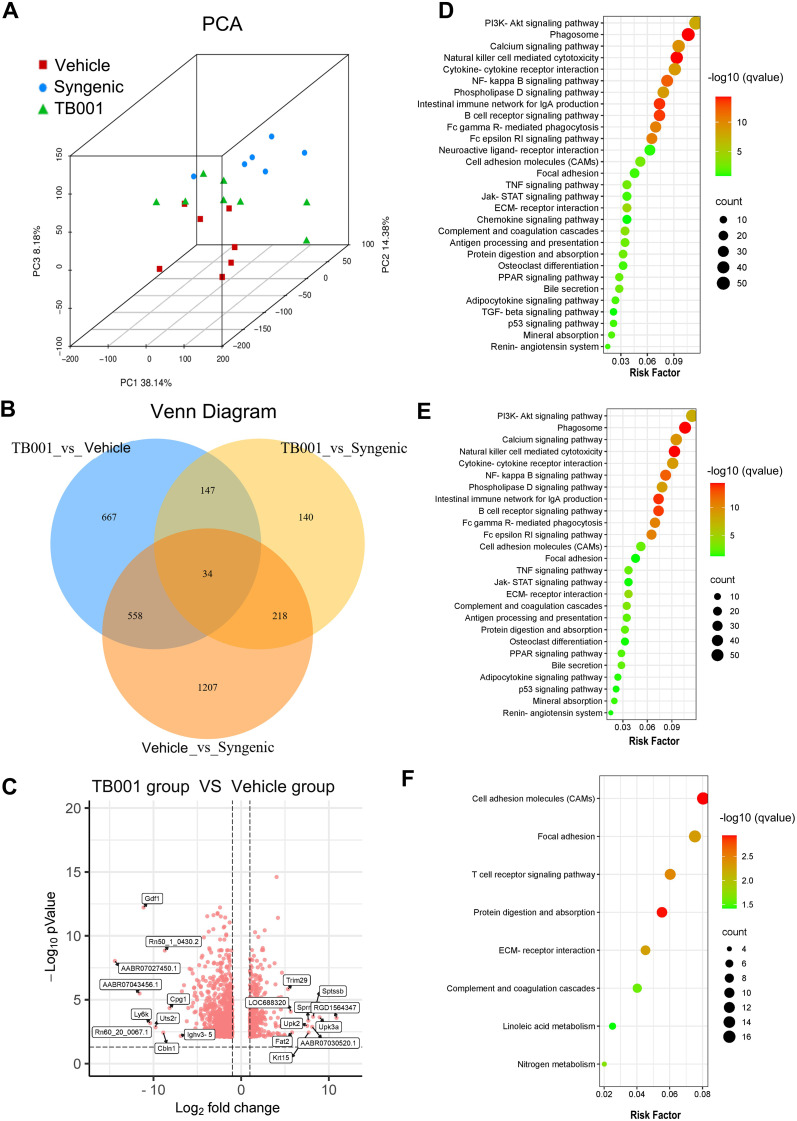
Transcriptome sequencing analysis of kidney grafts. **(A)** Principal component analysis. **(B)** Venn diagram of differentially expressed genes (DEGs) between three groups. **(C)** Heatmap of DEGs. Each row in the figure represents a gene, and each column represents a comparison group. Red color indicates upregulated expression, green color indicates downregulated expression. The darker the color, the higher the expression of differences. **(D)** KEGG enrichment analysis between the chronic fibrosis group the syngenic group. **(E)** KEGG enrichment analysis between the TB001 group the chronic fibrosis group. **(F)** KEGG enrichment analysis between the TB001 group the syngenic group.

Principal component analysis, Venn diagram, and heat map analyses demonstrated that gene expression profiles of TB001-treated samples were more similar to those of the syngeneic control samples (**Figures 4A, B**). Volcano plots comparing the TB001-treated group with the vehicle-treated group showed 494 up-regulated transcripts and 955 down-regulated transcripts (**Figure 4C**). KEGG enrichment analysis comparing the vehicle-treated group and syngeneic control group revealed significant enrichment of several immune-related pathways. Pathways related to fibrosis and EMT were also enriched, including focal adhesion, extracellular matrix-receptor interaction, and cell adhesion molecules. Interestingly, lipid metabolism pathways, such as the phospholipase D signaling pathway and PPAR signaling pathway, were significantly enriched (**Figure 4D**). Similar pathway enrichment was observed when comparing the vehicle-treated group with the TB001-treated group (**Figure 4E**). However, these pathways were significantly less enriched when comparing the TB001-treated group with the syngeneic control, and the PPAR signaling and phospholipase D signaling pathways were no significant enriched (**Figure 4F**). These results suggest that inflammatory responses and lipid metabolism were closely involved in kidney allograft fibrosis, and TB001 treatment appears to improve lipid metabolism, reduce inflammatory response, and alleviate kidney allograft fibrosis.

### 3.5 TB001 treatment alleviated EMT process in kidney allografts

**Figure 5:**
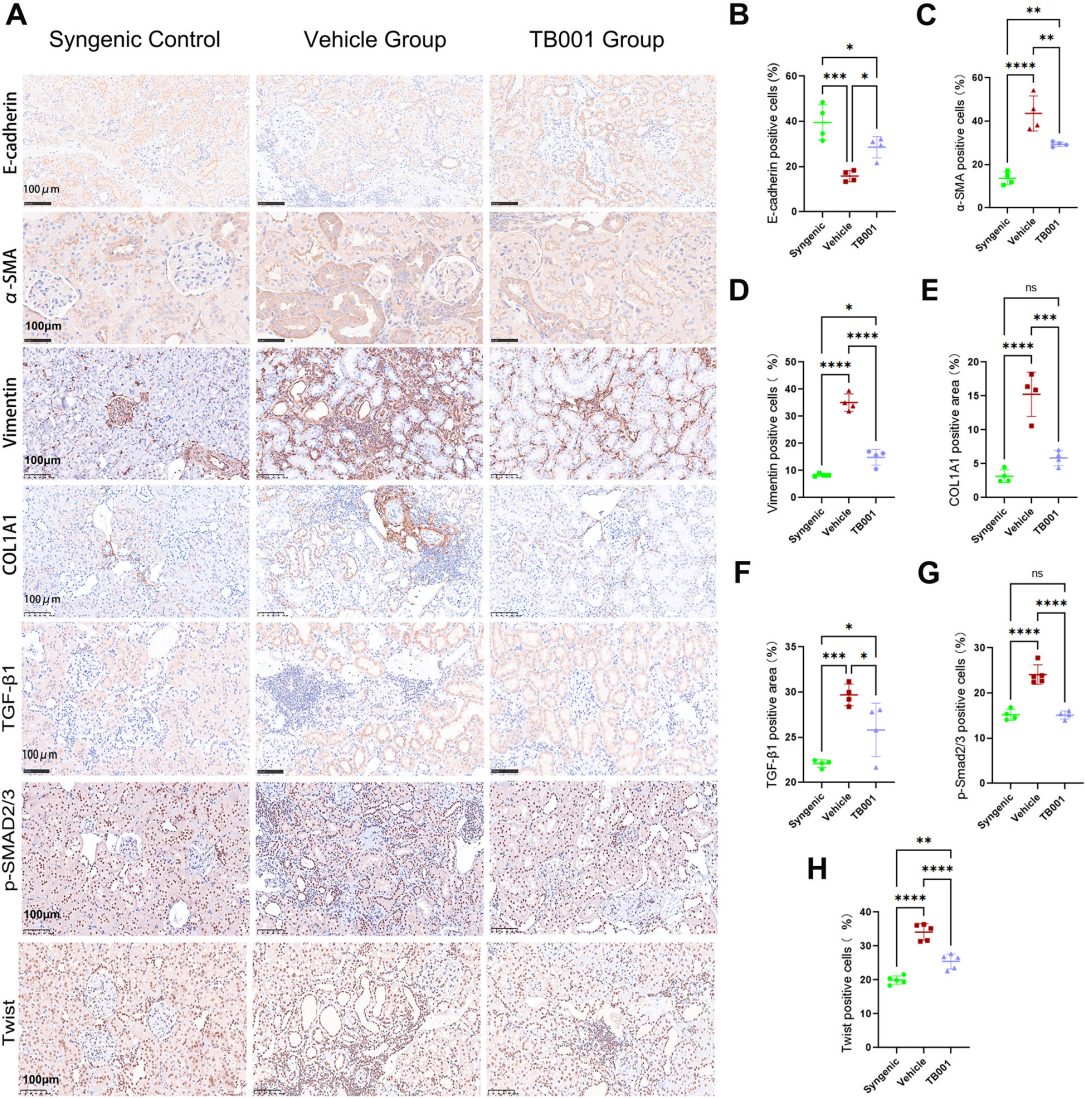
Immunohistochemical assay of EMT markers and TGF-β1 pathway. **(A)** Representative IHC images of E-cadherin, α-SMA, Vimentin, COL1A1, TGF-β1, p-Smad2/3 and Twist staining. **(B)** Quantitative analysis of E-cadherin positive cells. **(C)** Quantitative analysis of α-SMA positive cells. **(D)** Quantitative analysis of vimentin positive cells. **(E)** Quantitative analysis of COL1A1 positive cells. **(F)** Quantitative analysis of TGF-β1 positive cells. **(G)** Quantitative analysis of p-Smad2/3 positive cells. **(H)** Quantitative analysis of Twist positive cells. ns P >0.05, *P <0.05, **P <0.01, ***P < 0.001, ****P < 0.0001, One-way ANOVA followed by Tukey’s *post-hoc* test.

Epithelial-mesenchymal transition is commonly observed during kidney allograft fibrosis. To determine if TB001 treatment could alter EMT process, mRNA and protein expression levels of related molecules were tested. As shown In **Supplementary Figure 3**, qPCR analysis demonstrated that TB001 significantly reduced the mRNA expression levels of fibronectin (FN), α-smooth muscle actin (α-SMA), and vimentin compared with the vehicle-treated group. These findings suggest that TB001 effectively attenuated the EMT process in kidney allografts. Gene expression levels of protein kinase C-α (PKC-α), PKC-β, Snail1, and Twist were elevated in both vehicle- and TB001-treated groups. IHC analysis in **Figure 5A** confirmed that the expression of EMT protein markers corresponded with their mRNA levels. Specifically, E-cadherin expression was significantly decreased in the vehicle group (**Figure 5B**), while α-SMA, vimentin, and type I collagen (COL1A1) expression were markedly increased (**Figures 5C–E**). Moreover, TGF-β1 expression was notably concentrated along the brush border of renal tubules in the vehicle-treated group compared to the TB001-treated group (**Figure 5F**). The ratio of p-Smad2/3 positive TECs was significantly higher in the vehicle-treated group (**Figure 5G**), as was the ratio of Twist-positive TECs (**Figure 5H**). These results suggest that TB001 can alleviate EMT process and kidney allograft fibrosis.

### 3.6 TB001 increased expression of CPT1A and decreased expression of PKC-β

**Figure 6:**
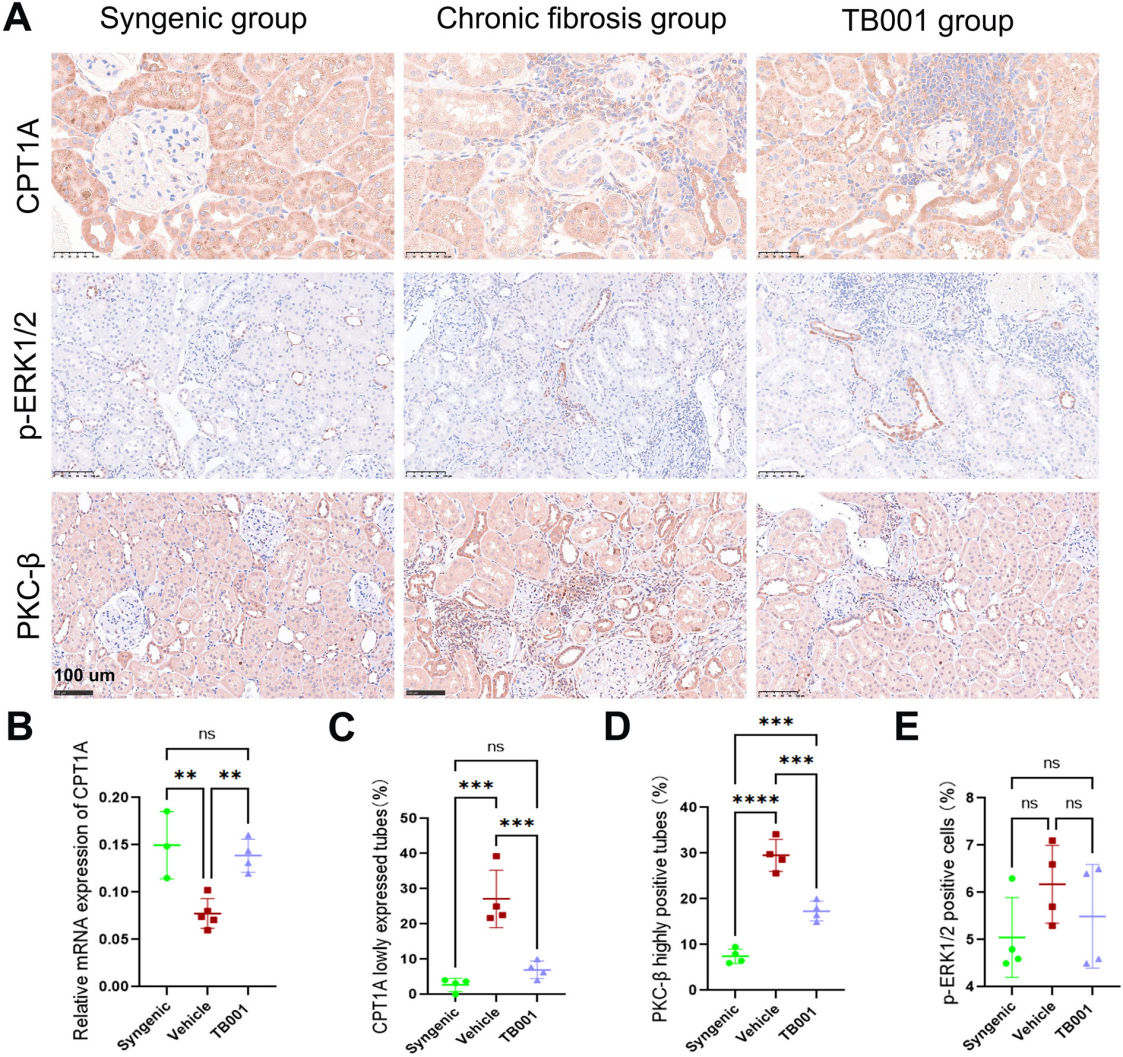
TB001 increased CPT1A expression and decreased PKC-β expression. **(A)** Representative IHC images of CPT1A, p-ERK1/2 and PKC-β staining. **(B)** Quantitative analysis of CPT1A mRNA expression. **(C)** Quantitative analysis of CPT1A positive tubes. **(D)** Quantitative analysis of PKC-β highly positive tubes. **(E)** Quantitative analysis of p-ERK1/2 positive cells.ns *P* > 0.05, **P < 0.01, ***P < 0.001, ****P < 0.0001, One-way ANOVA followed by Tukey’s *post-hoc* test.

Given the strong effects of TB001 in fibrosis inhibition and lipid regulation, we further investigated how it regulates lipid metabolism. carnitine palmitoyltransferase 1A (CPT1A) is a crucial enzyme that facilitates the breakdown of long-chain fatty acids in mitochondria for energy production and plays a key role in lipid metabolism by catalyzing the transport of fatty acids into the mitochondrial matrix, where they undergo beta-oxidation to generate energy. As shown in **Figures 6A, B**, TB001 treatment significantly increased the expression of CPT1A at both mRNA and protein levels. IHC staining of kidney grafts revealed significantly decreased CPT1A levels in area with i-IFTA (**Figure 6C**). Conversely, protein expression of PKC-β in the i-IFTA areas was also significantly increased, particularly in the vehicle-treated group (**Figure 6D**). These results suggest that TB001 promotes CPT1A expression, improves lipid metabolism, and reduces PKC-β expression in kidney allografts. However, no statistically significant difference was observed in p-ERK1/2 protein expression (**Figure 6E**), indicating that the ERK1/2 pathway may not be a primary mediator in kidney allograft fibrosis.

### 3.7 *In vitro* experiments confirmed that TB001 inhibited PKC and TGF-β1/Smad pathways and alleviated EMT process

**Figure 7:**
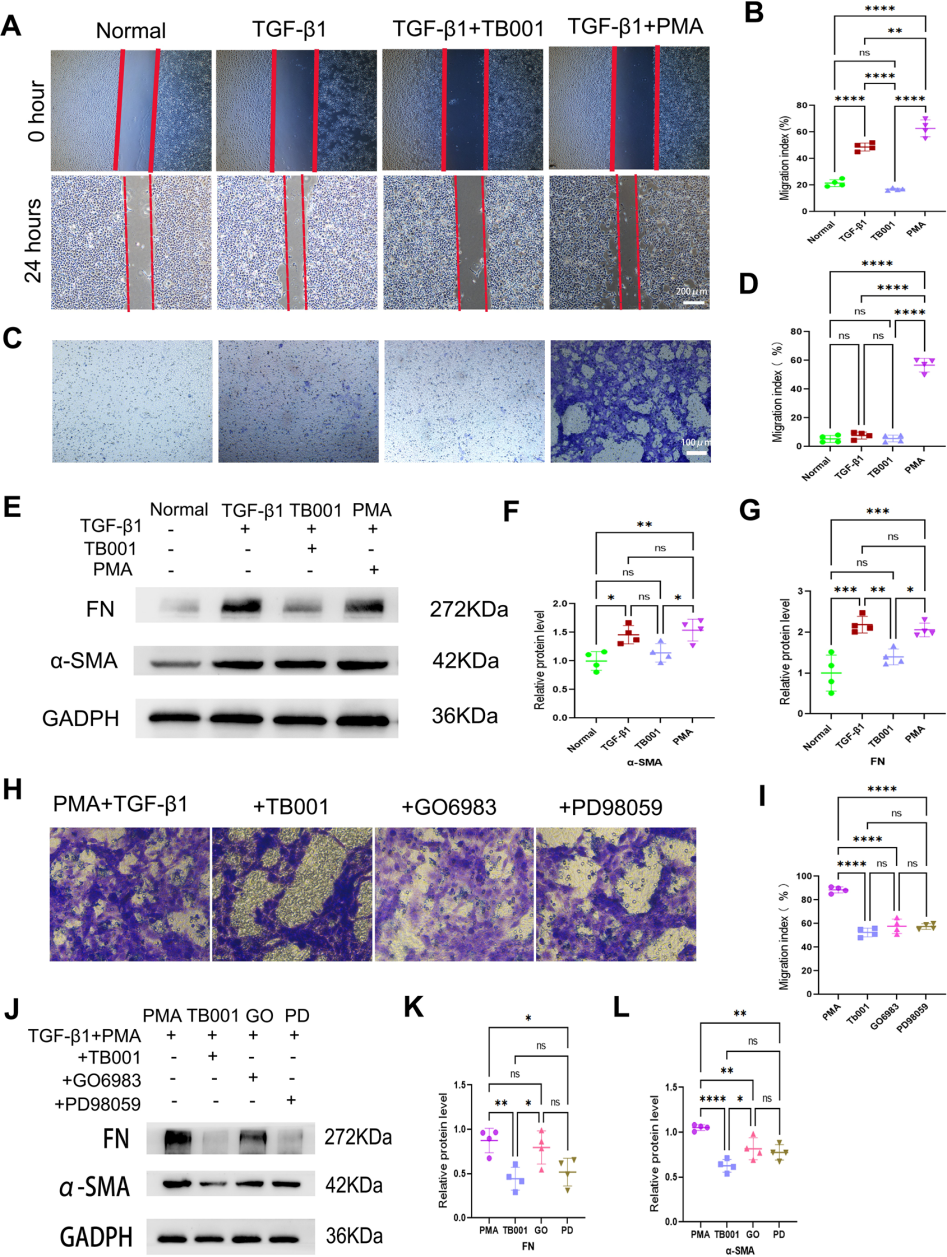
TB001 inhibited EMT process *in vitro*. **(A)** Scratch assay of HK-2 cells induced by TGF-β1. **(B)** Quantitative analysis of HK-2 cells migration in **Figure 5A**. **(C)** Transwell assay of HK-2 cells induced by TGF-β1. **(D)** Quantitative analysis of HK-2 cells migration **Figure 5C**. **(E)** WB assay of FN and α-SMA in the model of HK-2 cells induced by TGF-β1. **(F, G)** Quantitative analysis of WB assay in **Figure 5E**. **(H)** Transwell assay of HK-2 cells induced by TGF-β1 combined with PMA (PKC agonist). **(I)** Quantitative analysis of HK-2 cells migration **Figure 5H**. **(J)** WB assay of FN and α-SMA in the model of HK-2 cells induced by TGF-β1 combined with PMA (PKC agonist). **(K, L)** Quantitative analysis of WB assay in **Figure 5J**. ns *P* > 0.05, *P <0.05, **P < 0.01, ***P < 0.001, ****P < 0.0001, One-way ANOVA followed by Tukey’s *post-hoc* test.

*In vitro* experiments were carried out using HK-2 cells to validate our findings. As shown in **Figures 7A, B**, TB001 effectively inhibited HK-2 cells migration and EMT process induced by TGF-β1, whereas the PKC agonist PMA significantly promoted cell migration. In the transwell assay, cell motility was significantly increased upon PMA treatment (**Figures 7C, D**). Both WB (**Figures 7E–G**) and IF assays (**Supplementary Figure 4**) demonstrated that TB001 reduced expression of α-SMA and FN, while PMA increased their expression levels. Furthermore, TB001, PKC inhibitor GO6983, and MEK inhibitor PD98059 significantly inhibited HK-2 cell migration induced by TGF-β1 and PMA (**Figures 7H, I**). Inhibition of the ERK1/2 pathway by PD98059 also ameliorated the EMT process induced by PKC activation and TGF-β1 stimulation. These findings were further corroborated by both Western blot (**Figures 7J–L**) and immunofluorescence assays (**Supplementary Figure 5**).

**Figure 8:**
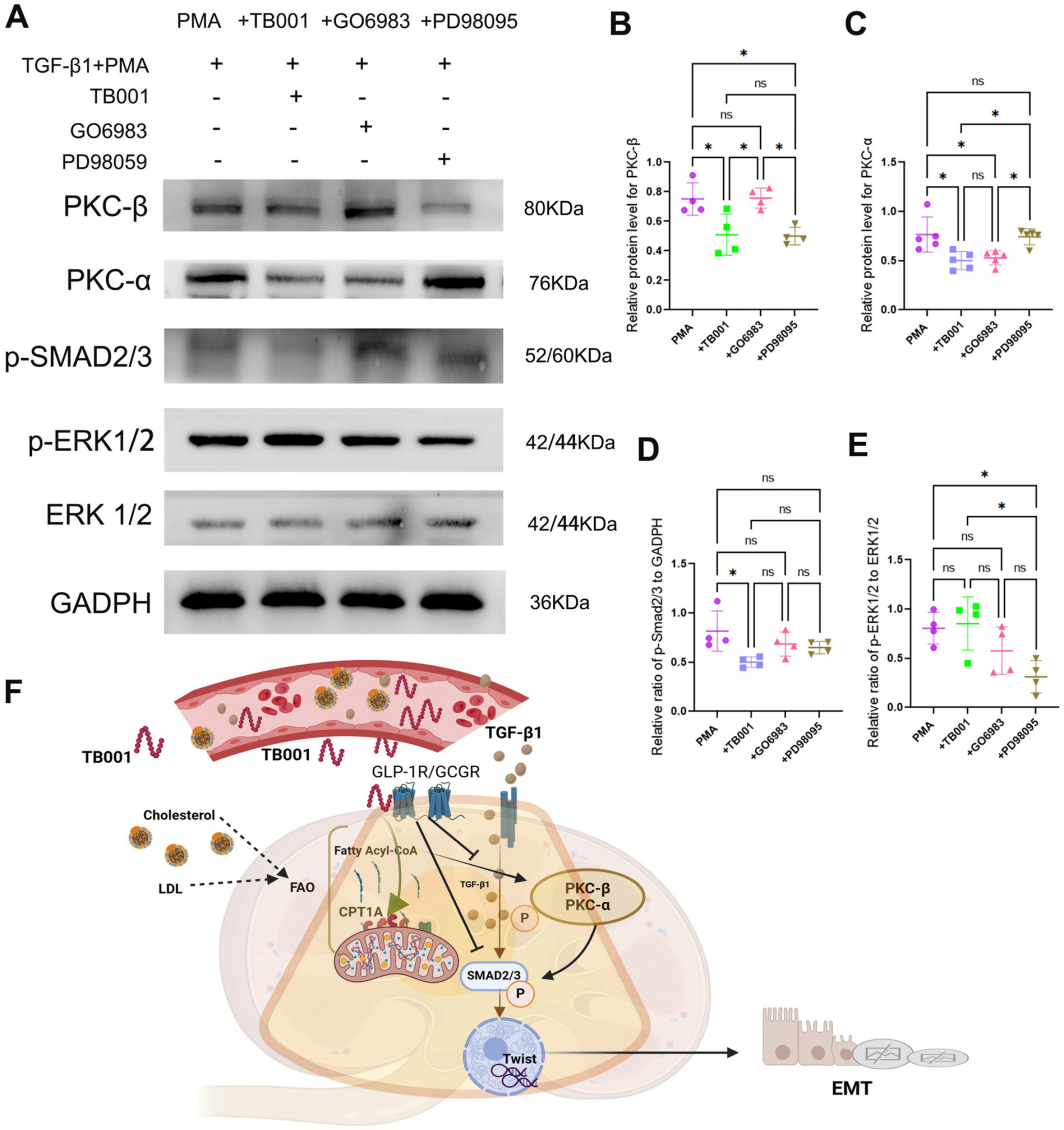
TB001 inhibited EMT process by inhibiting TGF-β1/p-Smad2/3, PKC-α and PKC-β pathway. **(A)** Represented images of WB assay. **(B)** Quantitative analysis of PKC-β protein level. **(C)** Quantitative analysis of PKC-α protein level. **(D)** Quantitative analysis of p-Smad2/3 protein level. **(E)** Quantitative analysis of p-ERK1/2 protein level. **(G)** Major mechanisms of TB001 acting on transplanted kidney fibrosis. ns *P* > 0.05, *P <0.05, One-way ANOVA followed by Tukey’s *post-hoc* test.

TB001 and PD98059 significantly reduced the expression of PKC-β (**Figures 8A, B**), while TB001 and GO6983 significantly reduced PKC-α expression levels (**Figure 8C**). Additionally, TB001 significantly inhibited the expression of p-Smad2/3, but no significant effects were observed with GO6983 or PD98059 treatment (**Figure 8D**). These data suggest that TB001 reduces HK-2 cell fibrosis by inhibiting both PKC-α and PKC-β pathways, as well as the TGF-β1/Smad2/3 pathway. Interestingly, while inhibition of MEK by PD98059 effectively suppressed ERK1/2 activation, reduced p-ERK1/2 expression levels, and alleviated the EMT process, TB001 did not affect ERK1/2 signaling pathway (**Figure 8E**), aligning with the immunohistochemistry results in kidney grafts.

## 4 Discussion

In this study, using clinically relevant rat kidney transplant models, we demonstrated that TB001 exerts multiple beneficial effects on graft outcomes, including reducing fibrosis and improving graft function. We further determined that the underlying mechanisms of the protective effects by TB001 involve enhancing lipid metabolism related pathways and inhibiting key pro-fibrotic pathways, such as TGF-β1/Smad2/3/Twist and PKC signaling. These findings were further supported by *in vitro* studies showing TB001’s effectiveness in mitigating the epithelial-mesenchymal transition (EMT) process.

In recent years, ECDs have been used more frequently to expand the donor pool, and the survival rate of kidneys from ECDs is significantly lower than that of organs from standard criteria donors (SCDs) or living donors (30, 31). Metabolism syndrome (MS) is prevalent among ECDs and kidney recipients, and MS and CAD significantly decreased the long-term kidney allograft survival (12, 13, 32). To treat the kidney allograft fibrosis, treatments involved in the lipid metabolism seems promising. In CKD patients, diabetic nephropathy, has been identified as the leading cause of end-stage renal disease (33). Regulating blood glucose and lipid metabolism have been identified as key signaling pathways to treat or prevent diabetic nephropathy (32, 34). And given that increasing evidences showing the critical role of lipid metabolism in kidney allograft fibrosis, therapeutic approaches targeting MS could potentially mitigate kidney allograft fibrosis.

TB001 in the present study, which reduced body weight and improved serum lipid levels in rats following kidney transplantation. This finding is consistent with previous study in both clinical studies, as well as basic researches. GLP-1R is widely distributed throughout the body, including the pancreas, central nervous system, gastrointestinal tract, cardiovascular system, and adipose tissue (21). GLP-1R agonists have demonstrated significant efficacy in the treatment of type 2 diabetes and obesity (35). And Recent studies have indicated that GLP-1R signaling exerts protective effects against atherosclerosis and endothelial dysfunction, along with anti-inflammatory effects on macrophages and anti-proliferative effects on smooth muscle cells (36). Thus, the combined activation of GLP-1R and GCGR may enhance the treatment of MS (20, 37), as well as in the setting of kidney transplantation.

In our previous studies, we highlighted that abnormal lipid metabolism is closely associated with kidney allograft fibrosis, and we found CPT1A is a critical target in treatment of kidney allograft fibrosis (7). Previous metabolomic and transcriptomic study of Cotadutide (GLP-1R/GCGR dual agonist) in hepatic fibrosis identified its effects on pathways related to lipogenesis, fibrosis, and inflammation (20). It proves the effectiveness of GLP-1R/GCGR dual agonist in anti-fibrosis under setting of non-transplantation. Similarly, transcriptome sequencing in the present study revealed that TB001 improves lipid metabolism-related pathways, such as the PPAR signaling pathway, in transplanted kidneys. IHC and qPCR assays further demonstrated that TB001 significantly increased the CPT1A expression, a key enzyme in lipid metabolism. The expression of CPT1A in both mRNA level and protein level is consistent with transcriptome sequencing findings. In the present study, we further found that TB001, dual GLP-1R and GCGR agonist, improve the lipid metabolism in the transplant kidney. It not only presented by the better serum lipid level and body weight control, but also showed in the better lipid metabolic and less kidney allograft fibrosis.

To date, research on GLP-1R/GCGR agonists in the treatment of kidney fibrosis remains limited, and the effects of GLP-1R or GCGR agonists on kidney allograft fibrosis have not been investigated. Previously, the GLP-1R agonist liraglutide has been shown to attenuate EMT changes in the UUO model by inhibiting TGF-β1/Smad3 and ERK1/2 signaling pathways (23). To better study the potential pathway of TB001 in improving intrarenal lipid metabolism. EMT model in HK-2 cells induced by TGF-β1 and PMA was applied, and this model exhibited dysregulated lipid metabolism and increased PKC expression. Previous studies have suggested that PKC-α mediates epidermal growth factor receptor (EGFR) ubiquitination in podocytes, cell surface endocytosis, and subsequent extracellular signal-regulated kinase (ERK) activation during hyperglycemia, leading to podocyte injury (38). Currently, the activation of PKC-α is widely recognized as a key pathological mechanism in the development of diabetic nephropathy (39, 40). In an experimental model of diabetic nephropathy, rubositoline has been shown to normalize glomerular hyperfiltration by inhibiting PKC-β, reduce urinary albumin excretion, maintain renal function, and mitigate mesangial dilation, glomerular sclerosis, and tubulointerstitial fibrosis. PKC-β inhibition is considered a promising strategy for improving the prognosis of diabetic nephropathy (41). These findings suggest that activation of PKC-α and PKC-β in kidney is associated with abnormal fatty acid oxidation in both non-transplantation and transplantation setting.

The present study in kidney transplantation model also indicates that TB001 significantly enhances lipid metabolism (marked by increased CPT1A expression in TECs) and reduces PKC-α and PKC-β expression, thereby attenuating kidney allograft fibrosis. TB001 also inhibits the TGF-β1/Smad2/3/Twist pathway, further mitigating renal fibrosis. These findings were also supported by *in vitro* results showing that TB001 reduced EMT changes and fibrosis in HK-2 cells by inhibiting PKC-α, PKC-β, and the TGF-β1/Smad2/3 pathway. Notably, TB001 did not inhibit ERK1/2 activation, suggesting that its effects on kidney allograft fibrosis do not involve ERK1/2 pathway.

Although the animal model used in this study reduced the risk of acute rejection, it failed to fully simulate the long-term immunosuppressive state of clinical transplant recipients, which may lead to bias in the evaluation of the fibrosis process of human kidney transplantation. In view of the routine use of immunosuppressants in clinical practice, this study failed to systematically evaluate the potential impact of tacrolimus, mycophenolate mofetil and prednisone. Due to the study design and sample size, this study failed to carry out horizontal comparative analysis of other potential treatment regimens. Although the current experimental results suggest that TB001 plays an important regulatory role in improving the lipid metabolism pathway of transplanted kidney, it has not yet been verified by molecular tools such as conditional gene knockout. The above limitations may have a certain impact on the universality of the conclusion, which needs further improvement in the follow-up study.

## 5 Conclusions

In summary, the GLP-1R/GCGR dual agonist TB001 effectively mitigates kidney allograft fibrosis and improves graft function, likely through mechanisms involving enhanced lipid metabolism and inhibition of the PKC-α, PKC-β, and TGF-β1/SMAD2/3/Twist pathways.

## Data Availability

The raw data supporting the conclusion of this article will be made available at China National Center for Bioinformation (CNCB) database (project number: PRJCA034659).
