# Supplementary material for: GLP-1 and glucagon receptor dual agonism ameliorates kidney allograft fibrosis by improving lipid metabolism
Source: Front Immunol. 2025 Mar 31;16:1551136. doi: 10.3389/fimmu.2025.1551136 (PMC11994718; doi:10.3389/fimmu.2025.1551136)
Supplement: Supplementary file 1 [file DataSheet1.pdf]

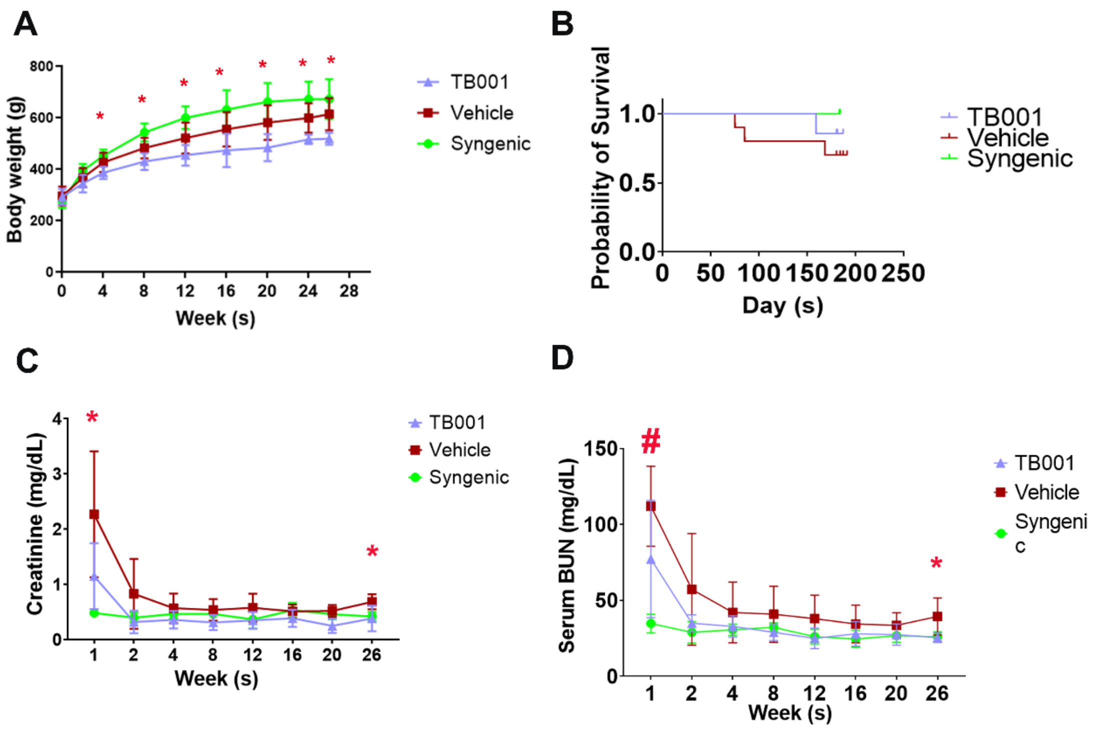

Supplementary figure 1. TB001 improved outcomes of SD-Wistar rat kidney transplantation model.

(a) Levels of body weight at different time points of the three groups were shown. (b) Survival analysis of TB001 in SD-Wistar rat kidney transplantation model and in a 26-weeks observation periods. Levels of blood creatinine(c), blood urea nitrogen (BUN) (d) at different time points of the three groups were shown. *Difference shown between the vehicle group VS. the TB001 group, $P < 0.05$, unpaired t test.

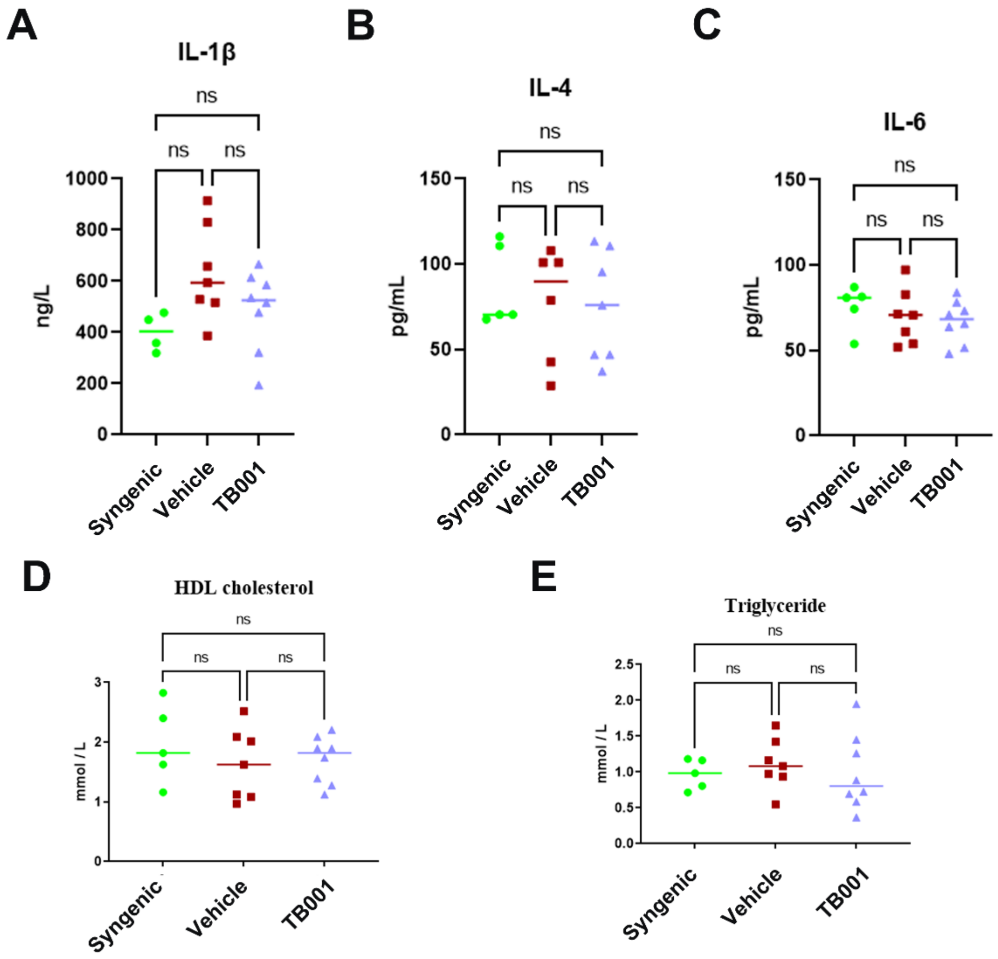

Supplementary figure 2. Levels of Inflammatory factors and serum lipids in F344-Lewis rat kidney transplantation model.

(a) Levels of IL-1β among different groups. (b) Levels of IL-4 among different groups. (c) Levels of IL-6 among different groups. (d) Levels of TNF-α among different groups. (e) Levels of TNF-α among different groups. ns, $P > 0.05$, unpaired t test.

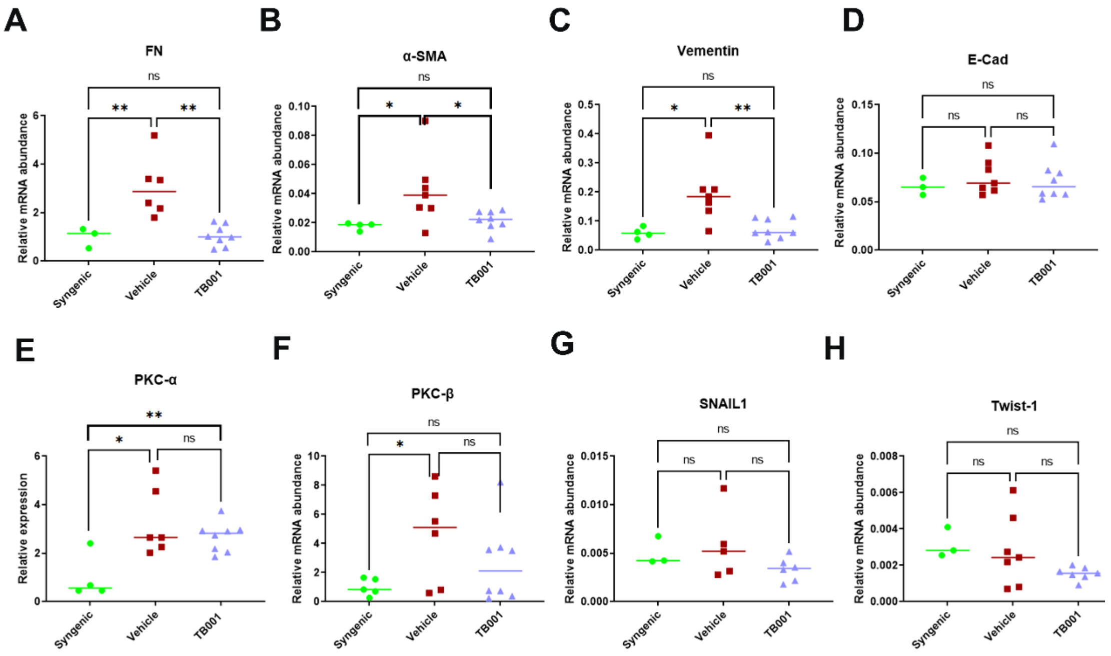

Supplementary figure 3. Results of EMT-related mRNA expression in F344-Lewis rat kidney transplantation model.

(a) Expression of FN among different groups. (b) Expression of α-SMA among different groups. (c) Expression of vimentin among different groups. (d) Expression of E-cadherin among different groups. (e) Expression of PKC-α among different groups. (f) Expression of PKC-β among different groups. (f) Expression of Snail1 among different groups. (d) Expression of Twist among different groups. ns *P* >0.05, **P* <0.05, ** *P* <0.01, unpaired t test.

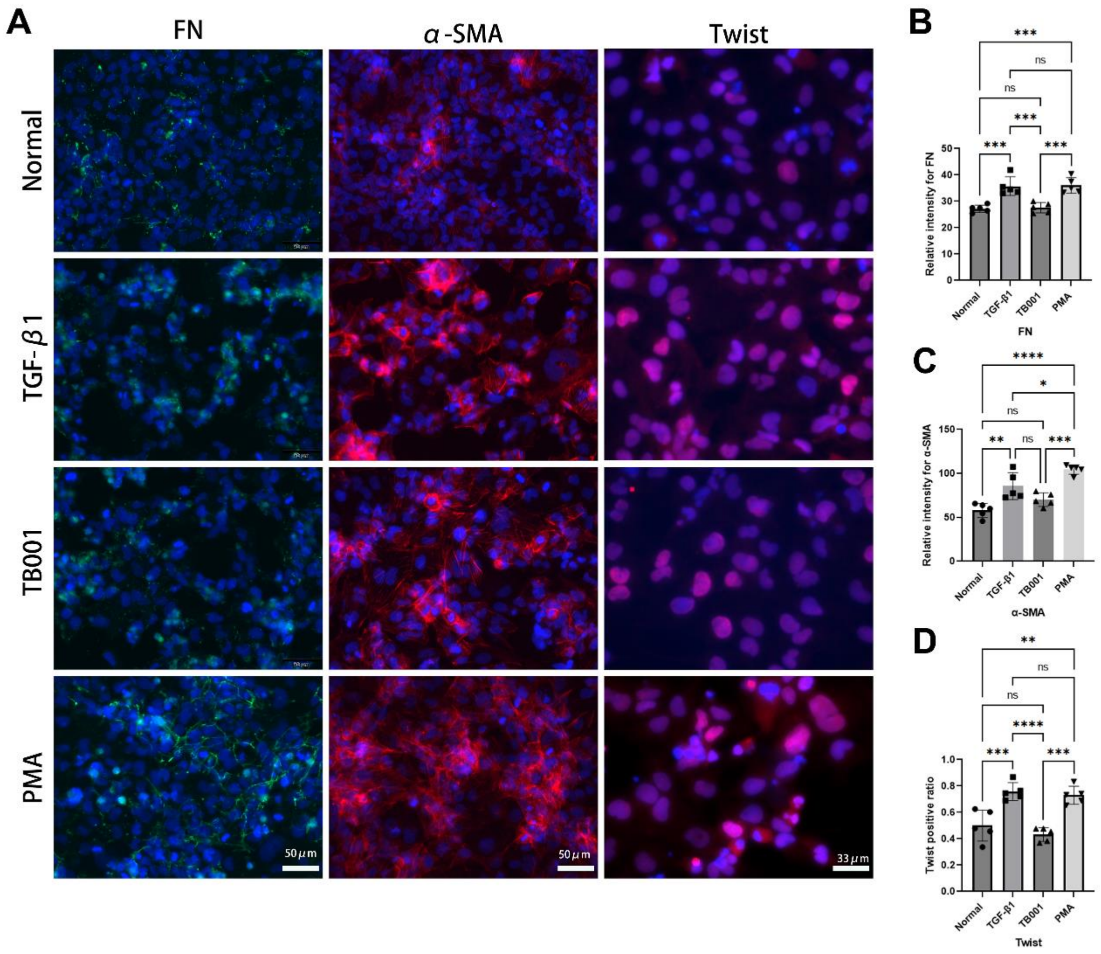

Supplementary figure 4. Representative images of immunoinfluoresence in the model of HK-2 cells induced by TGF-β1.

(a) Representative images of immunoinfluoresence in different groups, including FN, α-SMA and Twist. (b) Expression of FN among different groups. (c) Expression of α-SMA among different groups. (d) Expression of Twist among different groups. ns *P* >0.05, **P* <0.05, ** *P* <0.01, *** *P* <0.001, **** *P* <0.0001, unpaired t test.

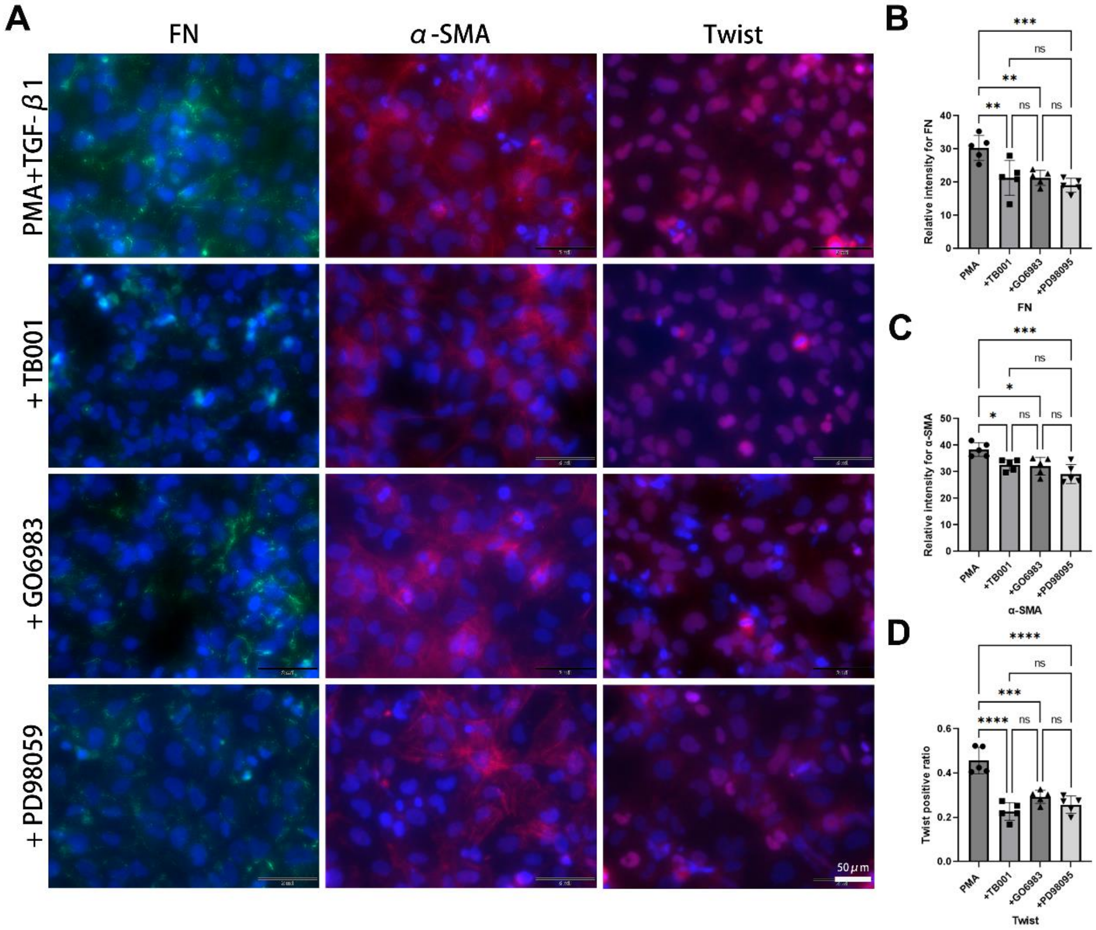

Supplementary figure 5. Representative images of immunoinfluoresence in the model of HK-2 cells induced by TGF-β1 and PMA.

(a) Representative images of immunoinfluoresence in different groups, including FN, α-SMA and Twist. (b) Expression of FN among different groups. (c) Expression of α-SMA among different groups. (d) Expression of Twist among different groups. ns *P* >0.05, **P* <0.05, ** *P* <0.01, *** *P* <0.001, **** *P* <0.0001, unpaired t test.
