# Supplementary material for: GLP-1 and glucagon receptor dual agonism ameliorates kidney allograft fibrosis by improving lipid metabolism
Source: Front Immunol. 2025 Mar 31;16:1551136. doi: 10.3389/fimmu.2025.1551136 (PMC11994718; doi:10.3389/fimmu.2025.1551136)

Western Blot

- Image Software: ChemiScope S6
- Protein markers: MultiColor Prestained Protein Marker (EpiZyme company, cat: WJ102)

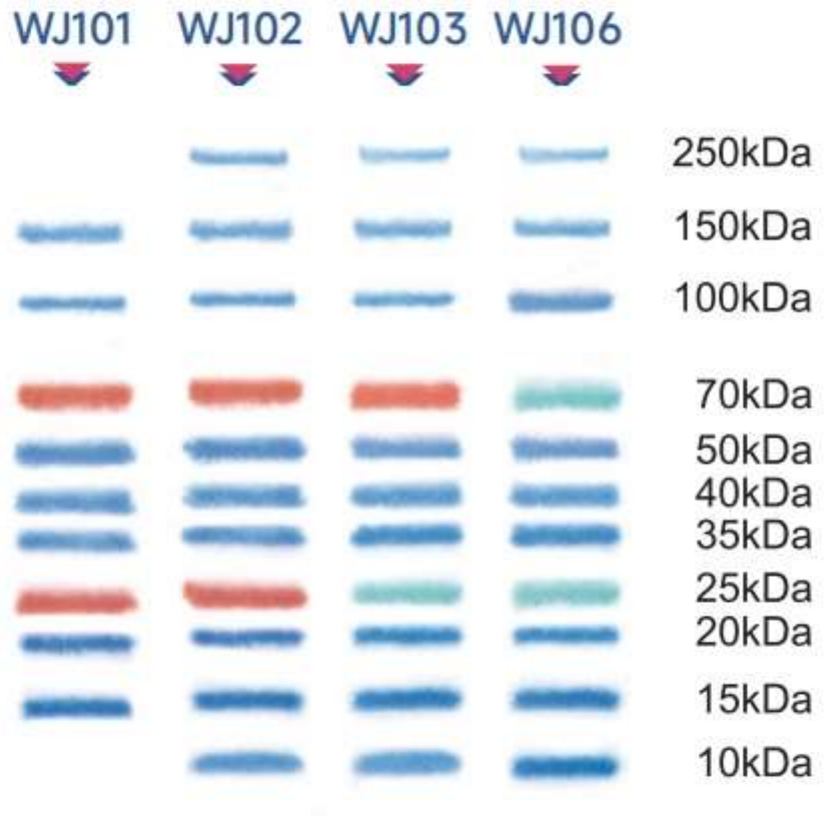

Figure 7.E

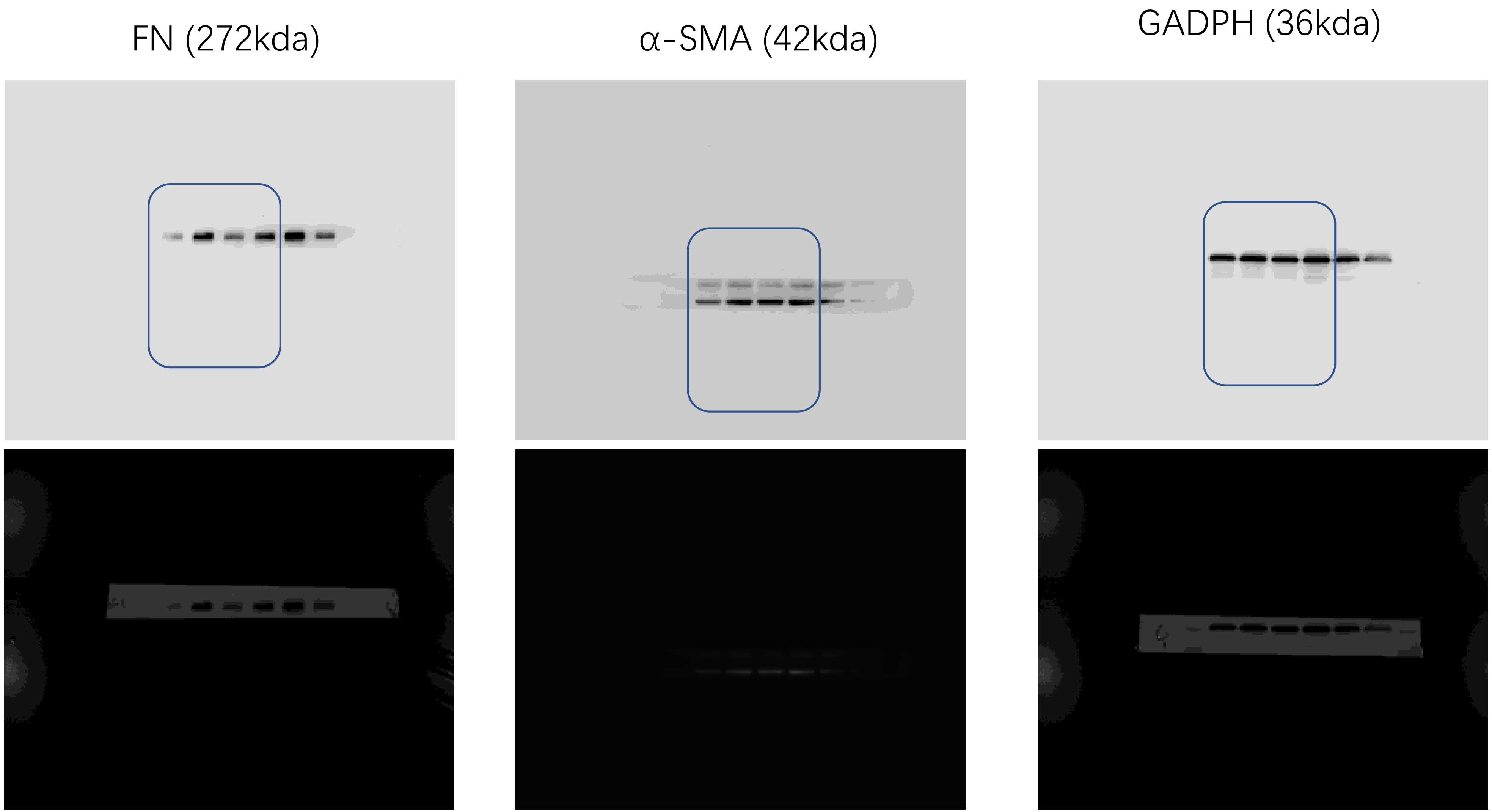

Figure 7.J

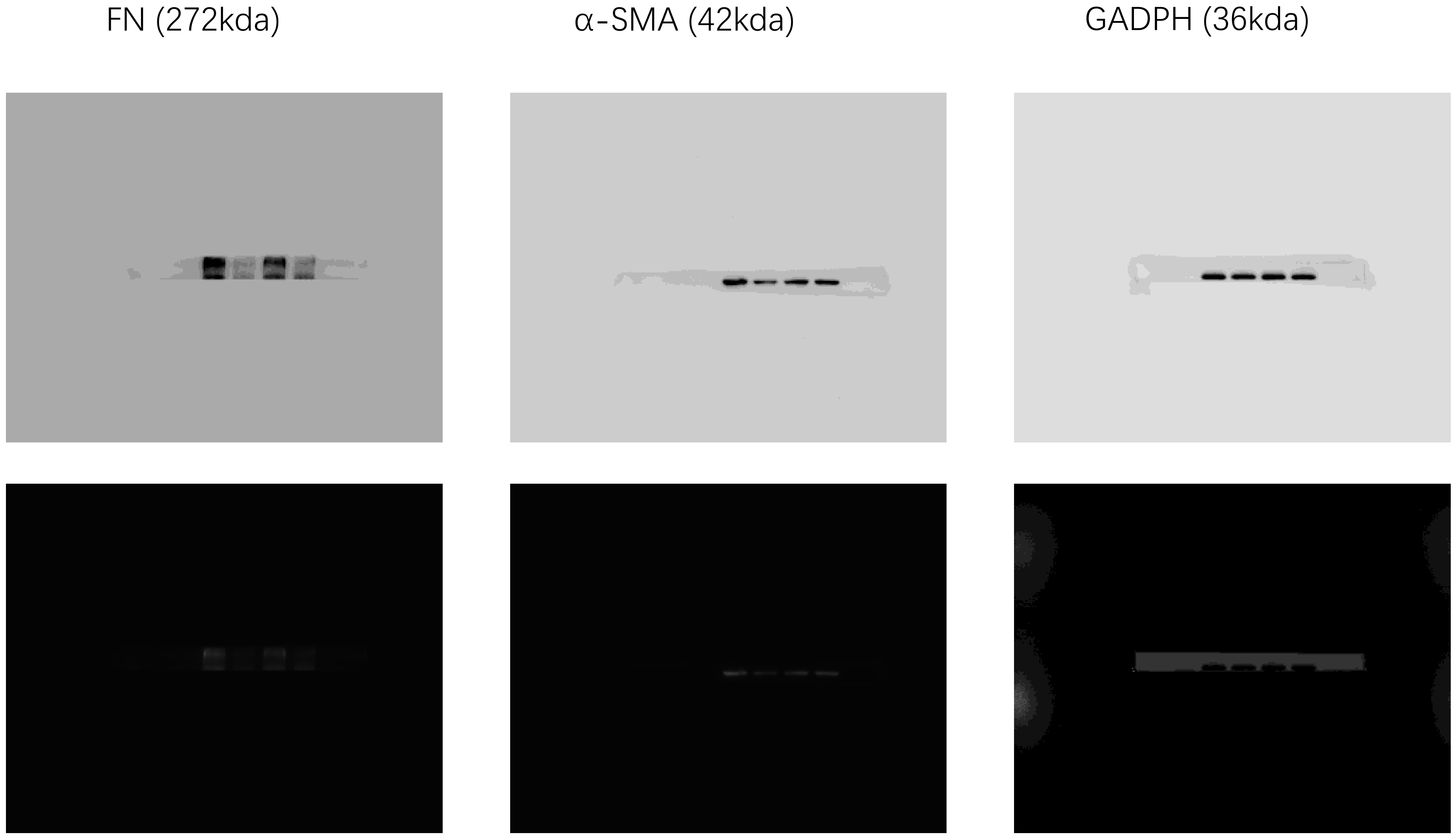

Figure 8 PKC-β

GADPH (36kda)

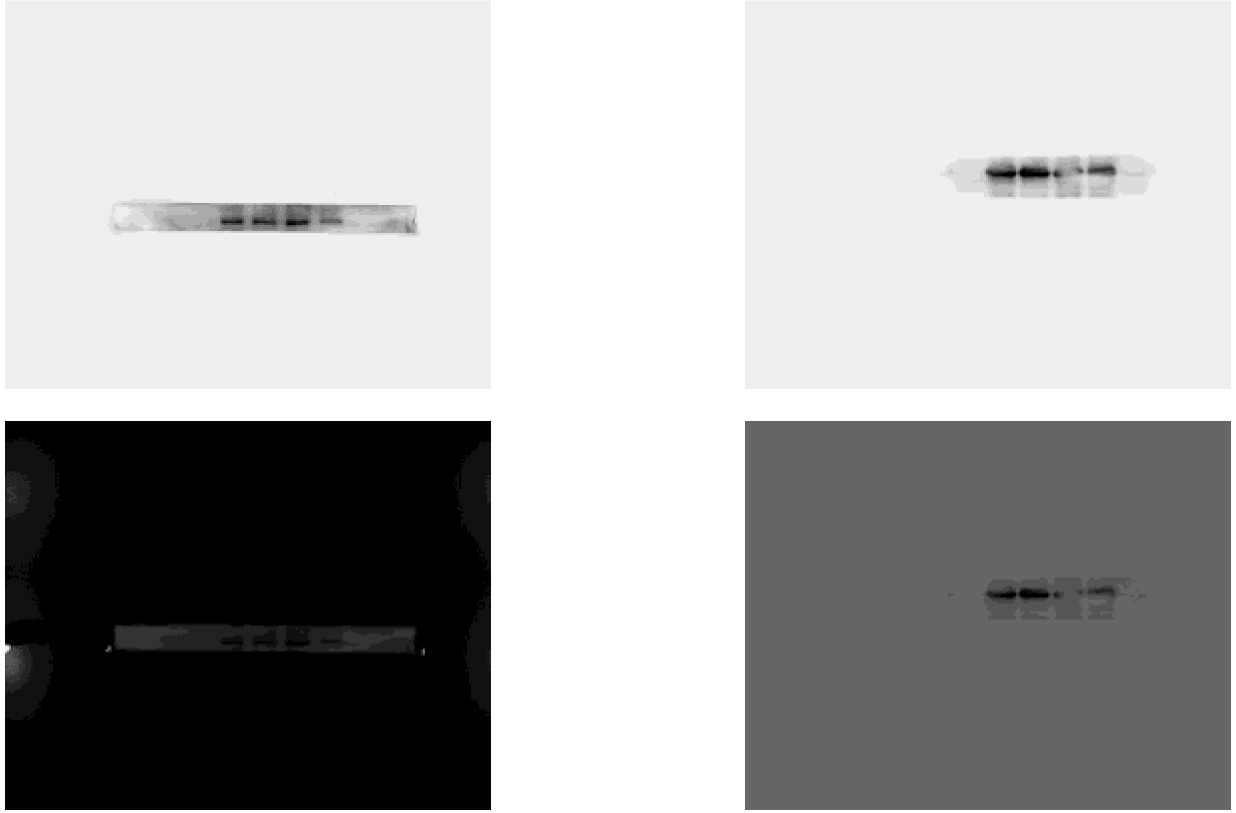

Figure 8 -PKC-α

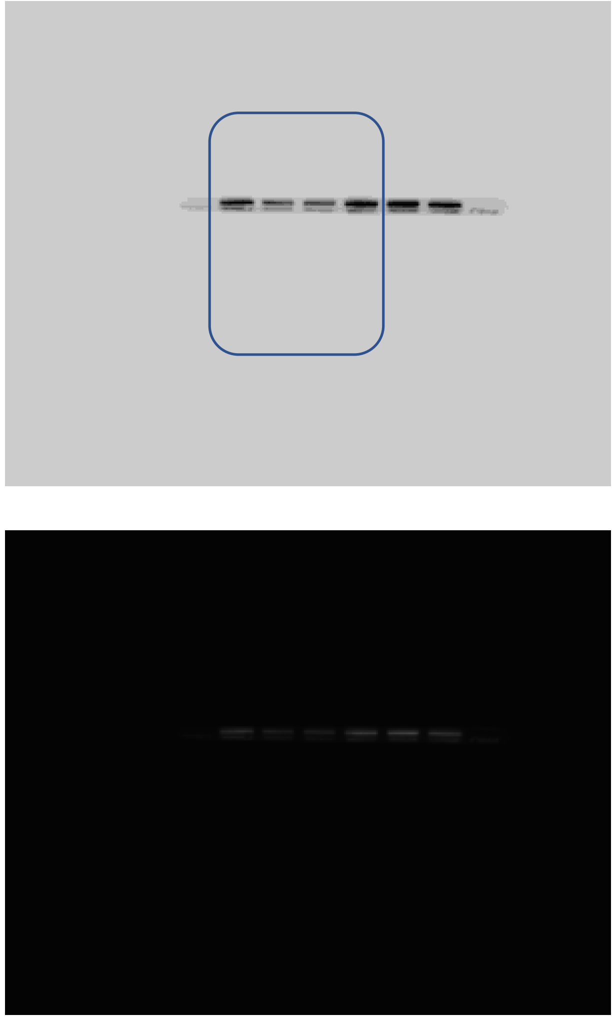

GADPH (36kda)

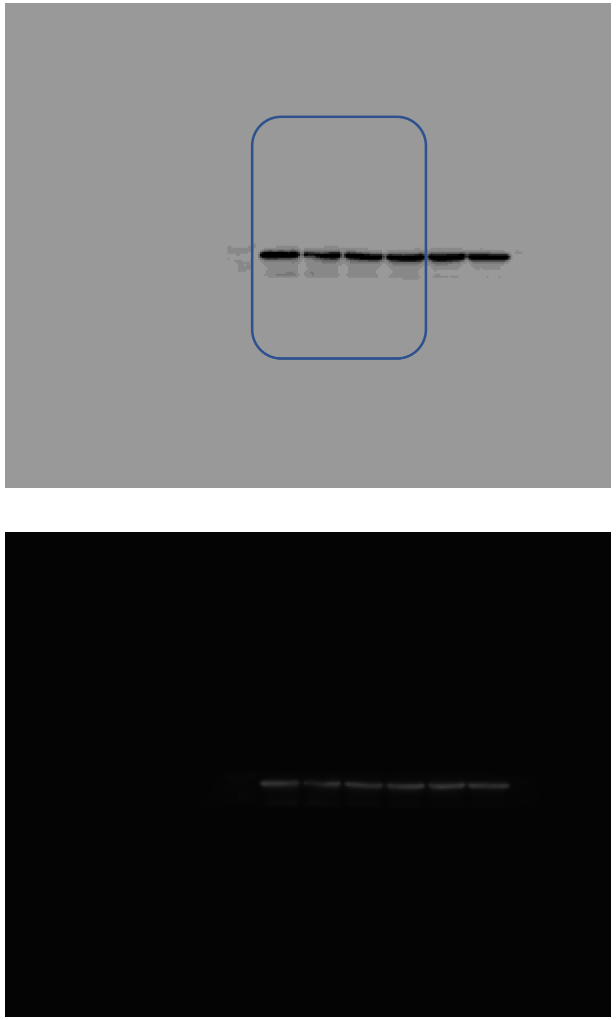

Figure 8 p-SMAD2/3

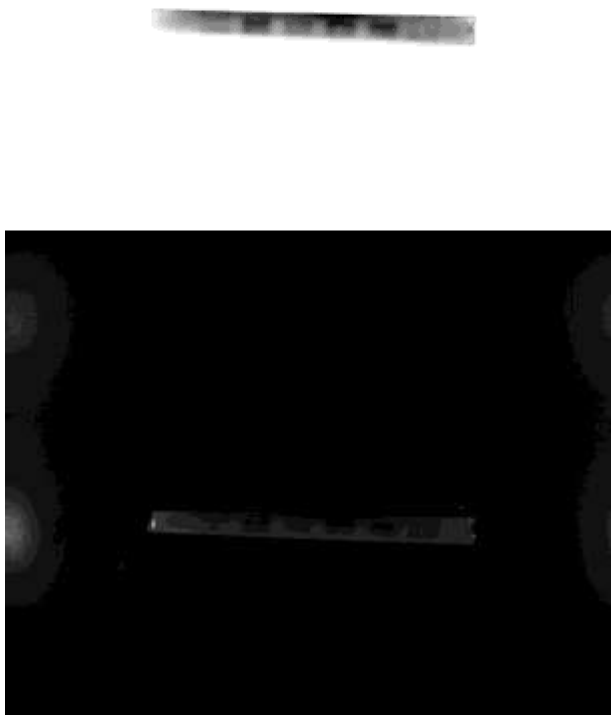

GADPH (36kda)

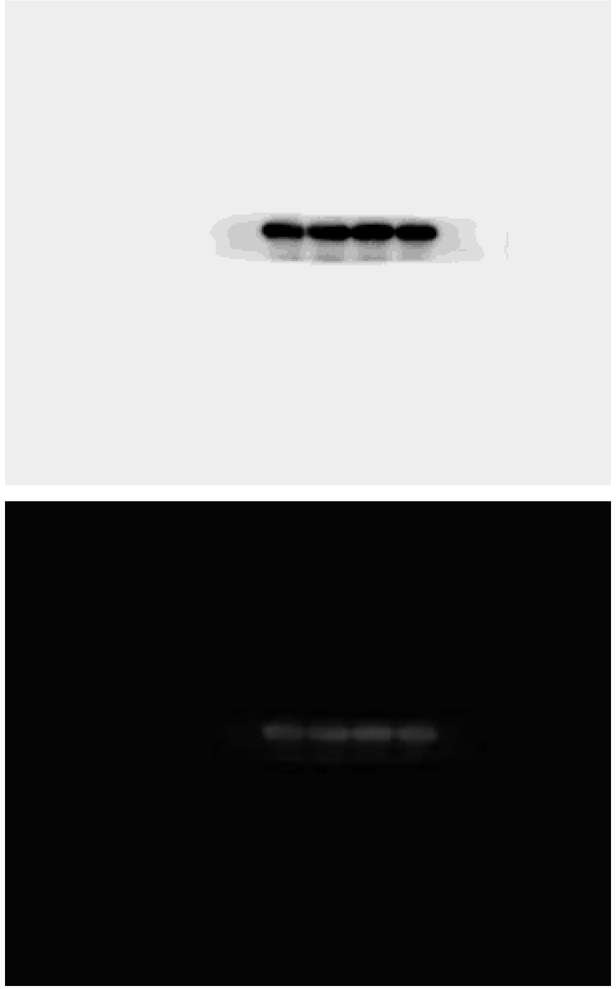

p-ERK(42/44kda)

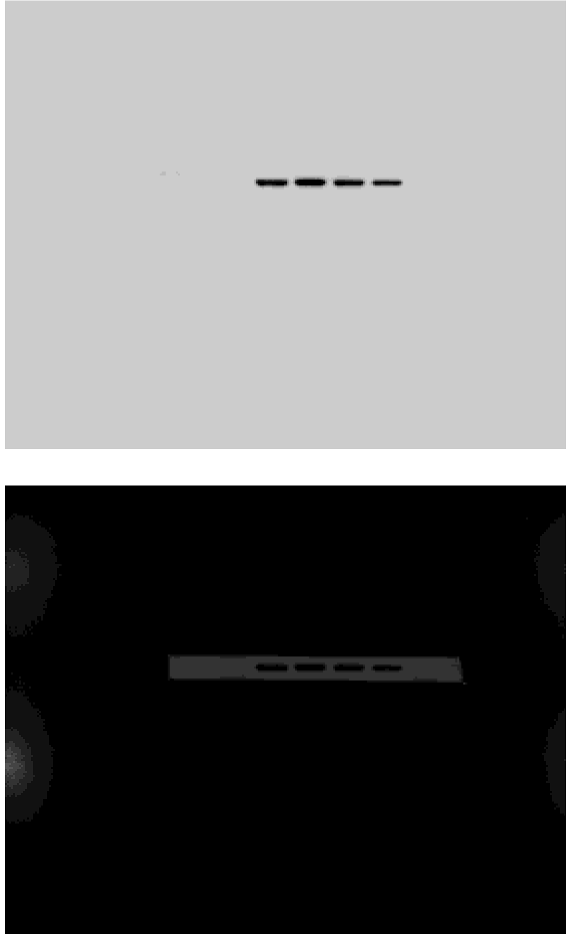

ERK(42/44kda)

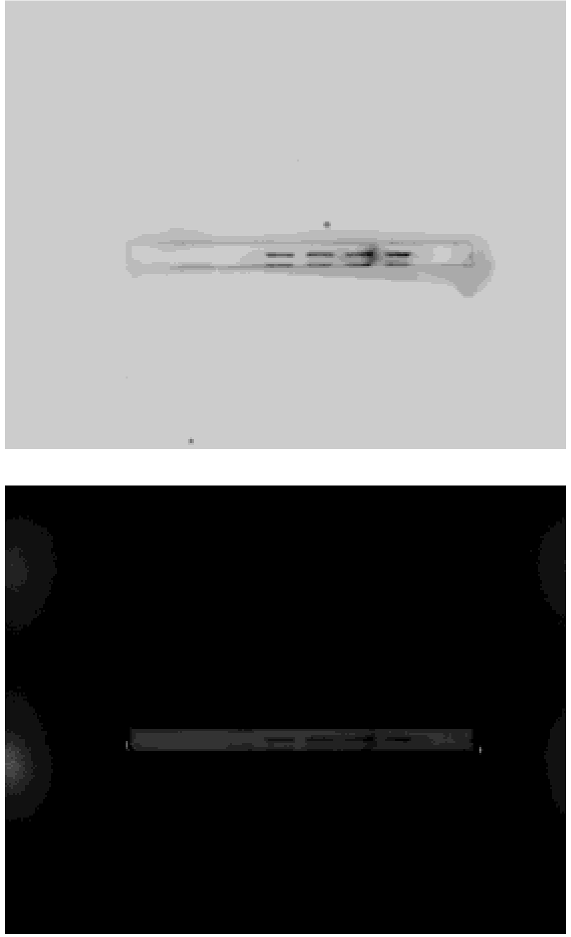

Supplement: Supplementary file 2 [file DataSheet2.pdf]
