# Supplementary figures and images for: GLP-1 and glucagon receptor dual agonism ameliorates kidney allograft fibrosis by improving lipid metabolism
Source: Front Immunol. 2025 Mar 31;16:1551136. doi: 10.3389/fimmu.2025.1551136 (PMC11994718; doi:10.3389/fimmu.2025.1551136)

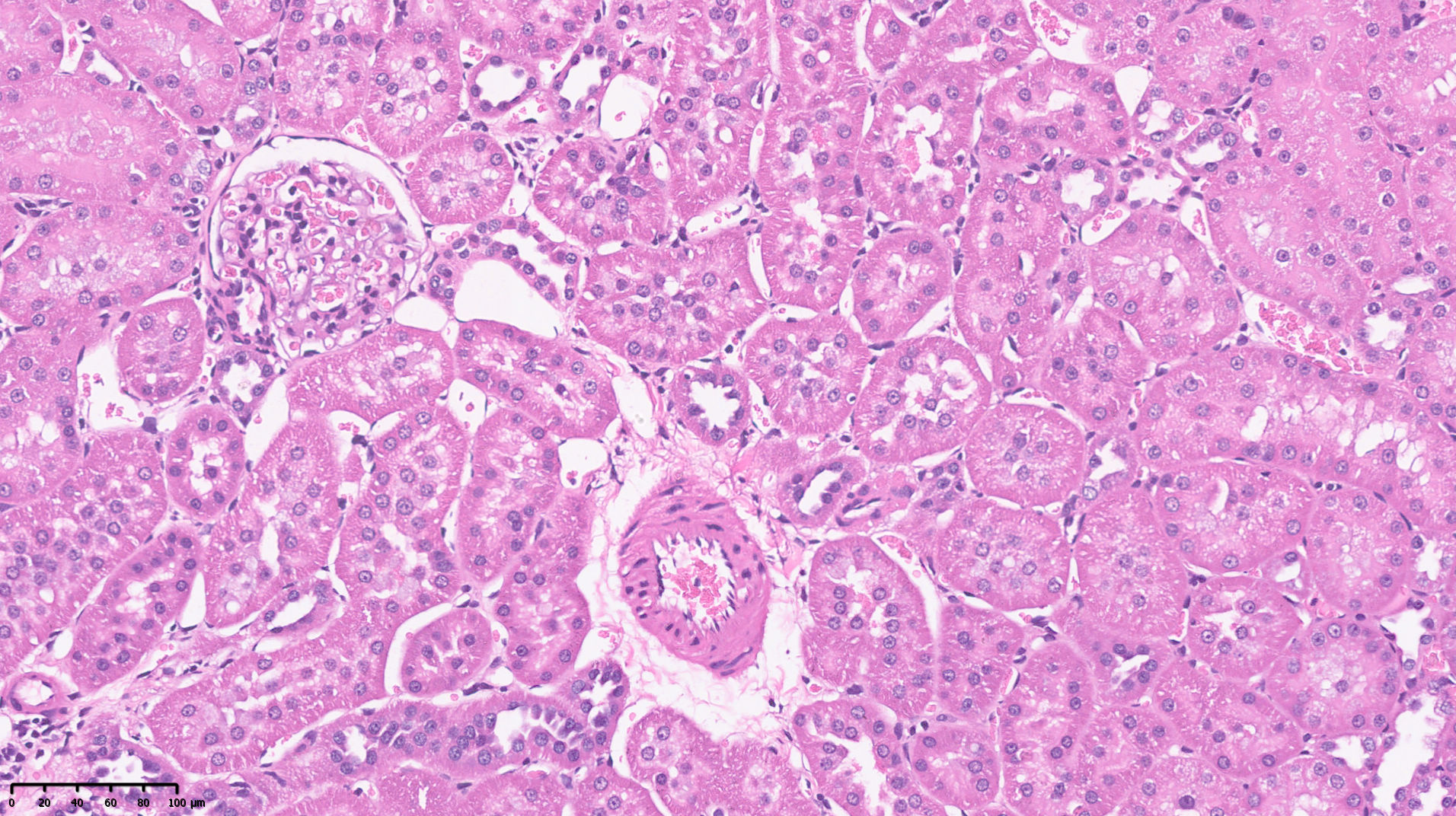

Supplement: Supplementary file 3 [file DataSheet3.zip › Routine staining picture/syngenic control_ HE.jpg]

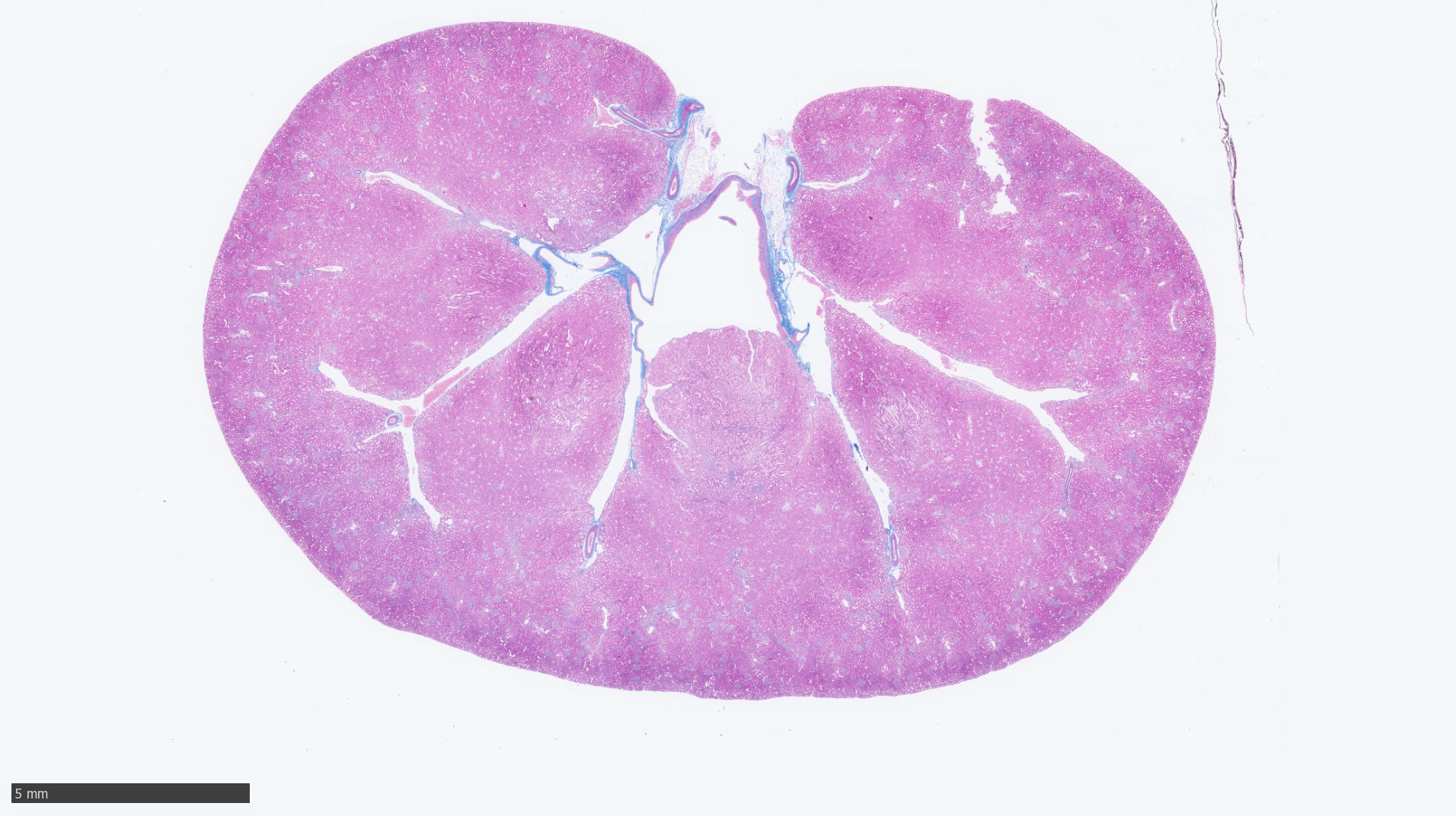

Supplement: Supplementary file 3 [file DataSheet3.zip › Routine staining picture/syngenic control_ MASSON.jpg]

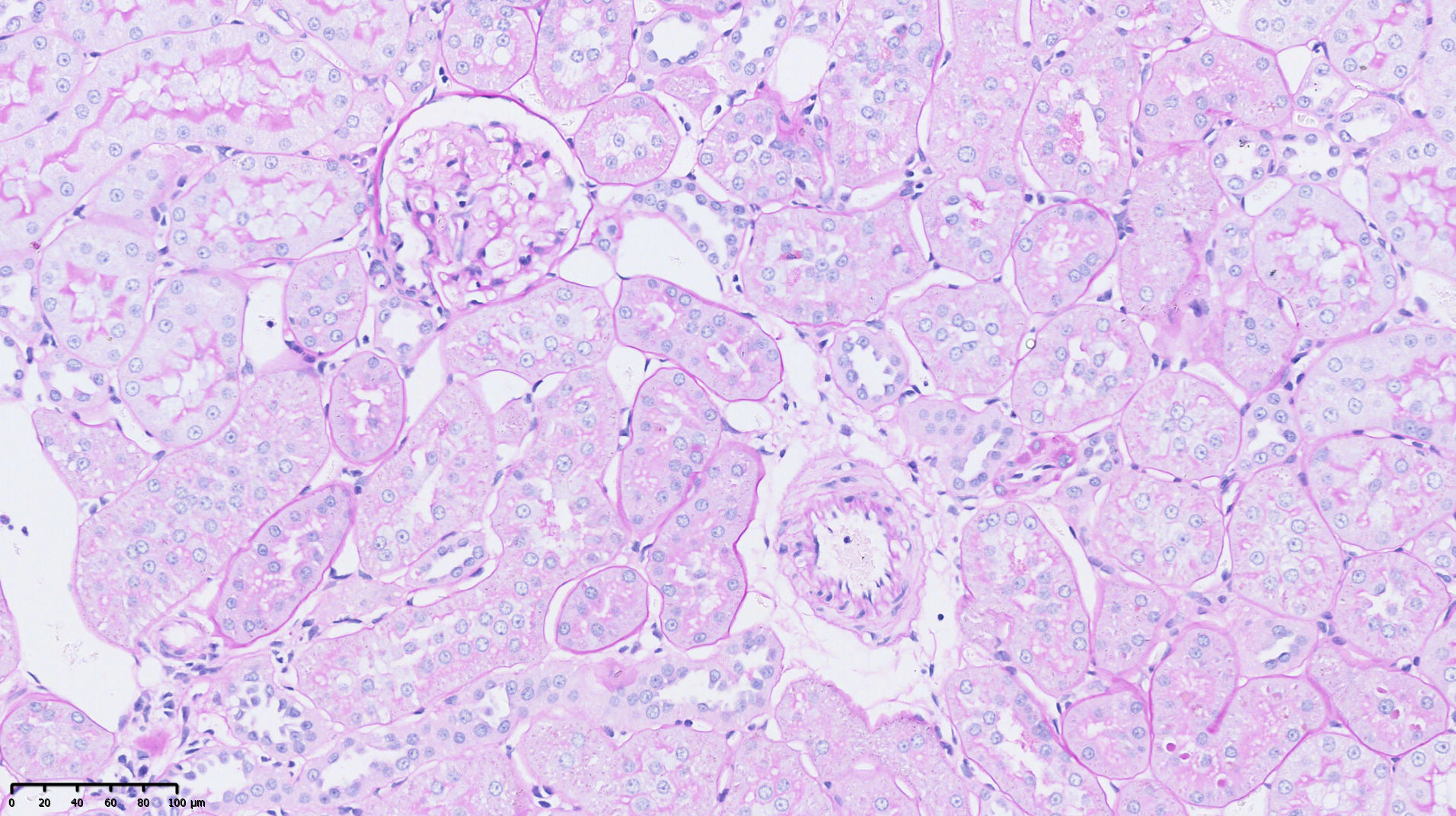

Supplement: Supplementary file 3 [file DataSheet3.zip › Routine staining picture/syngenic control_ PAS.jpg]

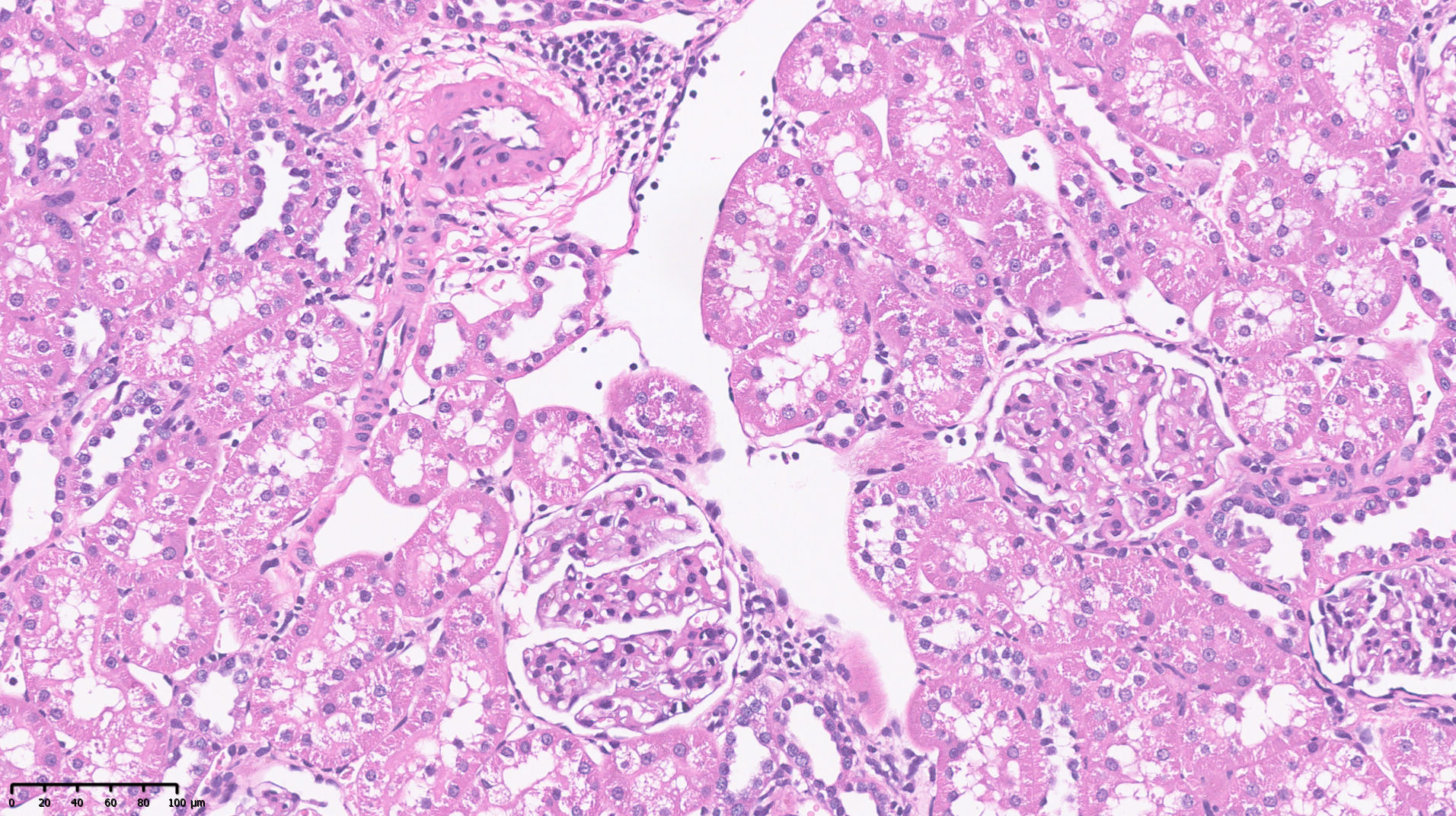

Supplement: Supplementary file 3 [file DataSheet3.zip › Routine staining picture/TB001 group_ HE.jpg]

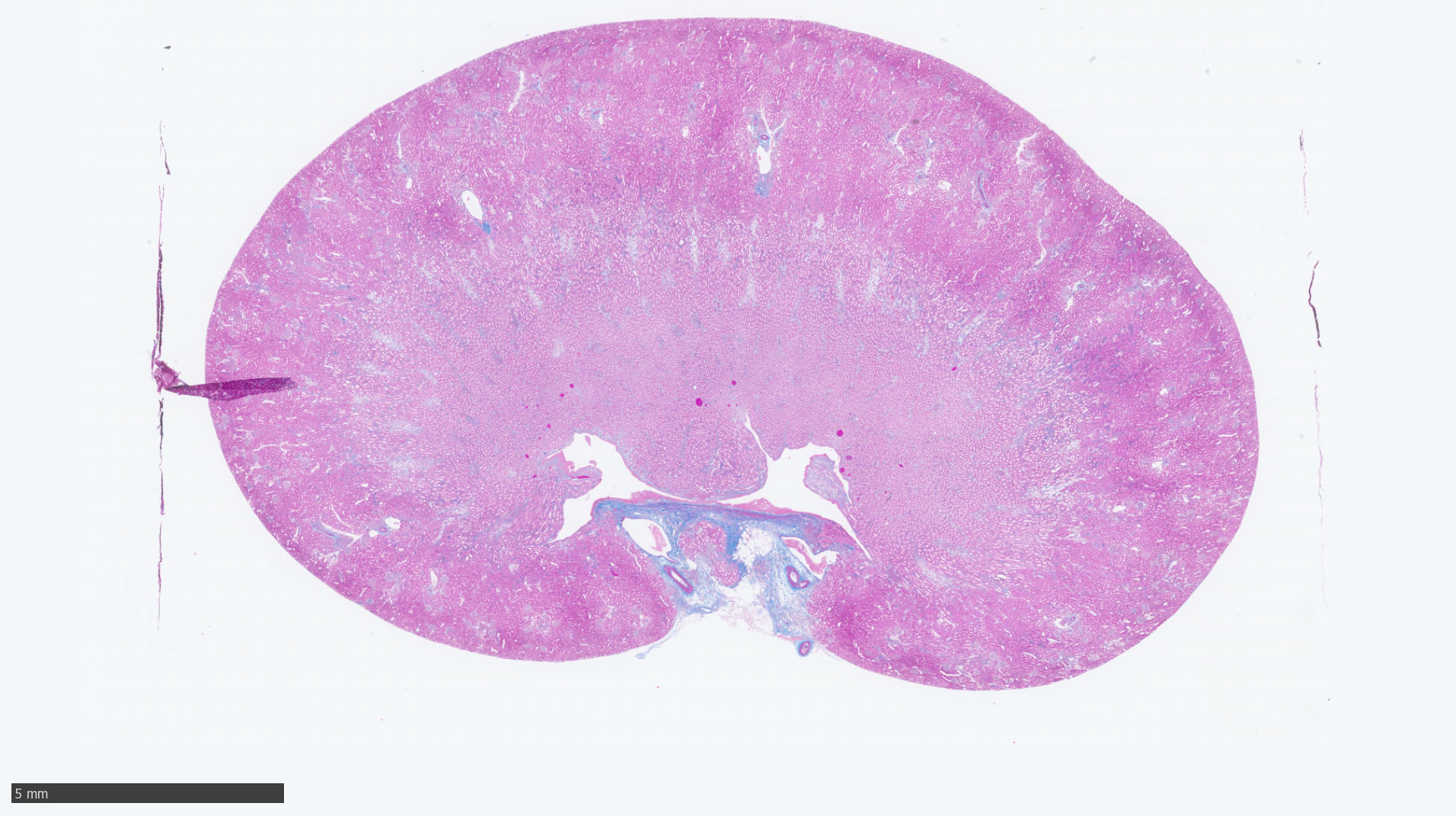

Supplement: Supplementary file 3 [file DataSheet3.zip › Routine staining picture/TB001 group_ MASSON.jpg]

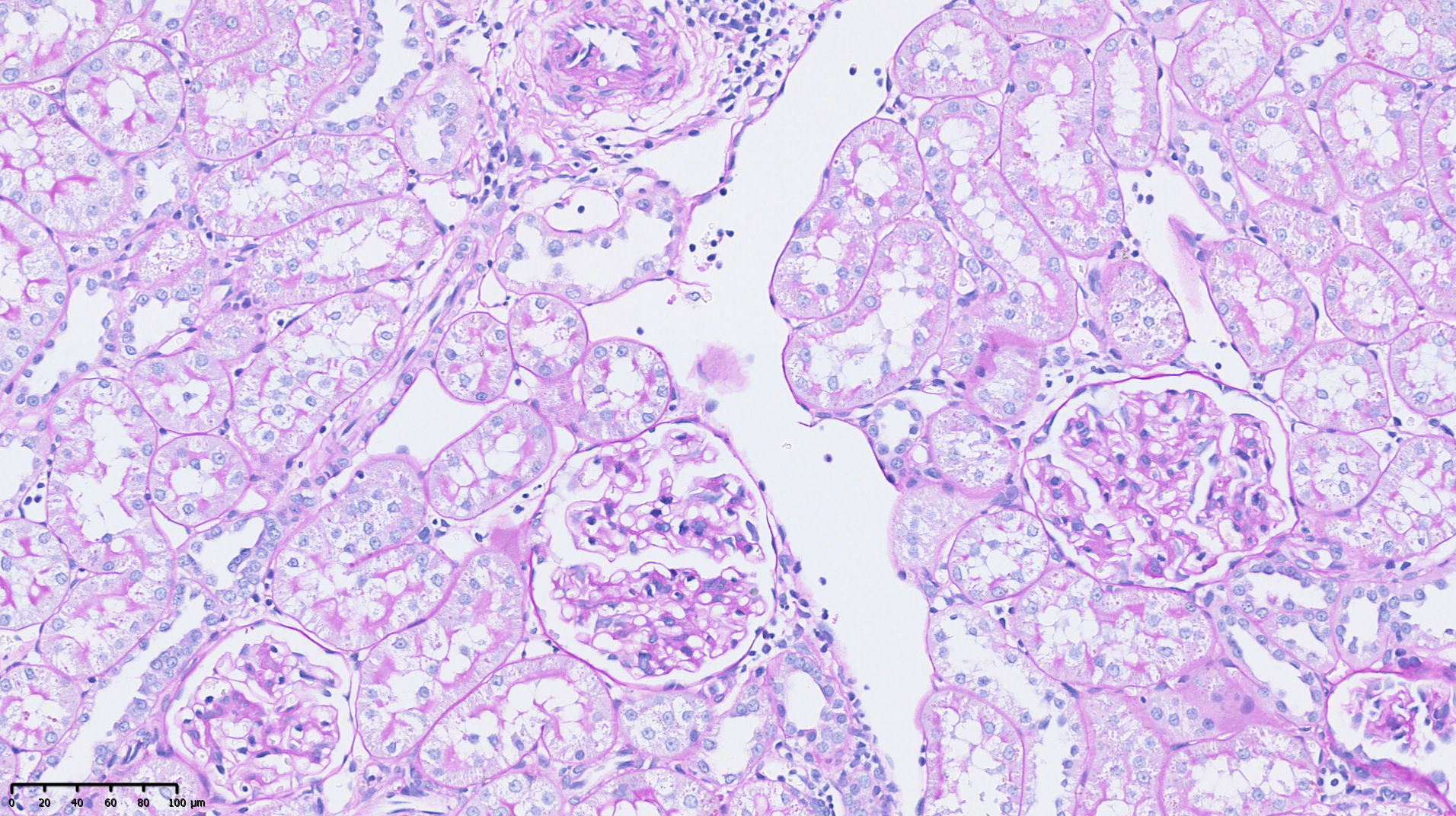

Supplement: Supplementary file 3 [file DataSheet3.zip › Routine staining picture/TB001 group_ PAS.jpg]

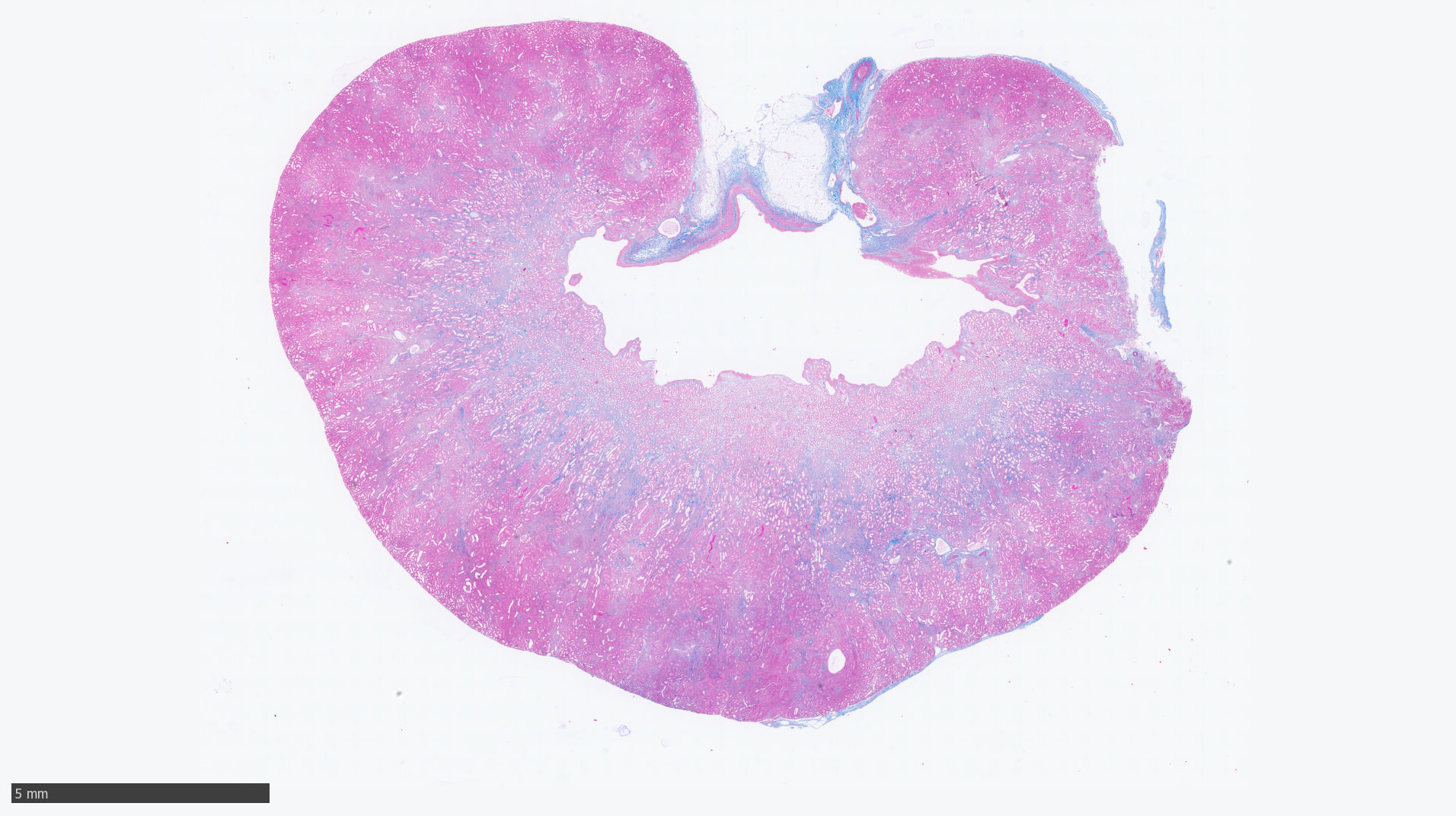

Supplement: Supplementary file 3 [file DataSheet3.zip › Routine staining picture/Vehicle group- MASSON_.jpg]

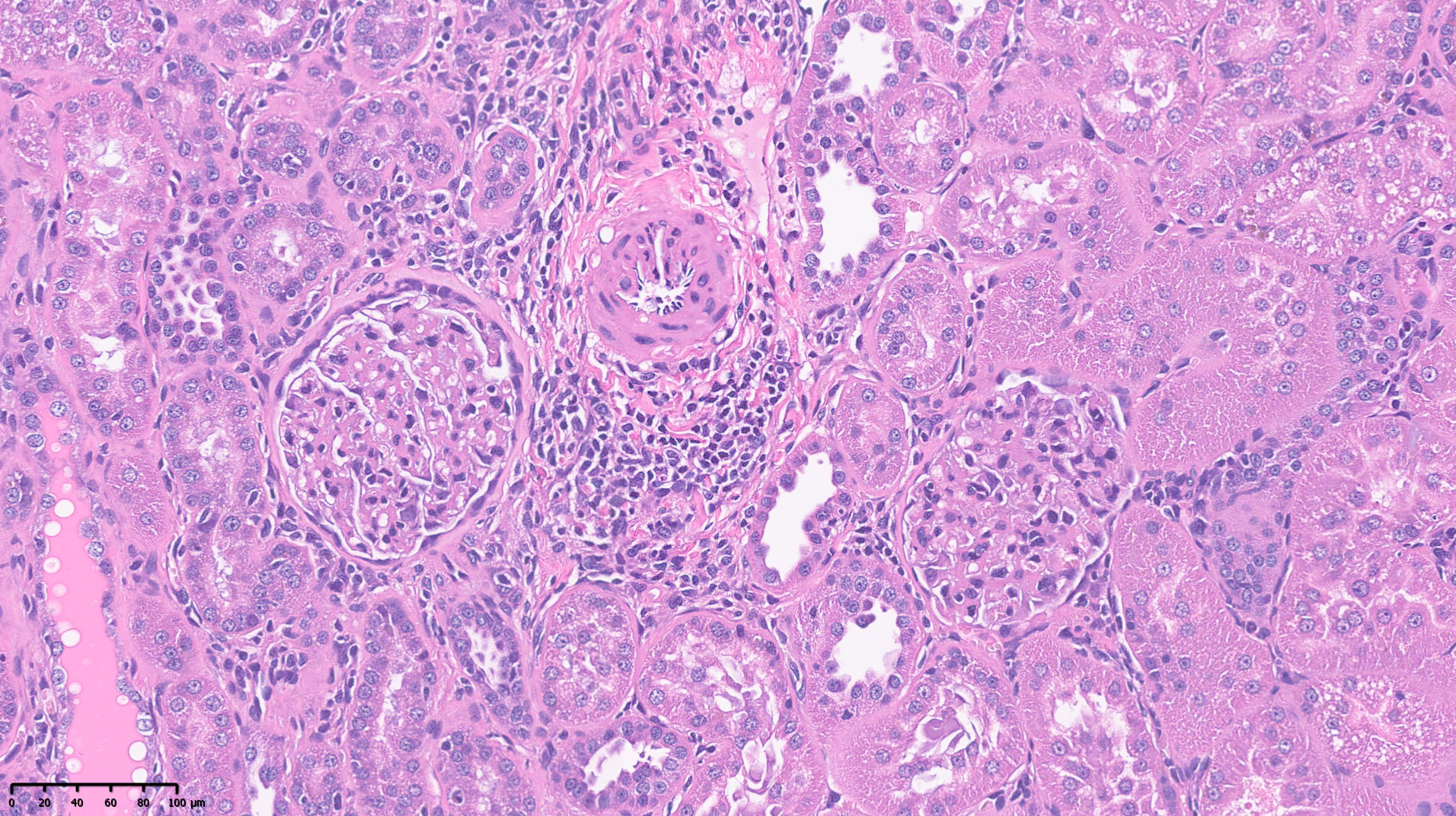

Supplement: Supplementary file 3 [file DataSheet3.zip › Routine staining picture/Vehicle group_ HE.jpg]

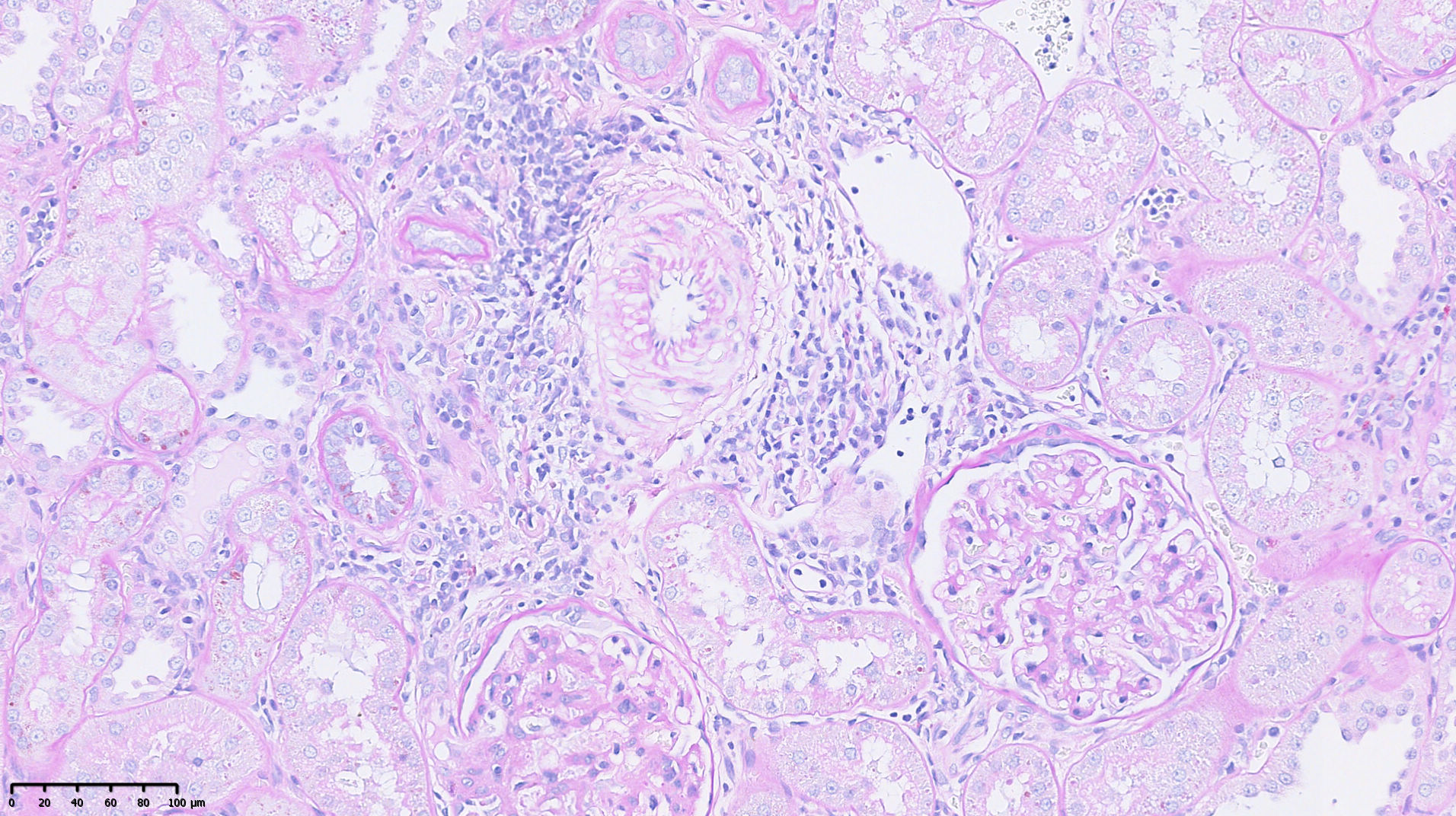

Supplement: Supplementary file 3 [file DataSheet3.zip › Routine staining picture/Vehicle group_ PAS.jpg]

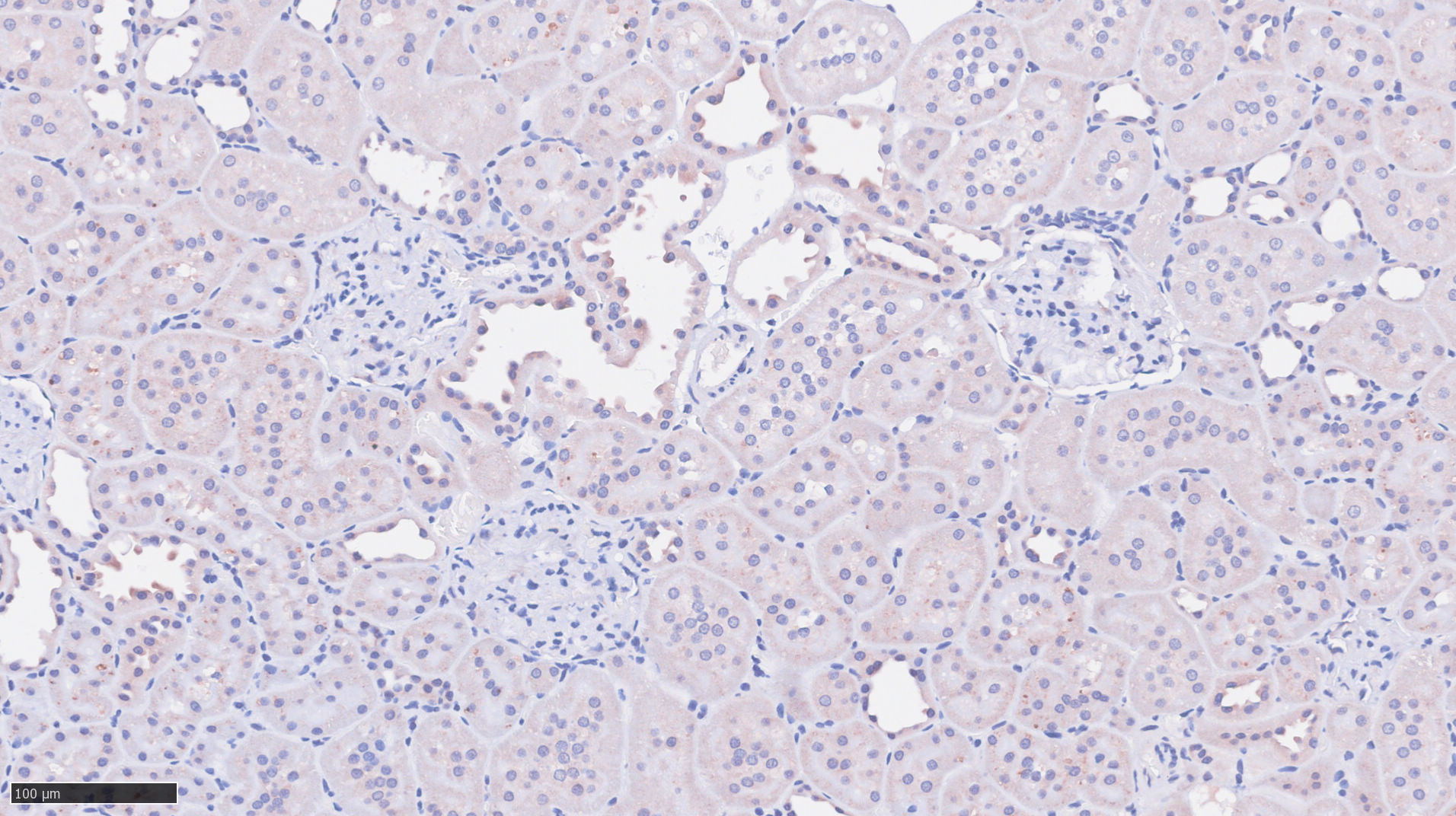

Supplement: Supplementary file 3 [file DataSheet3.zip › IHC staining picture/CCL2/syngenic control.jpg]

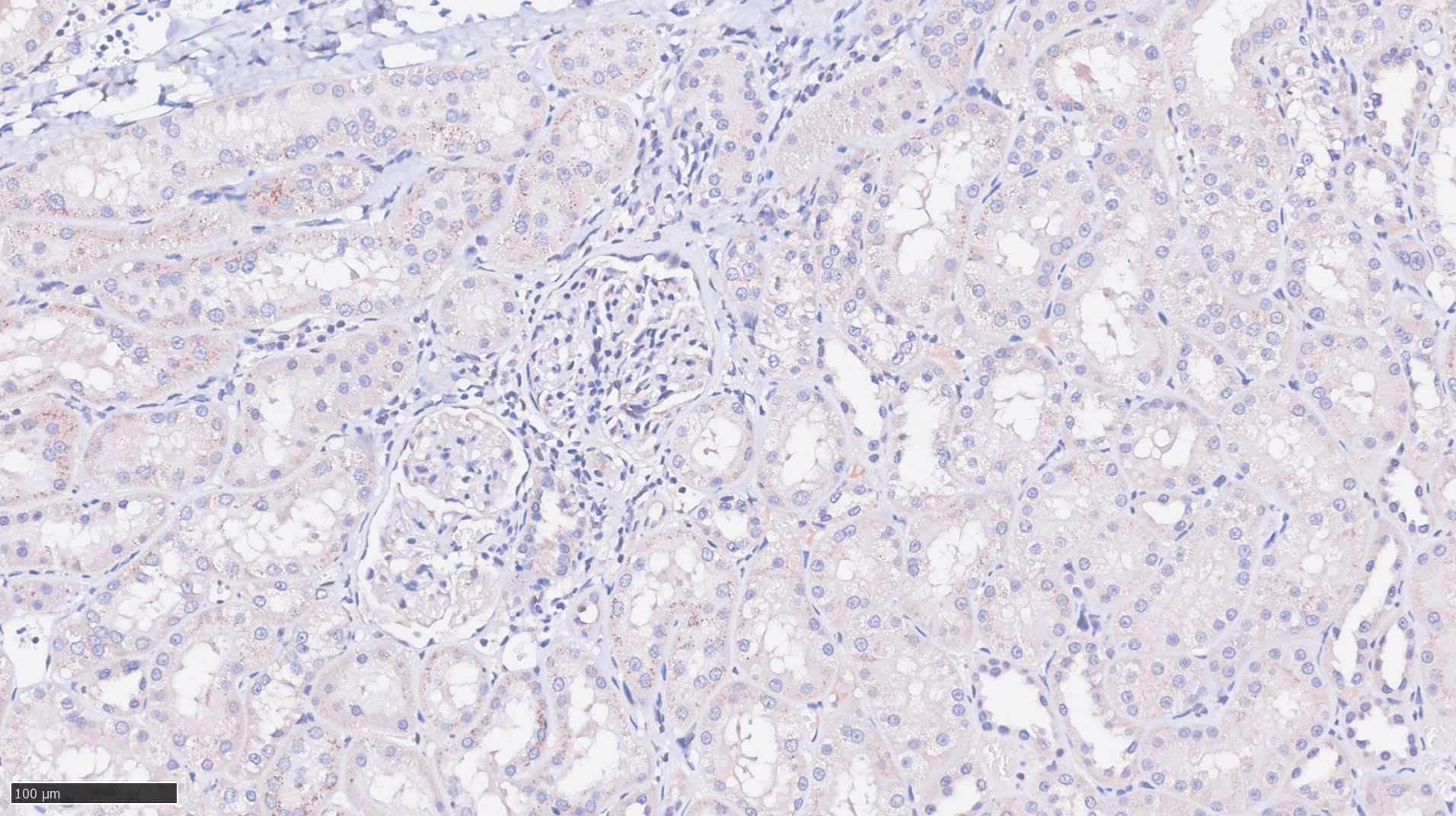

Supplement: Supplementary file 3 [file DataSheet3.zip › IHC staining picture/CCL2/TB001 group.jpg]

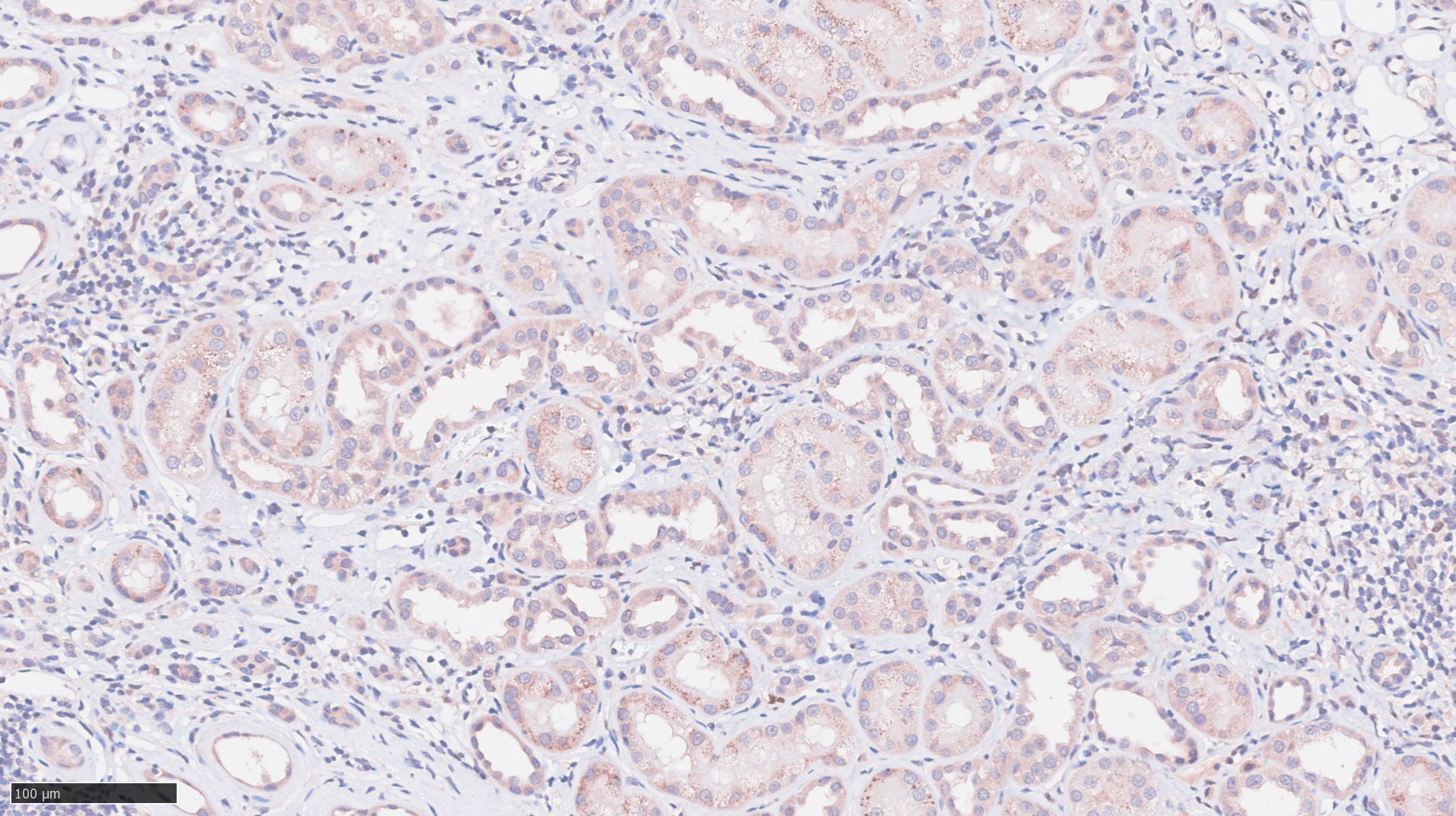

Supplement: Supplementary file 3 [file DataSheet3.zip › IHC staining picture/CCL2/Vehicle group.jpg]

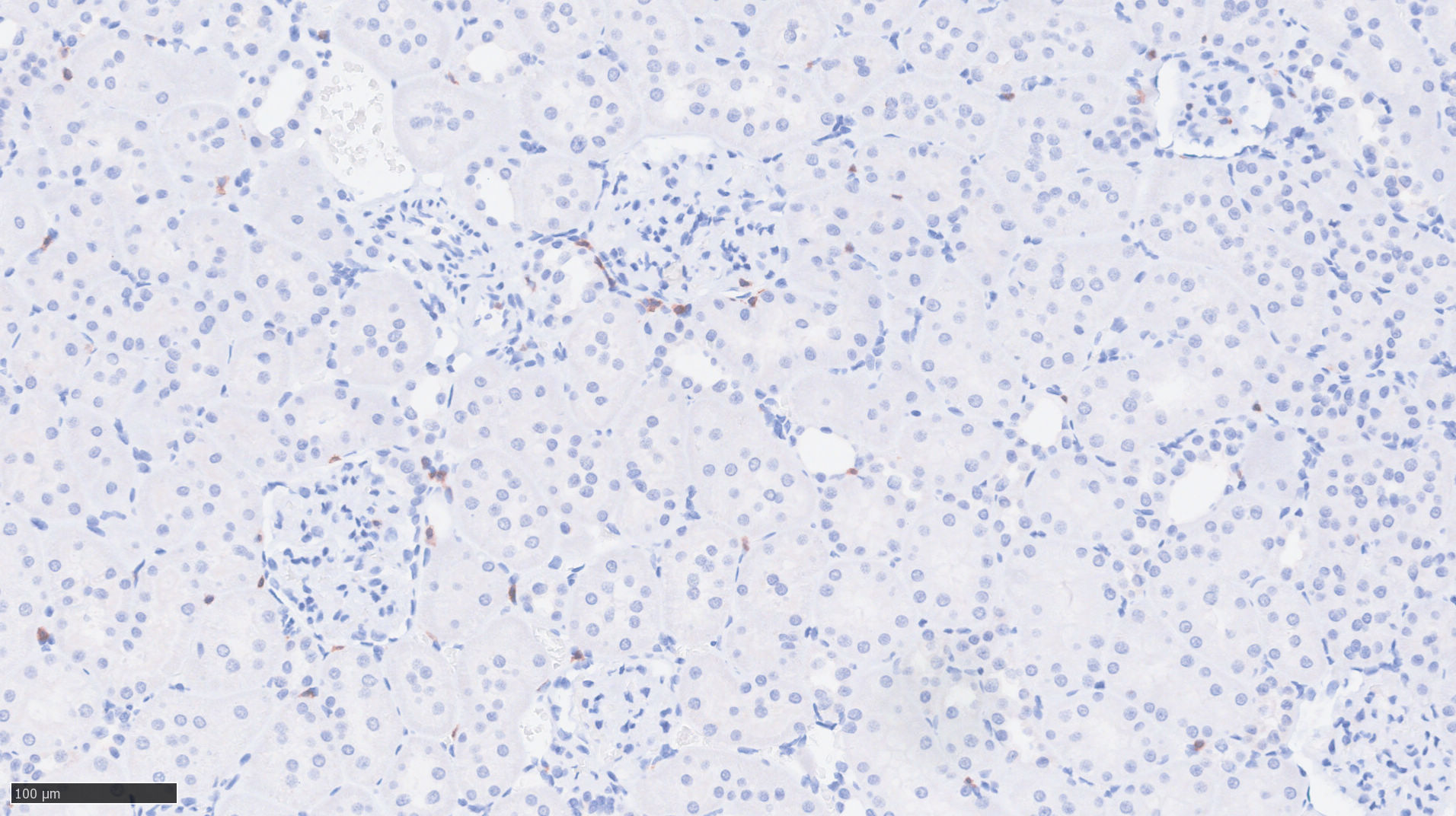

Supplement: Supplementary file 3 [file DataSheet3.zip › IHC staining picture/CD3/syngenic control.jpg]

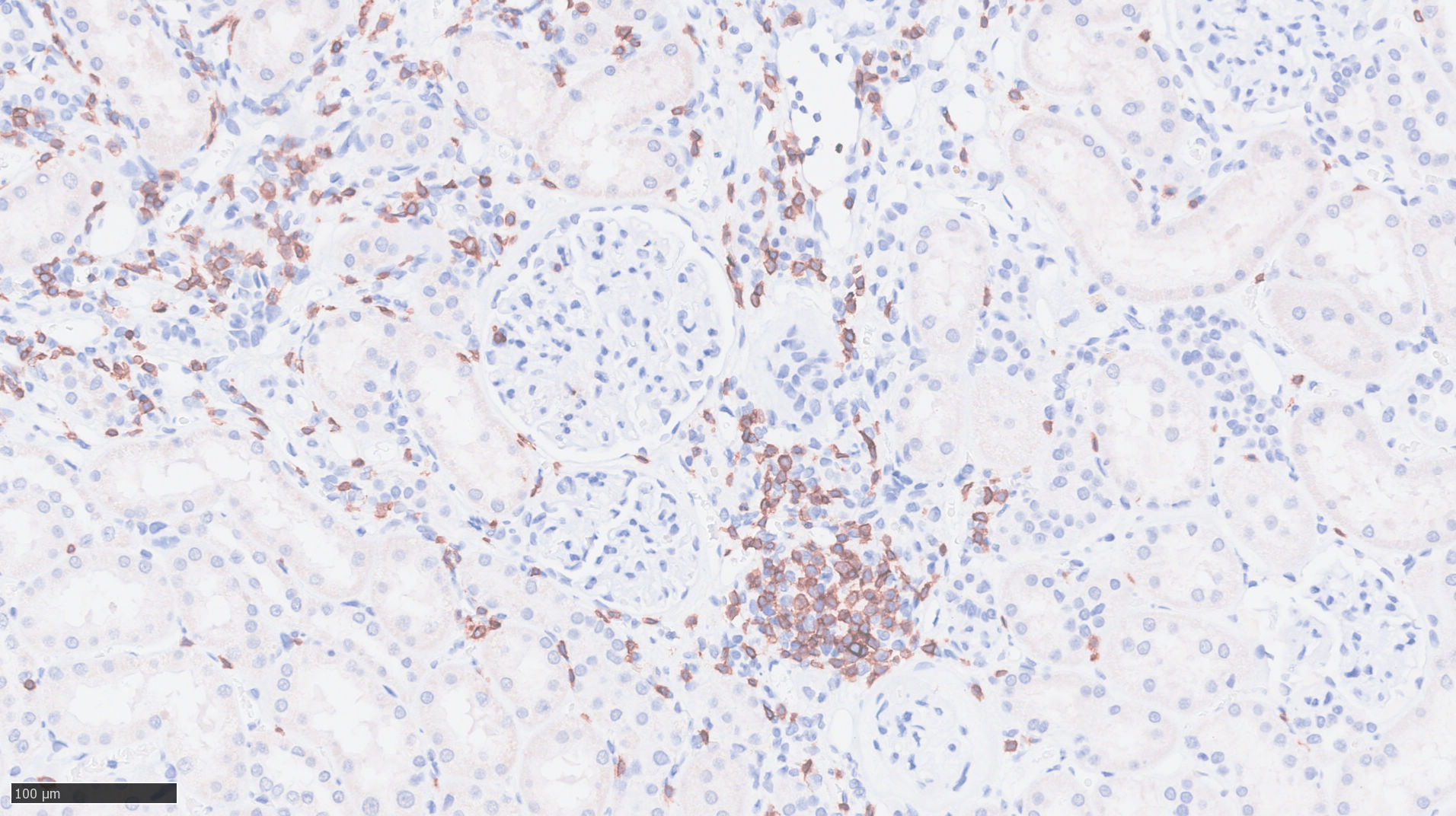

Supplement: Supplementary file 3 [file DataSheet3.zip › IHC staining picture/CD3/TB001 group.jpg]

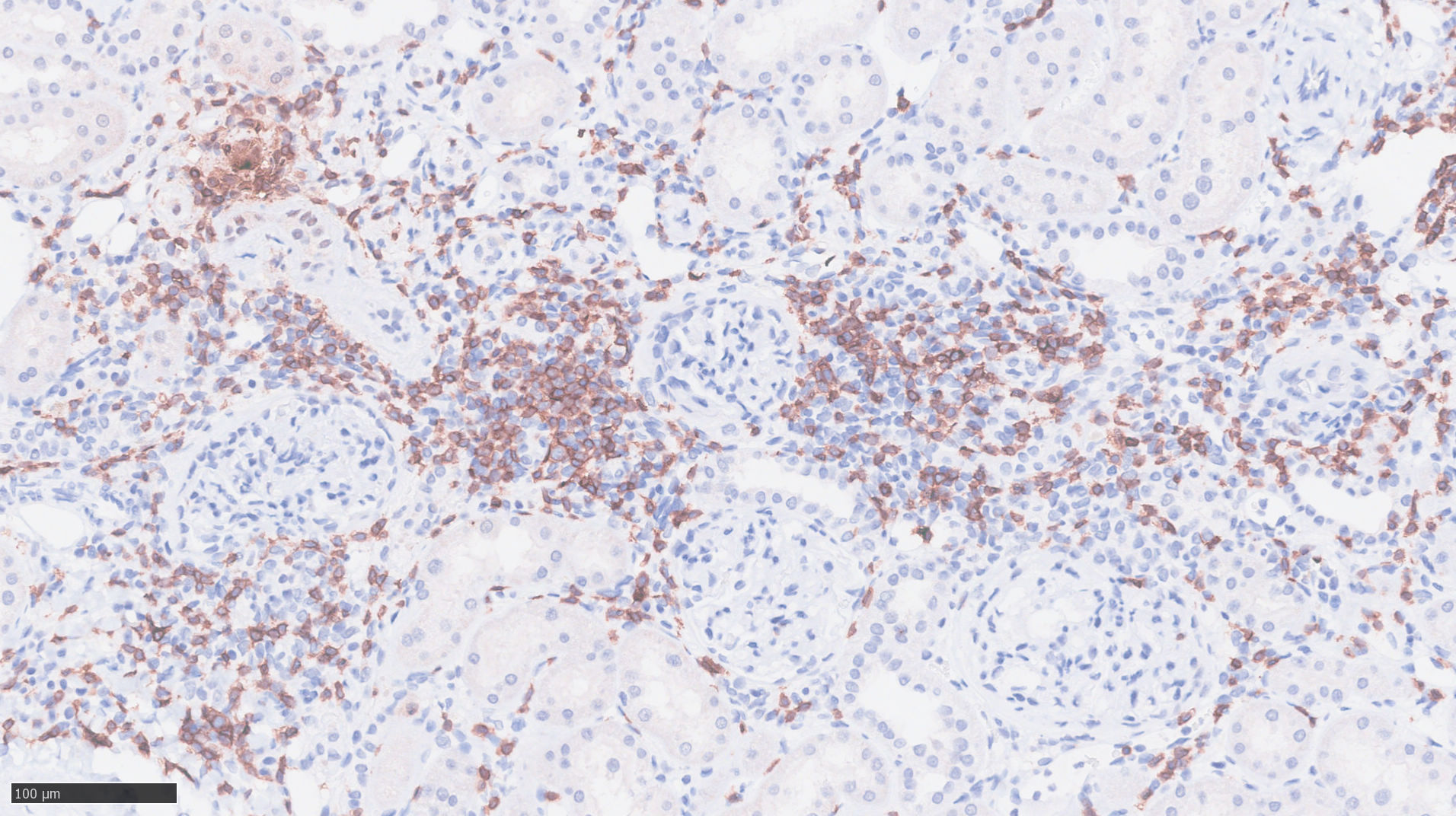

Supplement: Supplementary file 3 [file DataSheet3.zip › IHC staining picture/CD3/vehicle group.jpg]

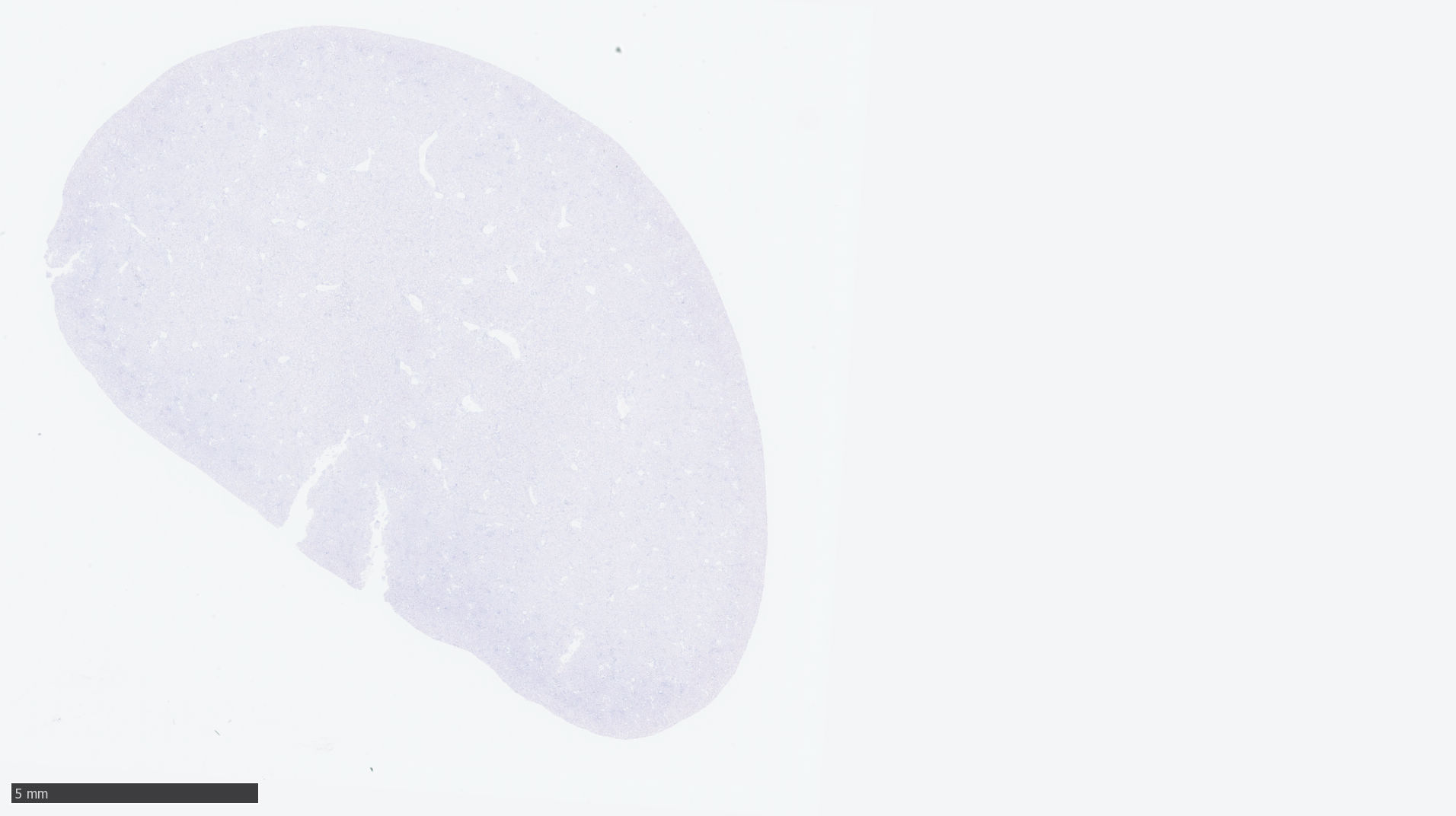

Supplement: Supplementary file 3 [file DataSheet3.zip › IHC staining picture/CD68/syngenic control.jpg]

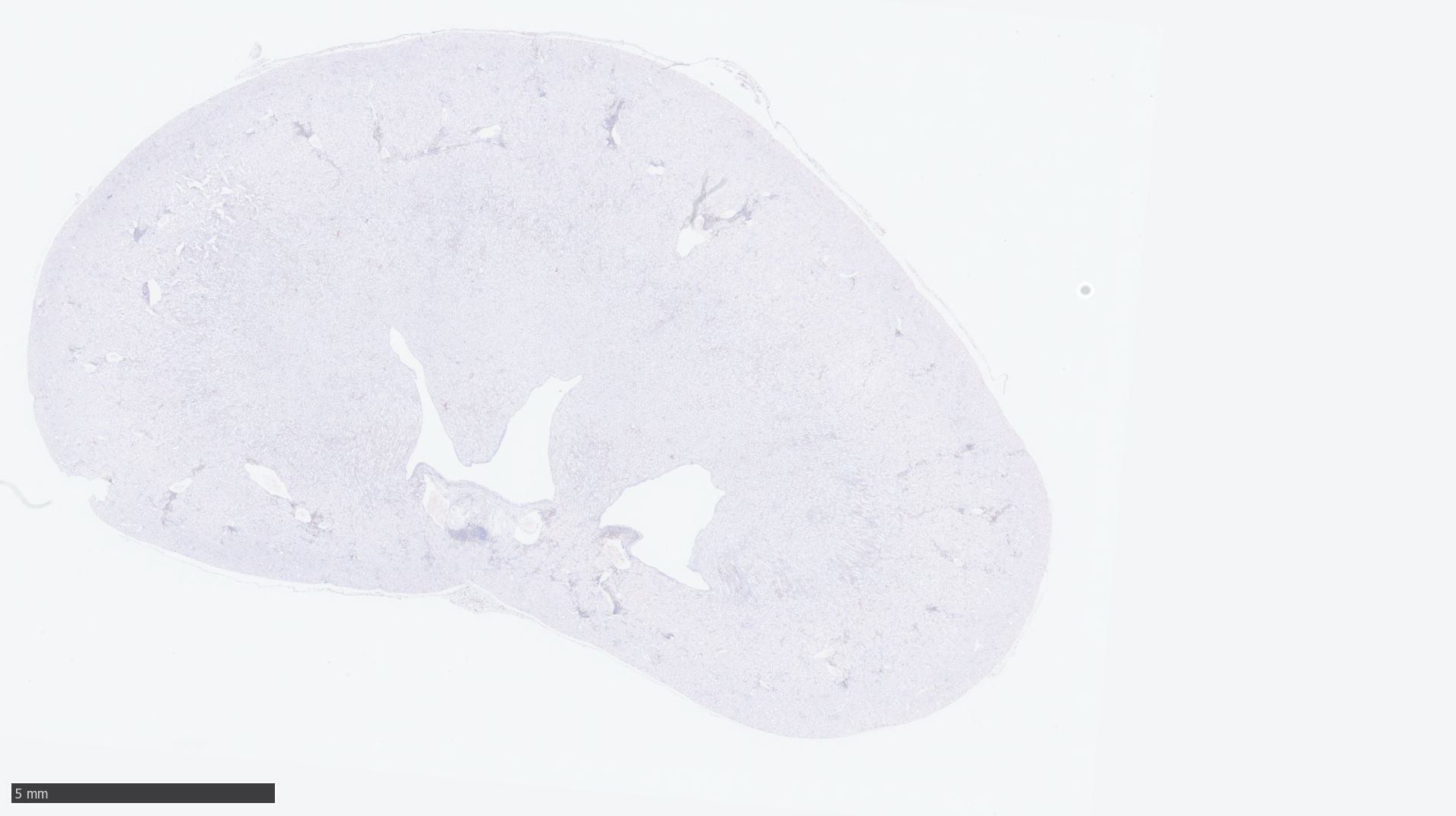

Supplement: Supplementary file 3 [file DataSheet3.zip › IHC staining picture/CD68/TB001 group.jpg]

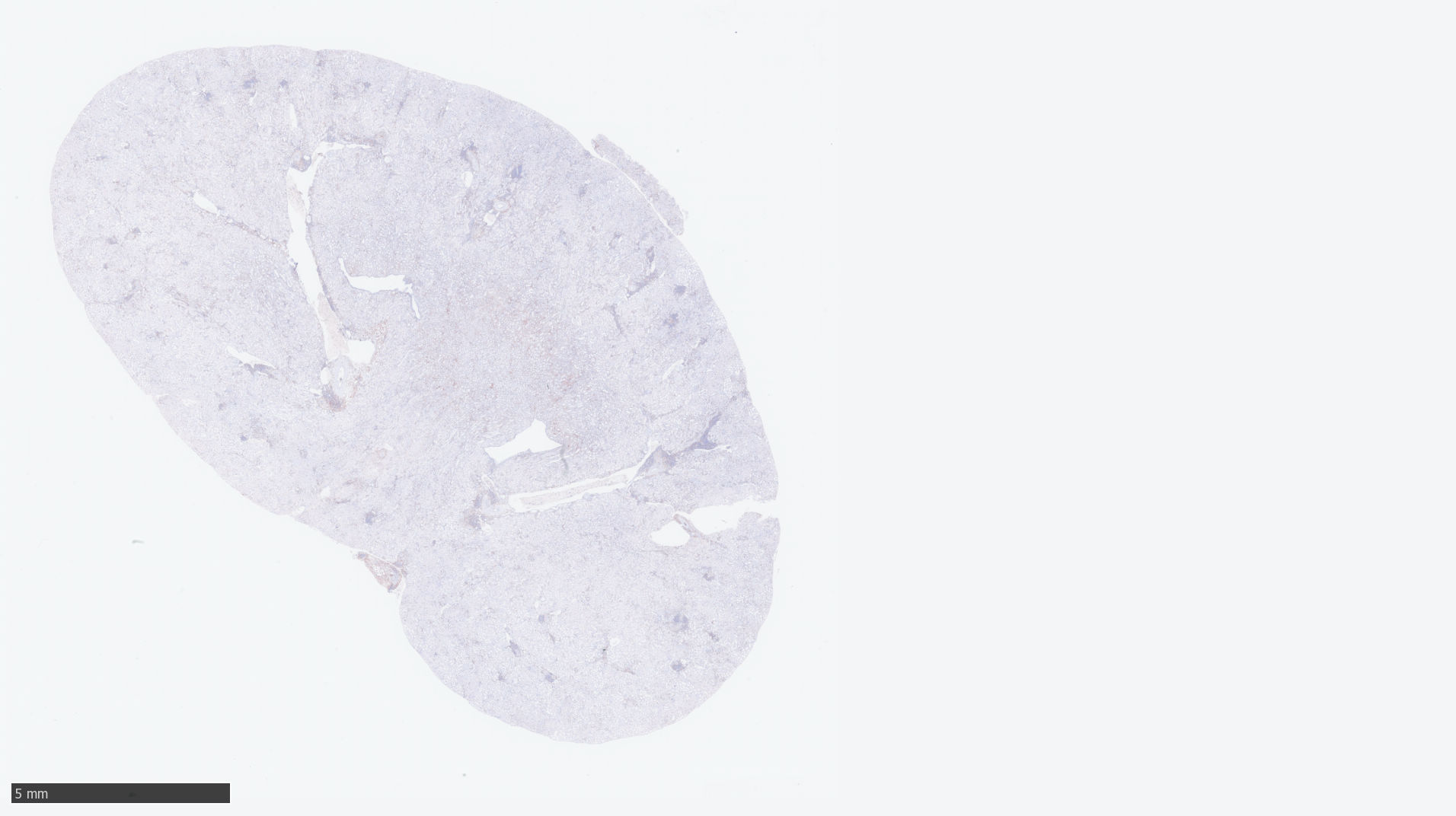

Supplement: Supplementary file 3 [file DataSheet3.zip › IHC staining picture/CD68/Vehicle group.jpg]

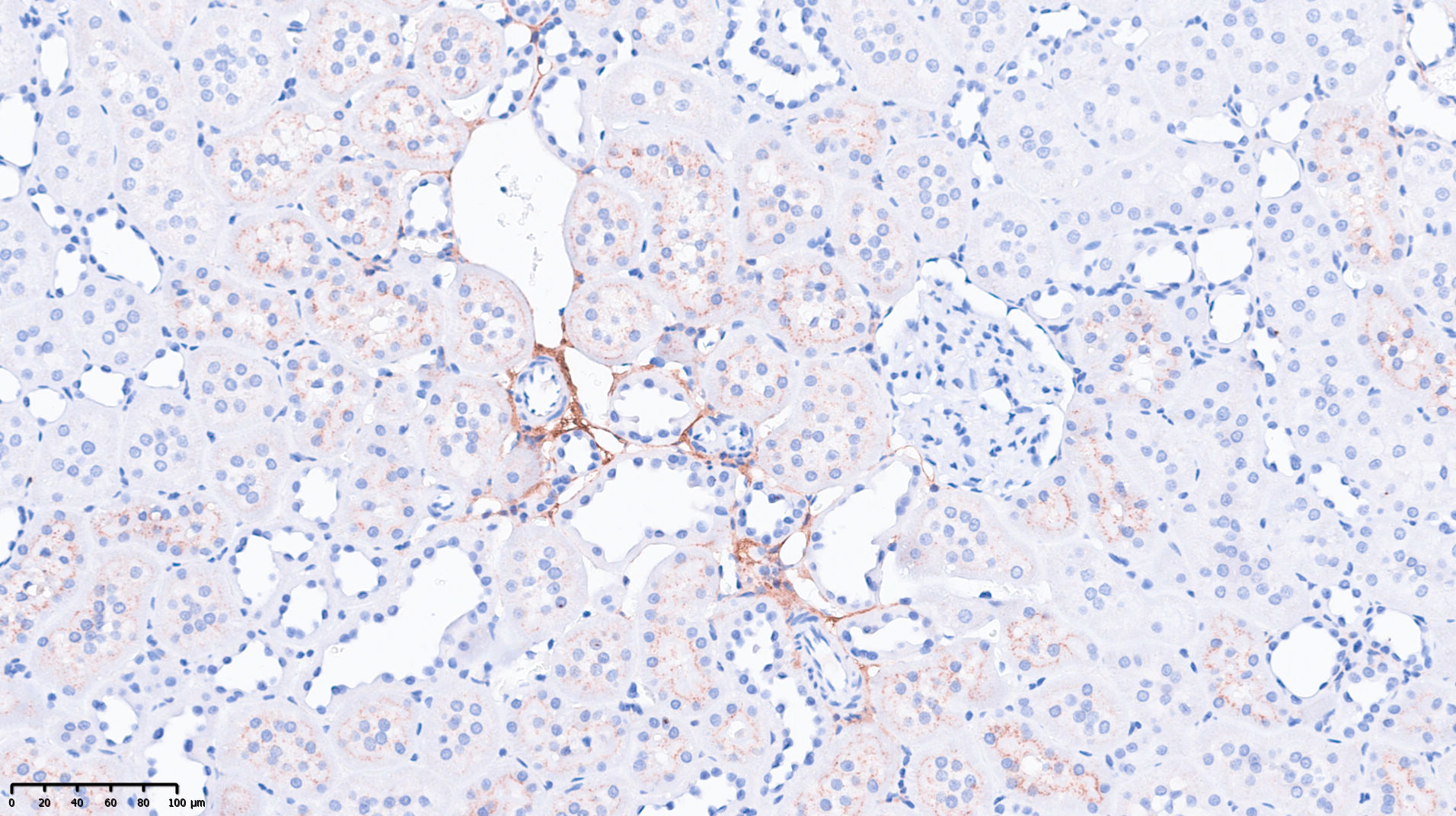

Supplement: Supplementary file 3 [file DataSheet3.zip › IHC staining picture/cola1/syngenic control.jpg]

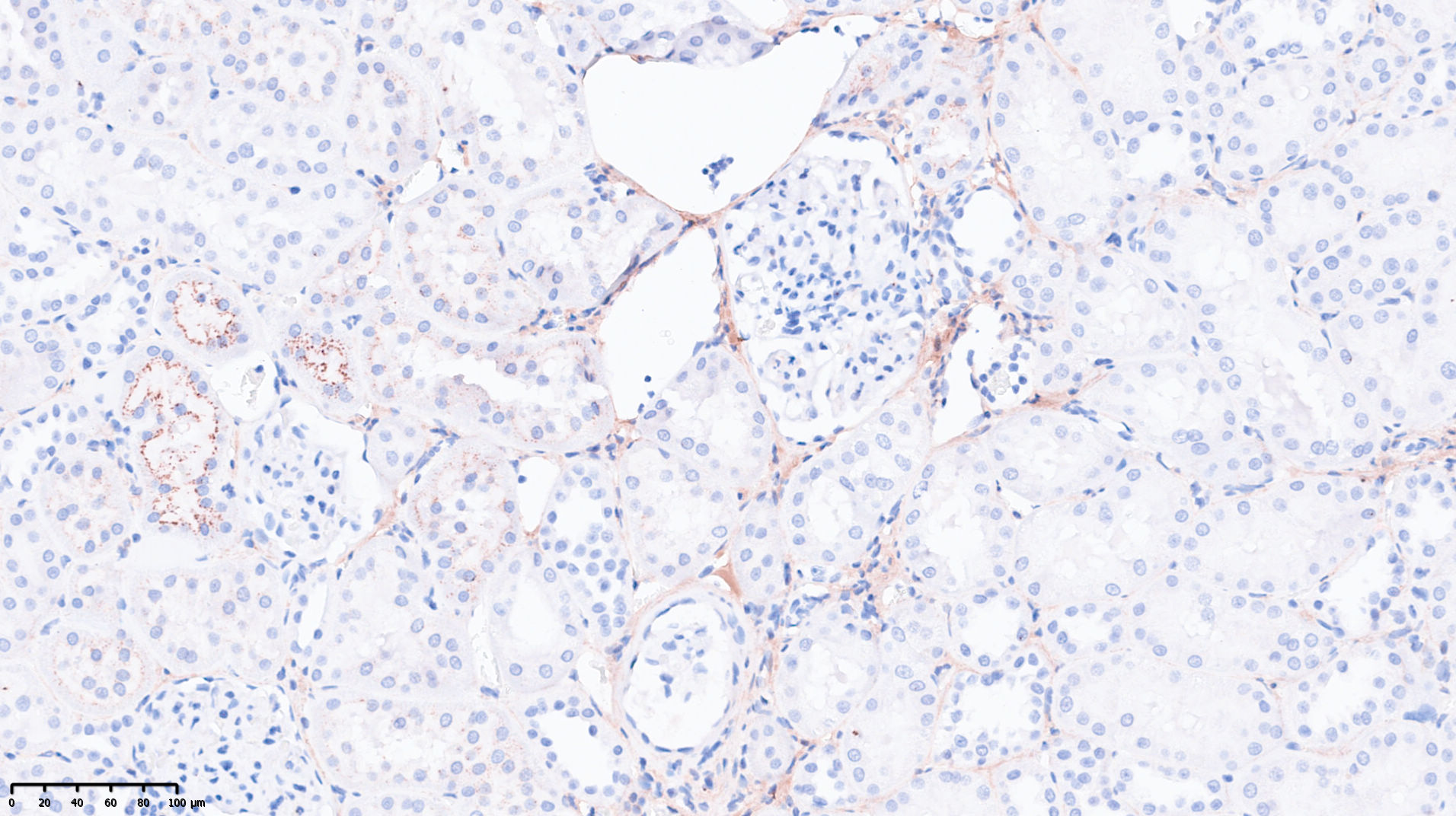

Supplement: Supplementary file 3 [file DataSheet3.zip › IHC staining picture/cola1/TB001 group.jpg]

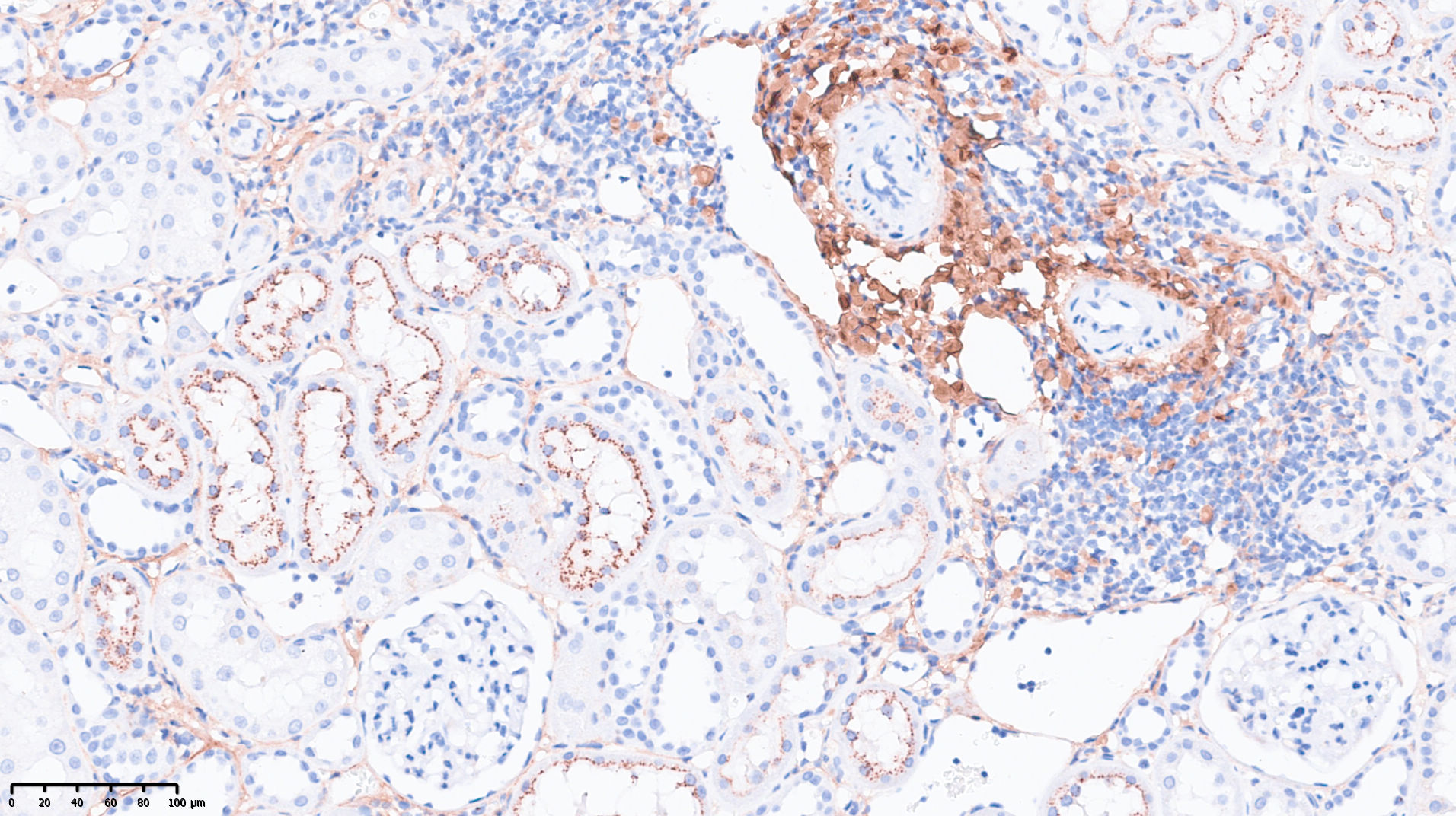

Supplement: Supplementary file 3 [file DataSheet3.zip › IHC staining picture/cola1/Vehicle group.jpg]

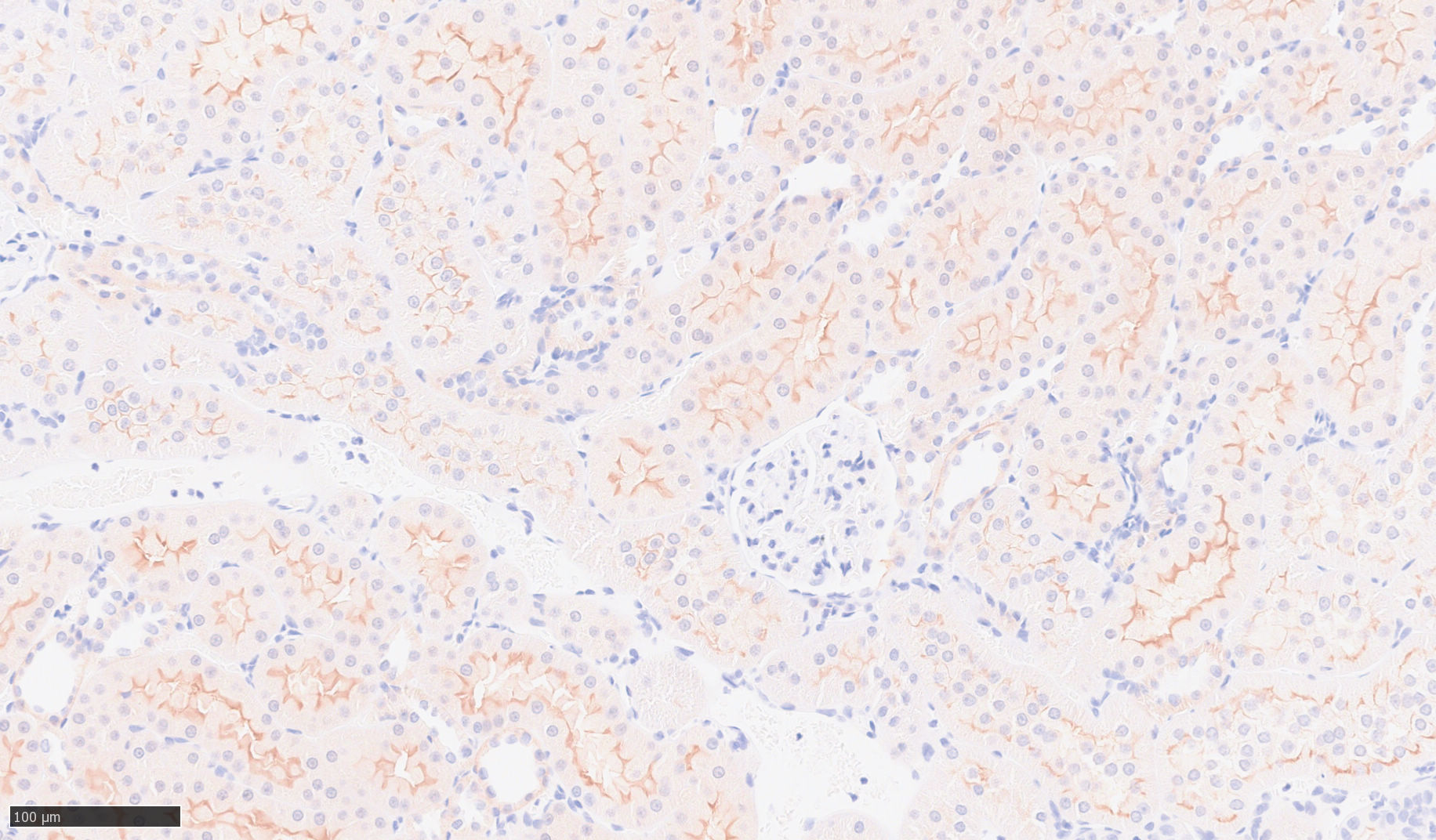

Supplement: Supplementary file 3 [file DataSheet3.zip › IHC staining picture/Ecadherin/syngenic control.jpg]

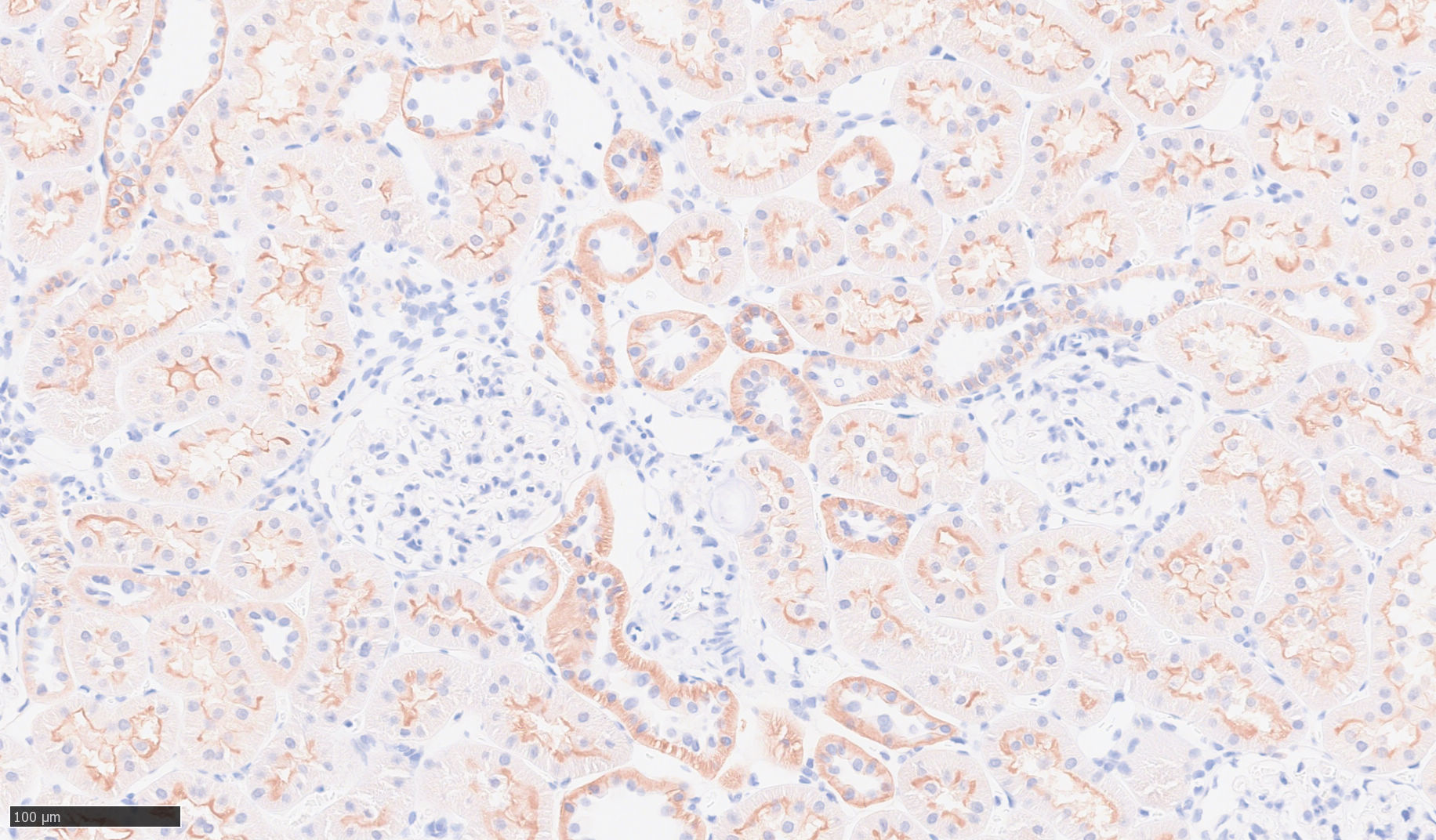

Supplement: Supplementary file 3 [file DataSheet3.zip › IHC staining picture/Ecadherin/TB001 group.jpg]

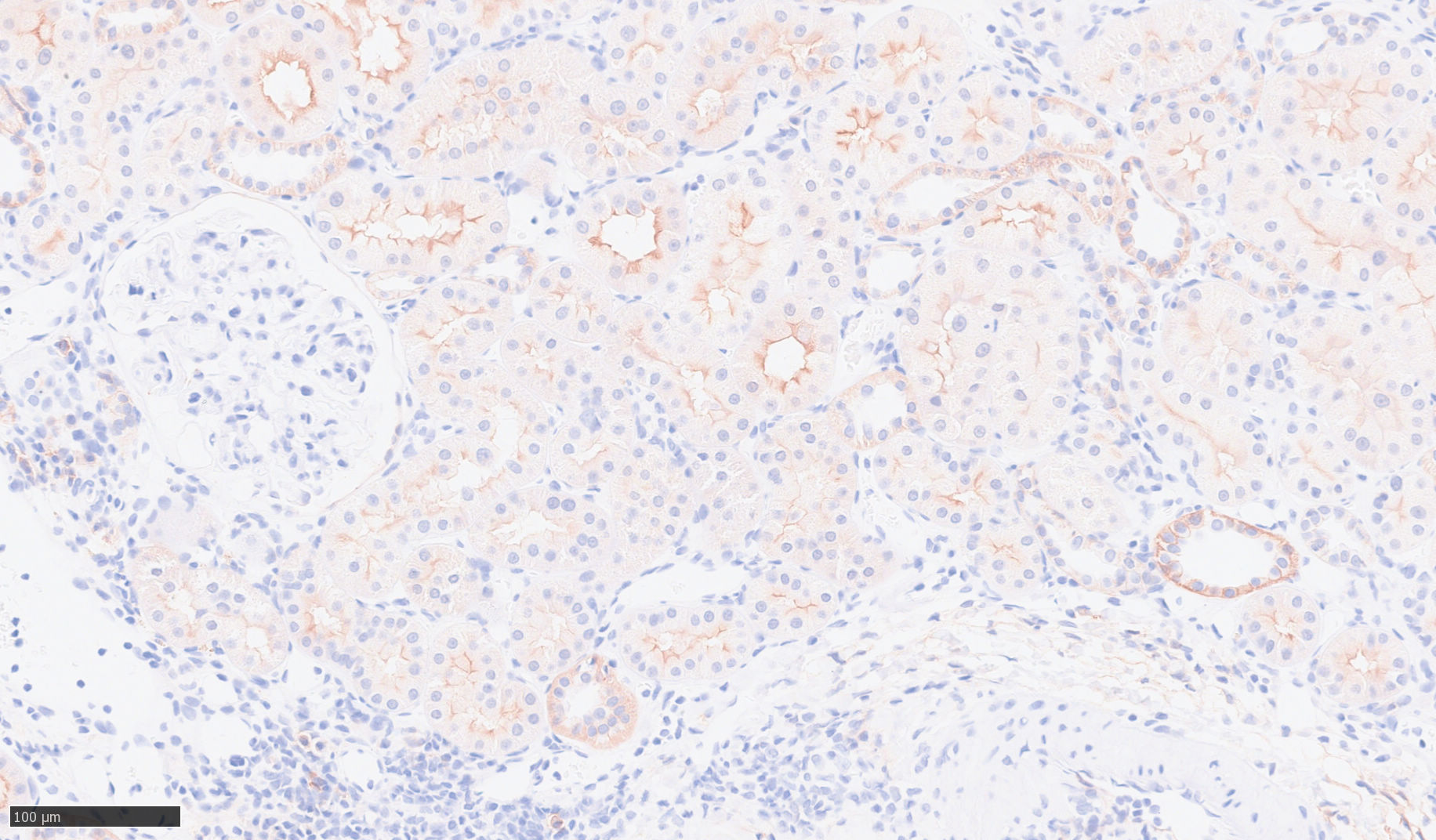

Supplement: Supplementary file 3 [file DataSheet3.zip › IHC staining picture/Ecadherin/Vehicle group.jpg]

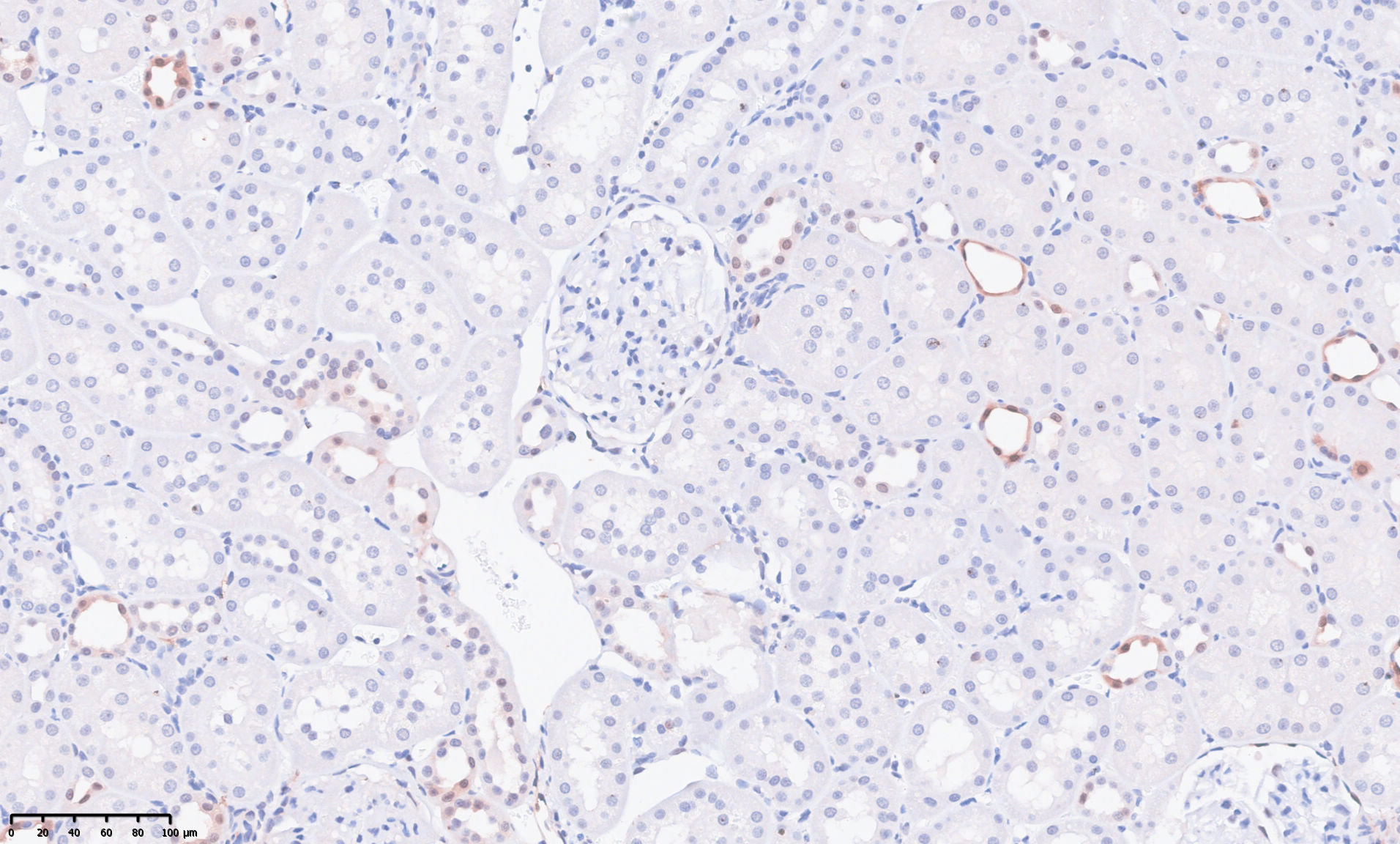

Supplement: Supplementary file 3 [file DataSheet3.zip › IHC staining picture/p-ERK12/syngenic control.jpg]

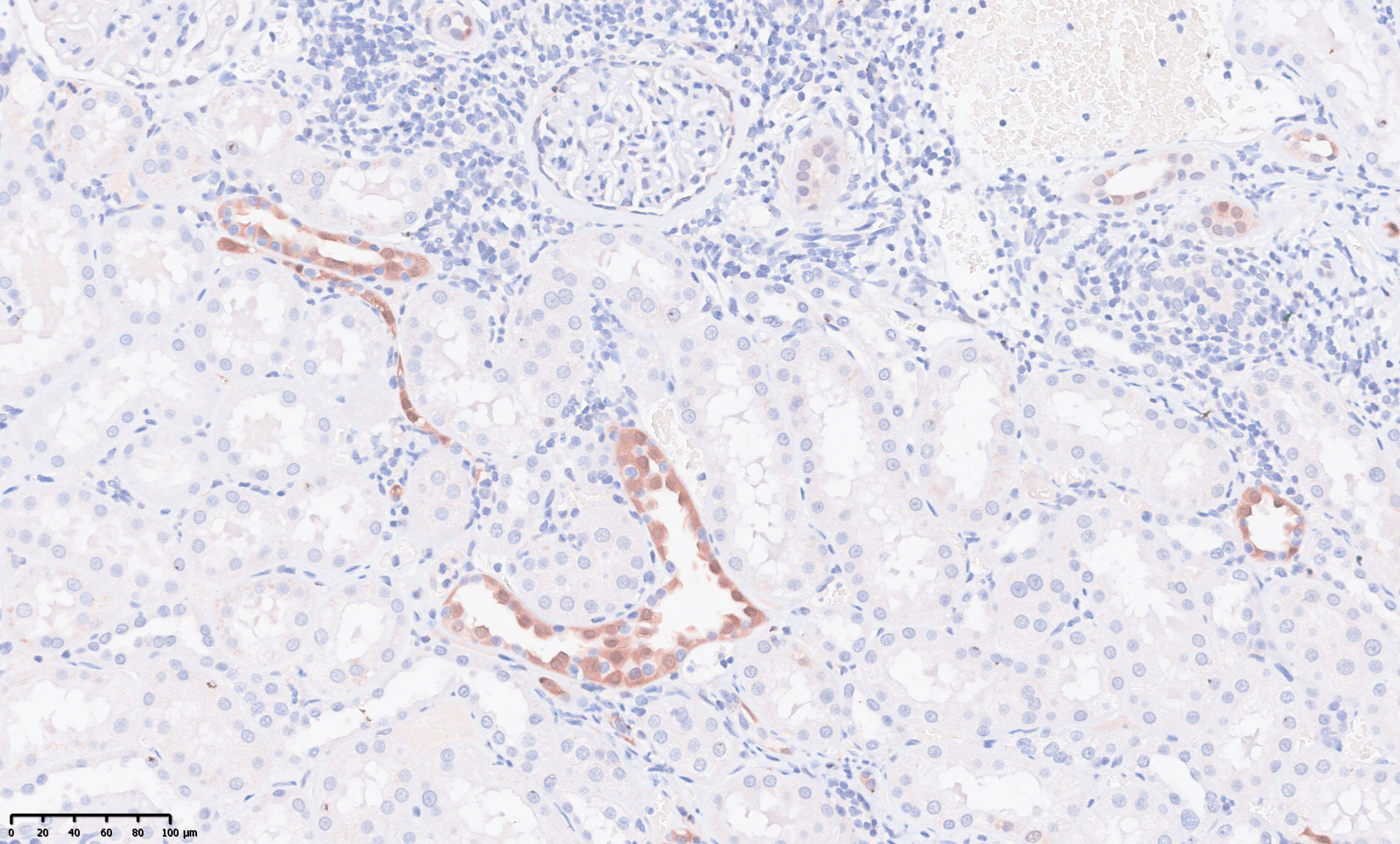

Supplement: Supplementary file 3 [file DataSheet3.zip › IHC staining picture/p-ERK12/TB001 group.jpg]

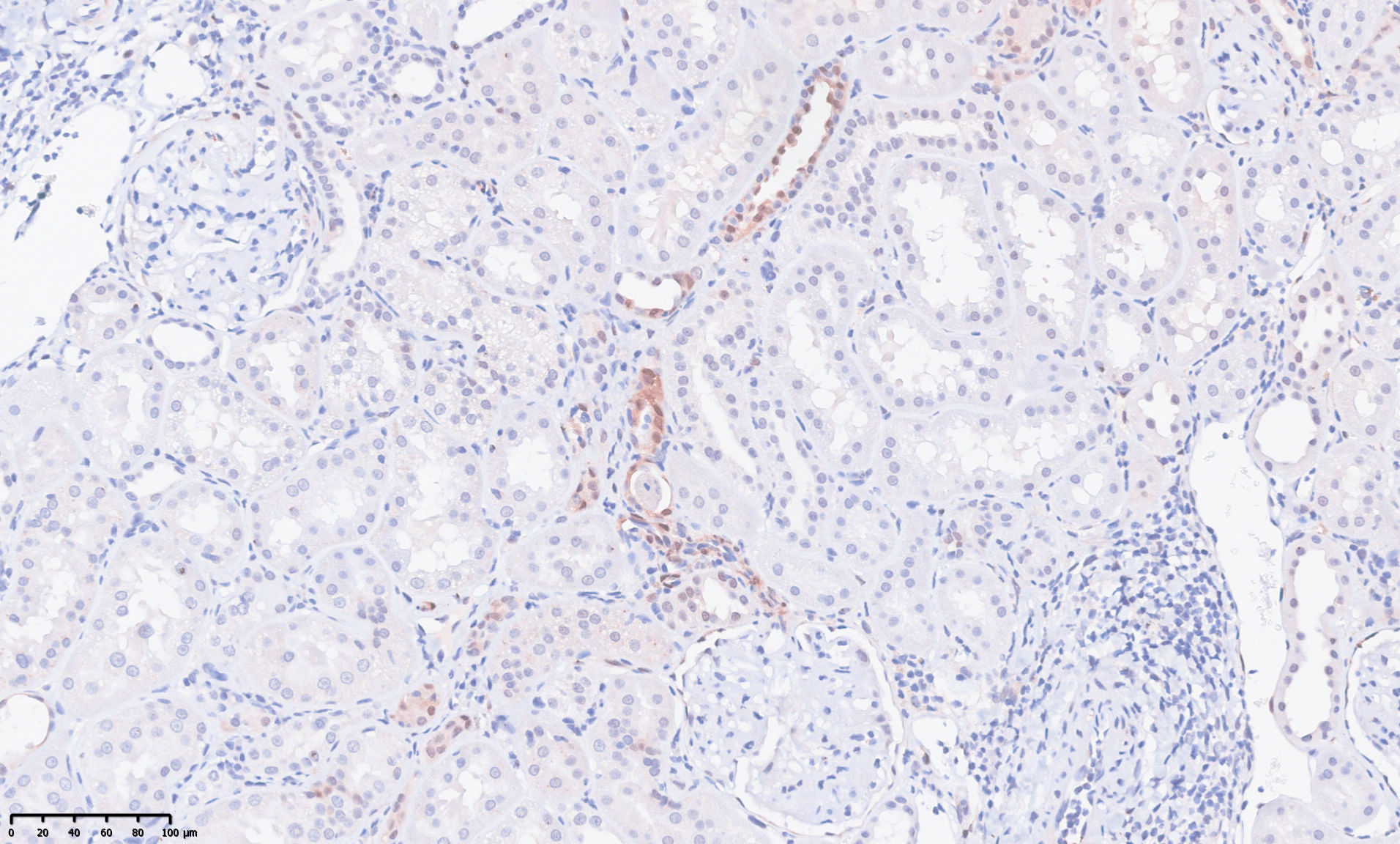

Supplement: Supplementary file 3 [file DataSheet3.zip › IHC staining picture/p-ERK12/Vehicle group.jpg]

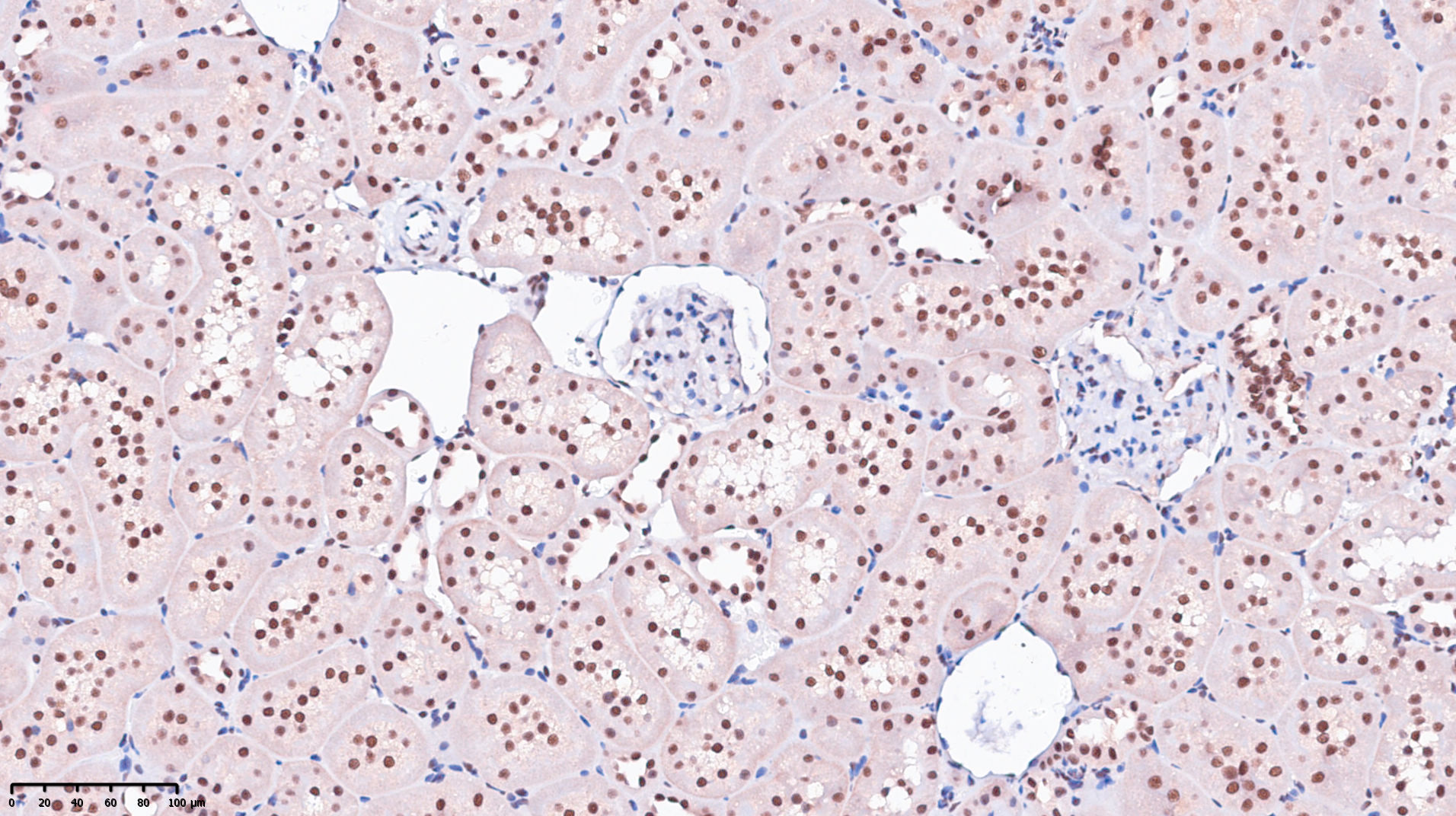

Supplement: Supplementary file 3 [file DataSheet3.zip › IHC staining picture/p-SMAD23/syngenic control.jpg]

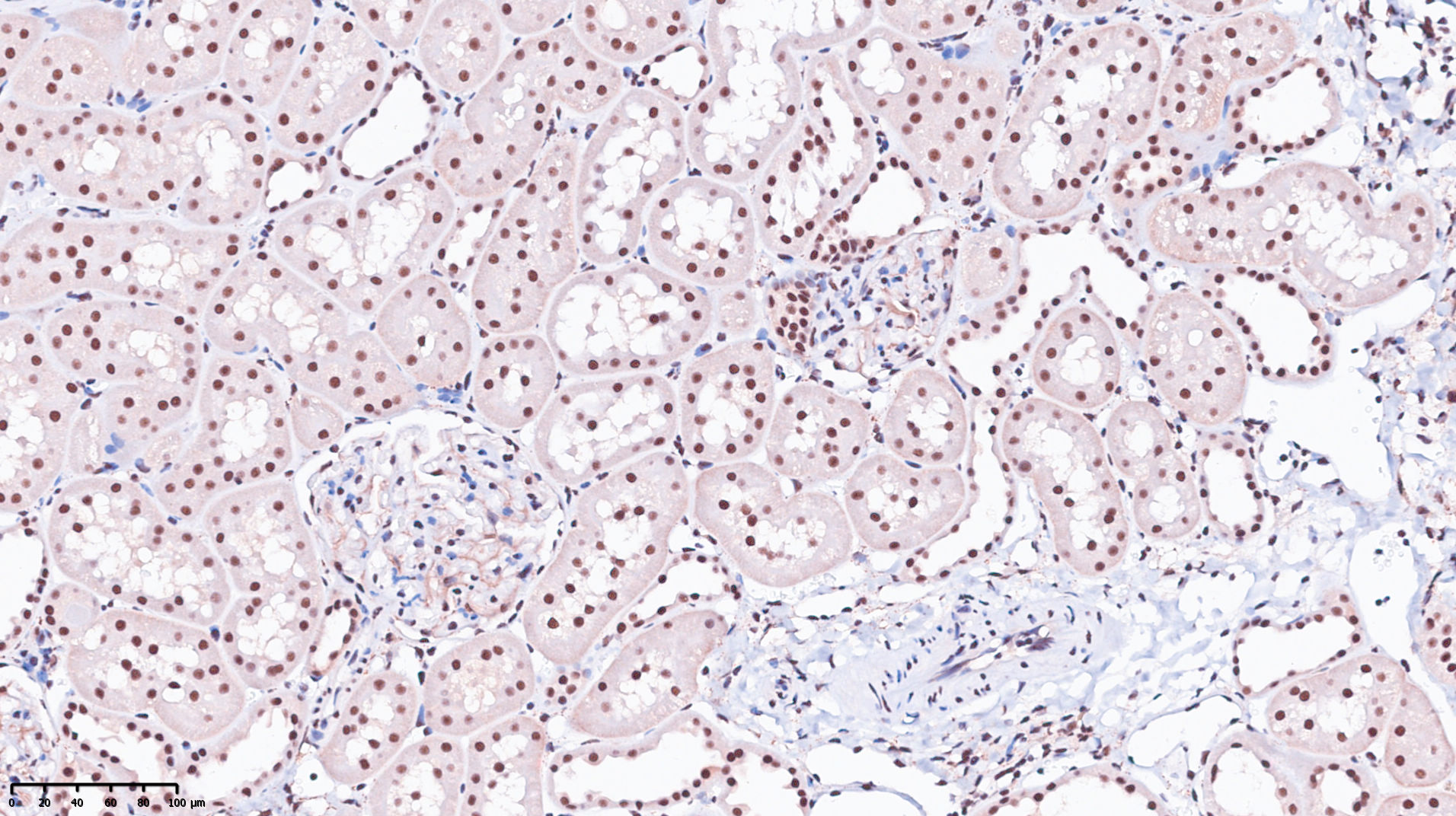

Supplement: Supplementary file 3 [file DataSheet3.zip › IHC staining picture/p-SMAD23/TB001 group.jpg]

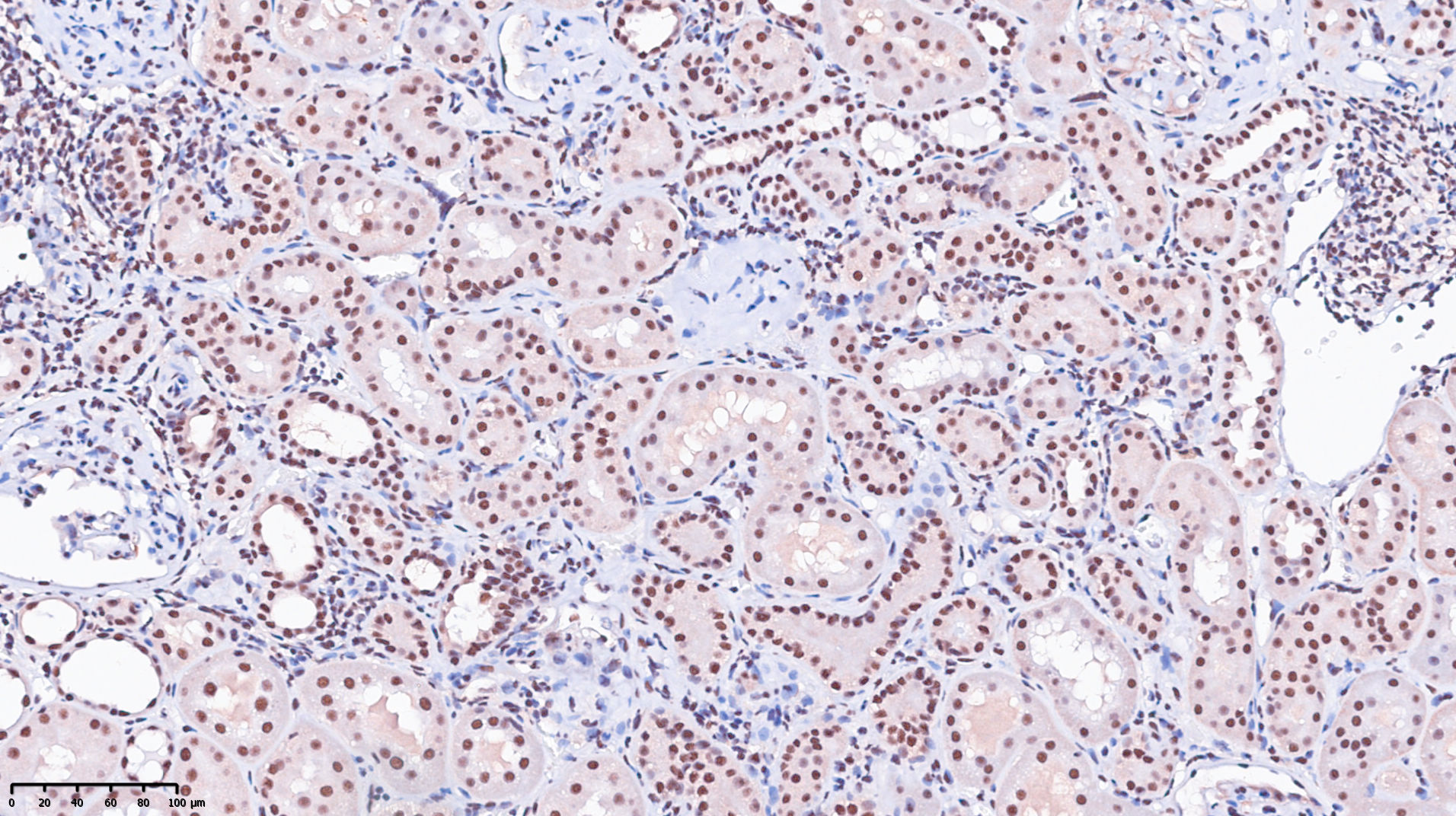

Supplement: Supplementary file 3 [file DataSheet3.zip › IHC staining picture/p-SMAD23/Vehicle group.jpg]

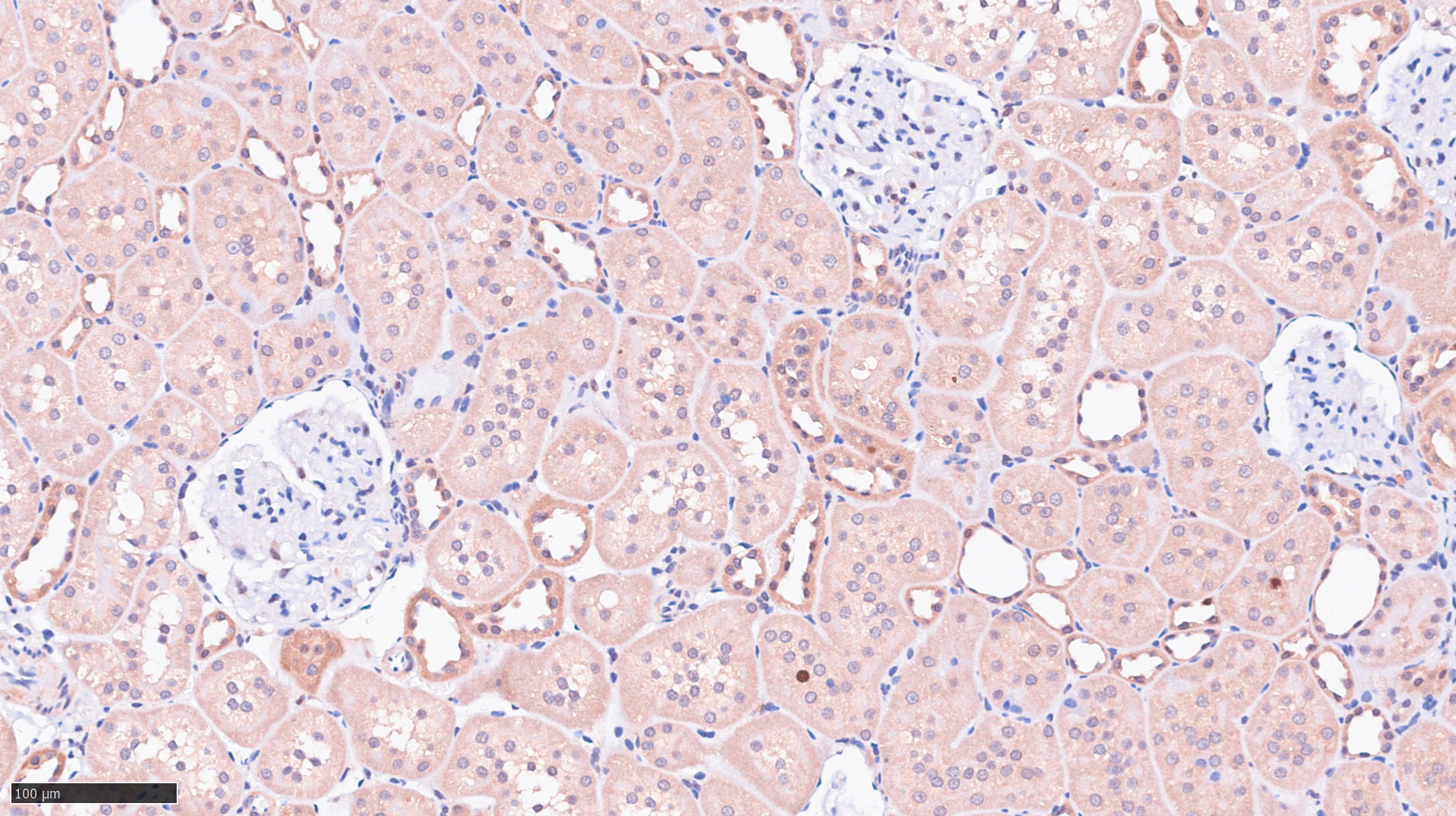

Supplement: Supplementary file 3 [file DataSheet3.zip › IHC staining picture/PKC-β/syngenic control.jpg]

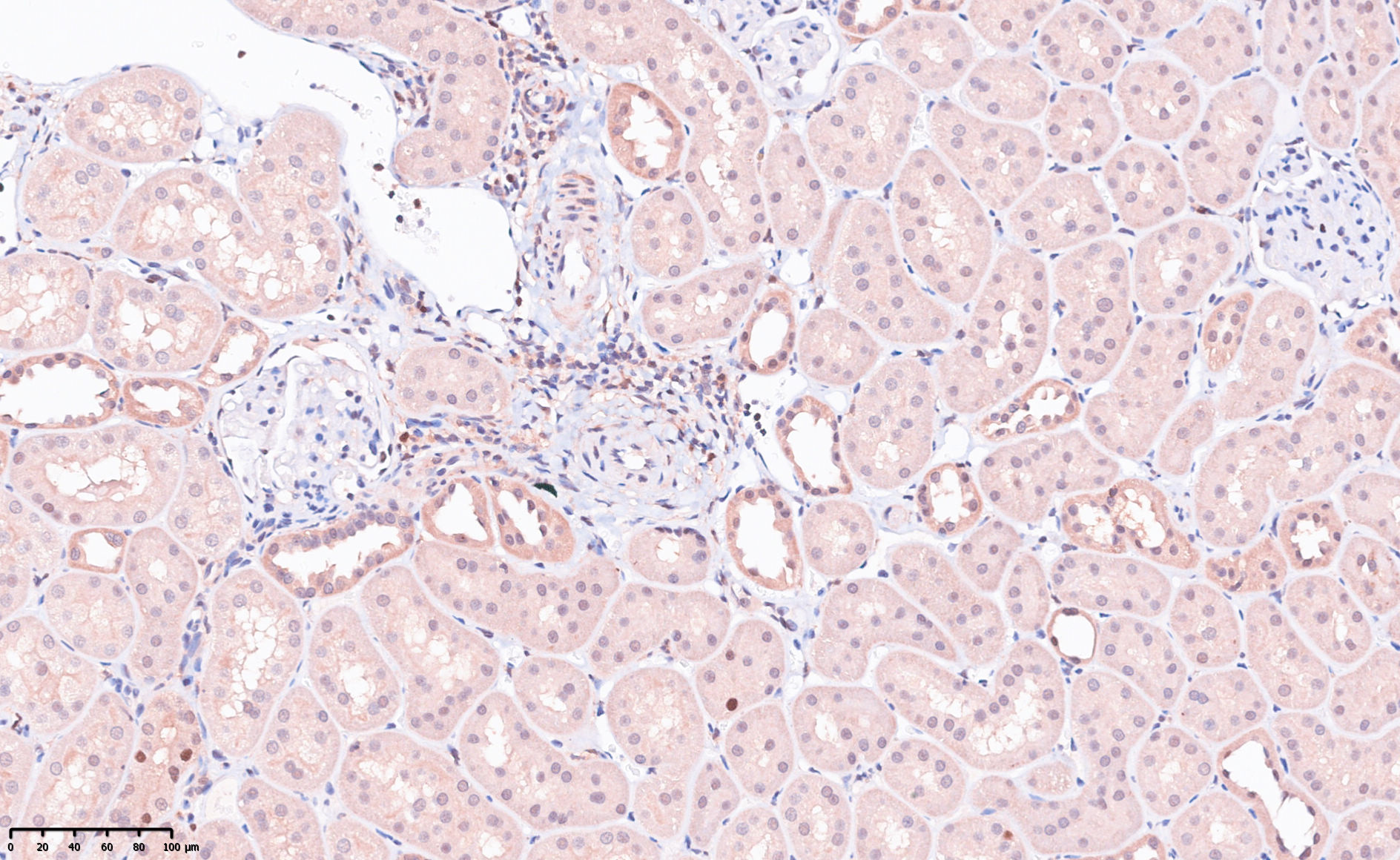

Supplement: Supplementary file 3 [file DataSheet3.zip › IHC staining picture/PKC-β/TB001 group.jpg]

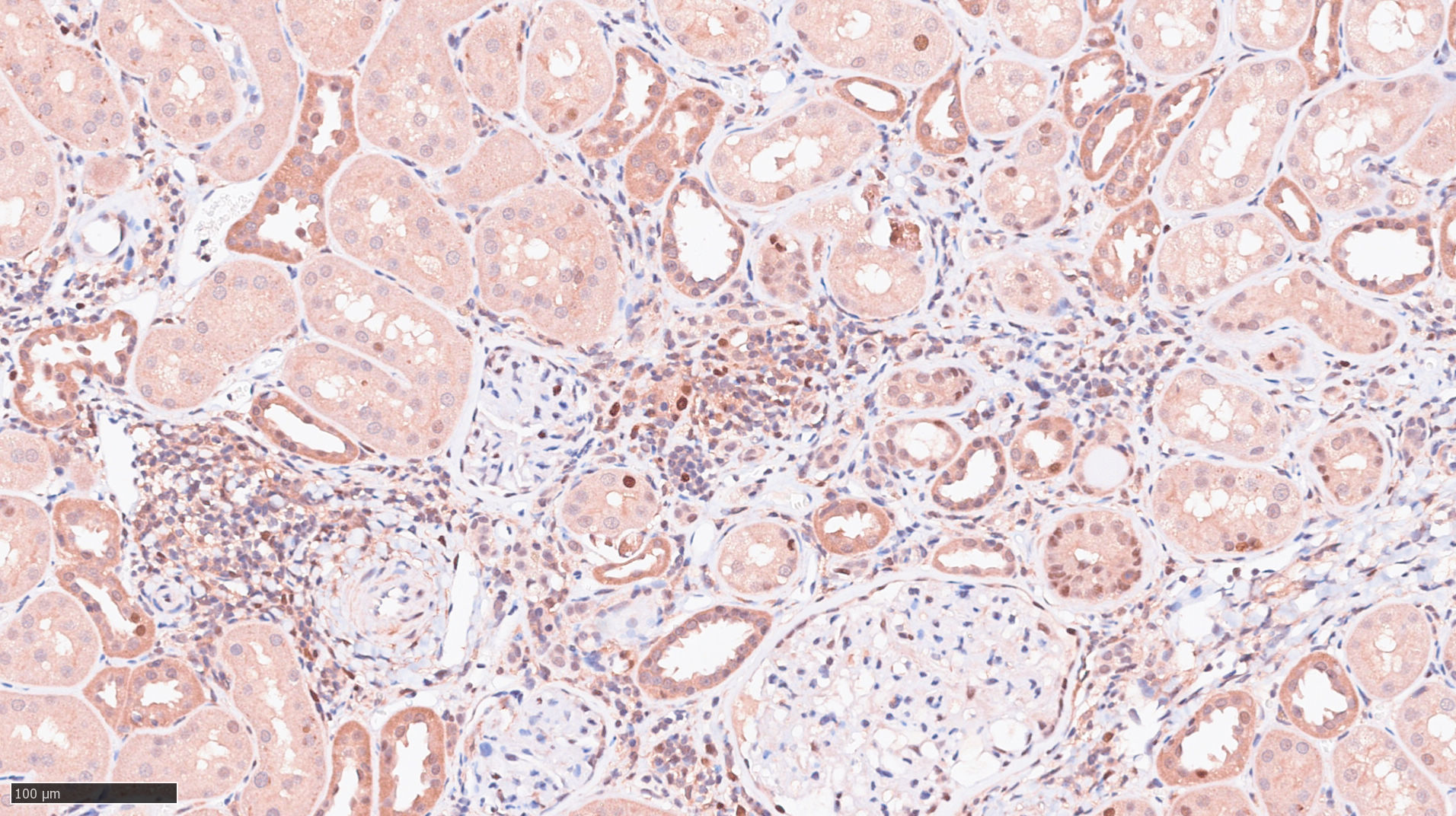

Supplement: Supplementary file 3 [file DataSheet3.zip › IHC staining picture/PKC-β/Vehicle group.jpg]

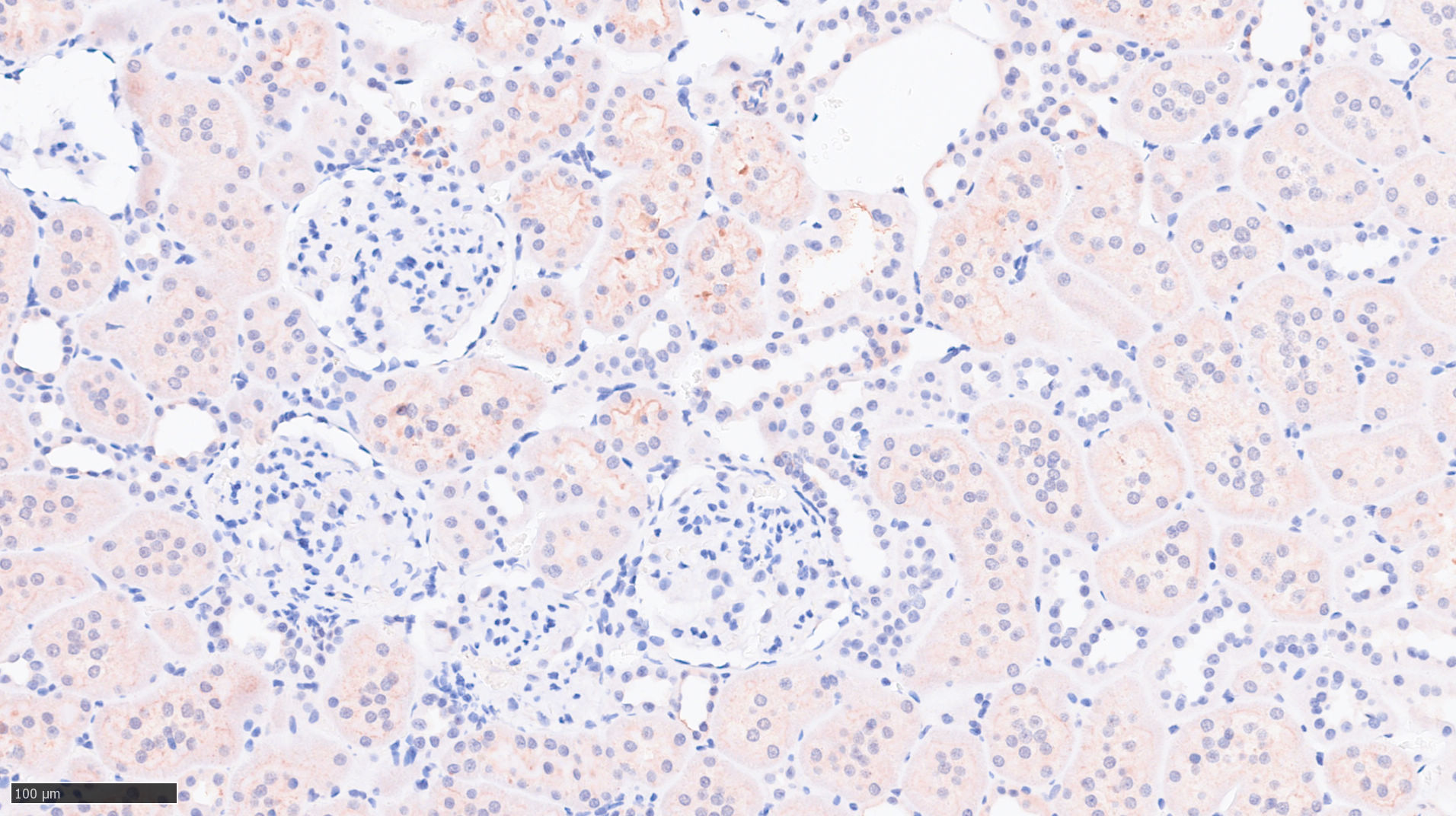

Supplement: Supplementary file 3 [file DataSheet3.zip › IHC staining picture/TGF-β1/syngenic control.jpg]

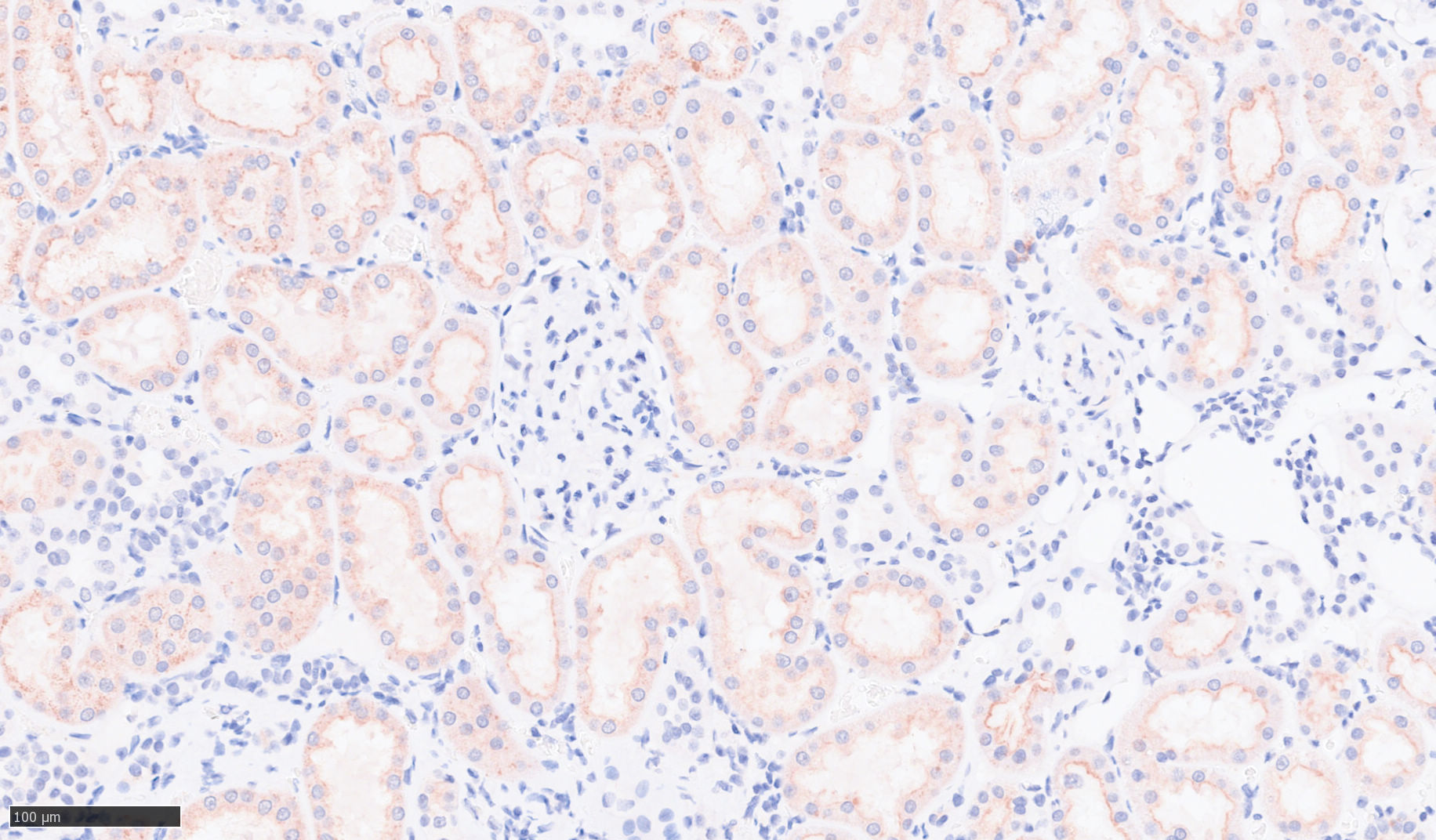

Supplement: Supplementary file 3 [file DataSheet3.zip › IHC staining picture/TGF-β1/TB001 group.jpg]

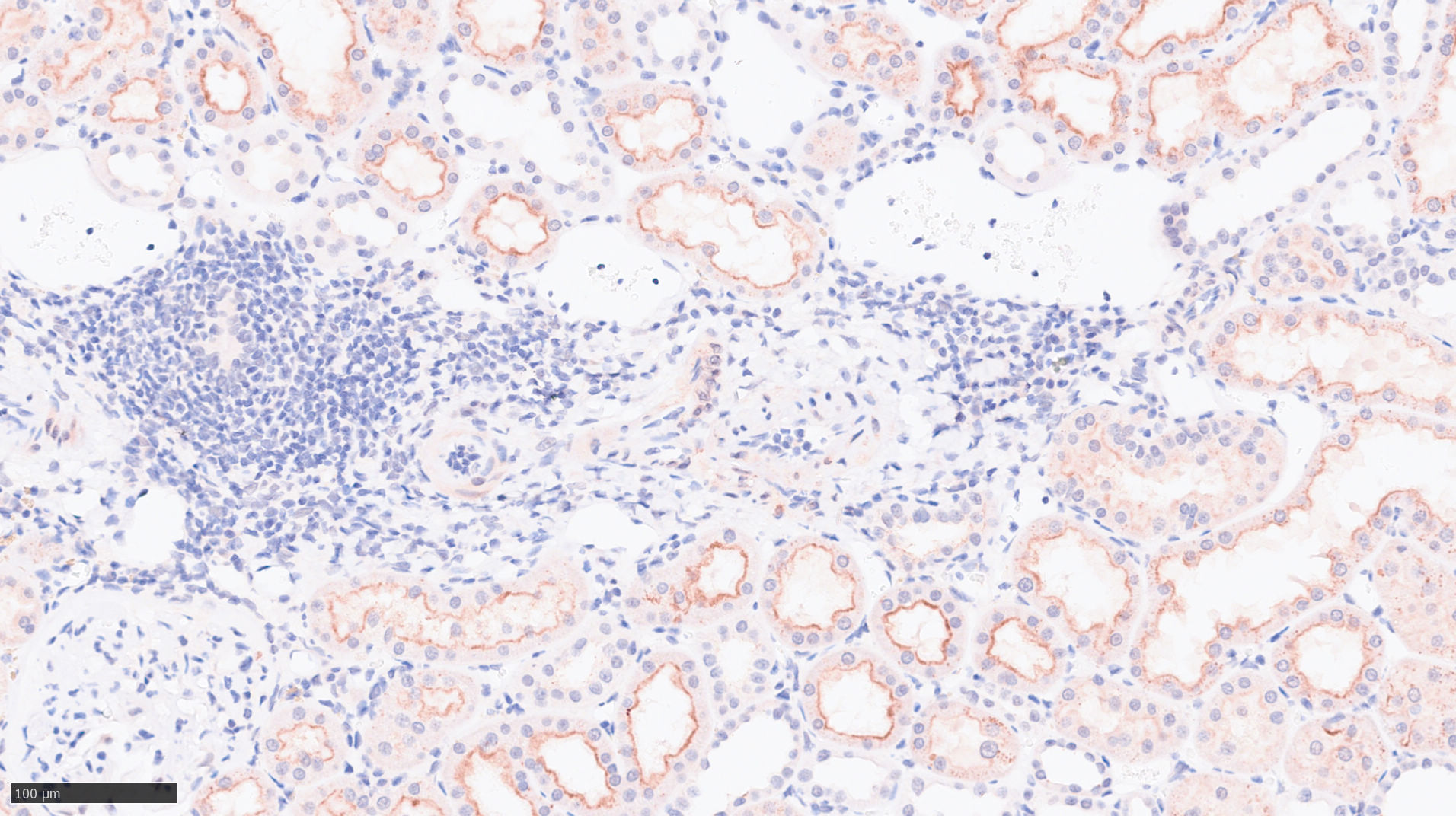

Supplement: Supplementary file 3 [file DataSheet3.zip › IHC staining picture/TGF-β1/Vehicle group.jpg]

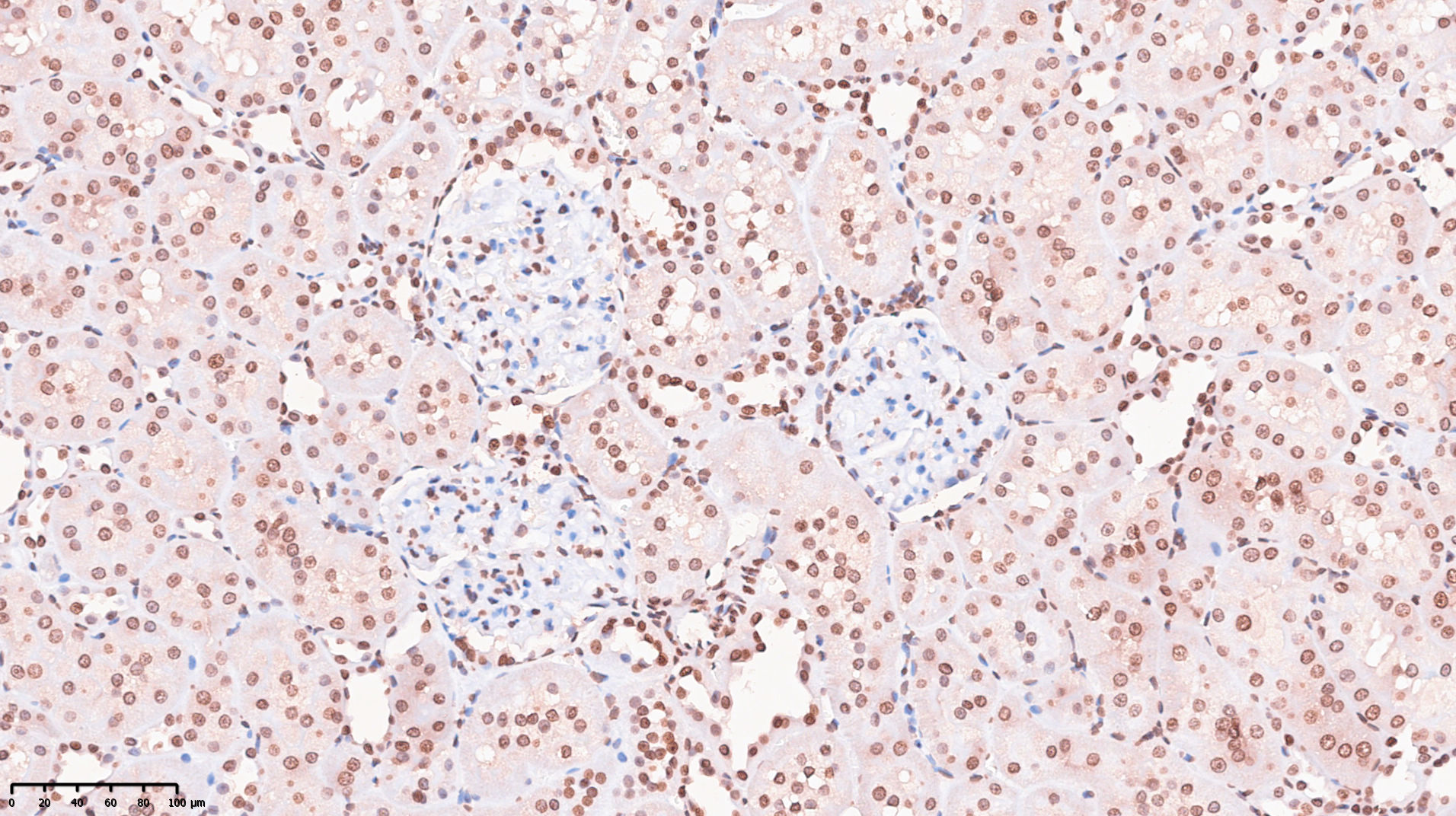

Supplement: Supplementary file 3 [file DataSheet3.zip › IHC staining picture/Twist/syngenic control.jpg]

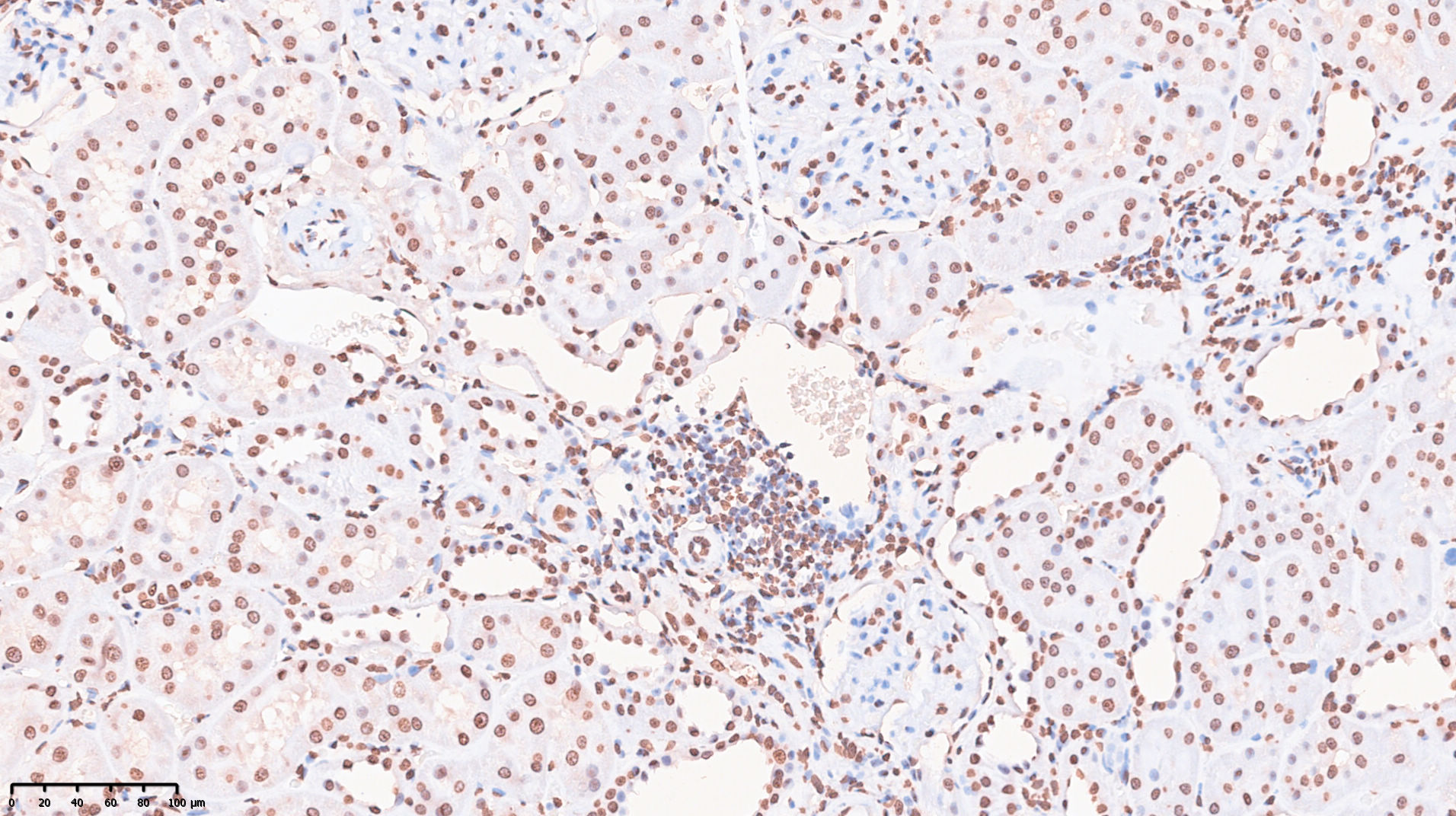

Supplement: Supplementary file 3 [file DataSheet3.zip › IHC staining picture/Twist/TB001 group.jpg]

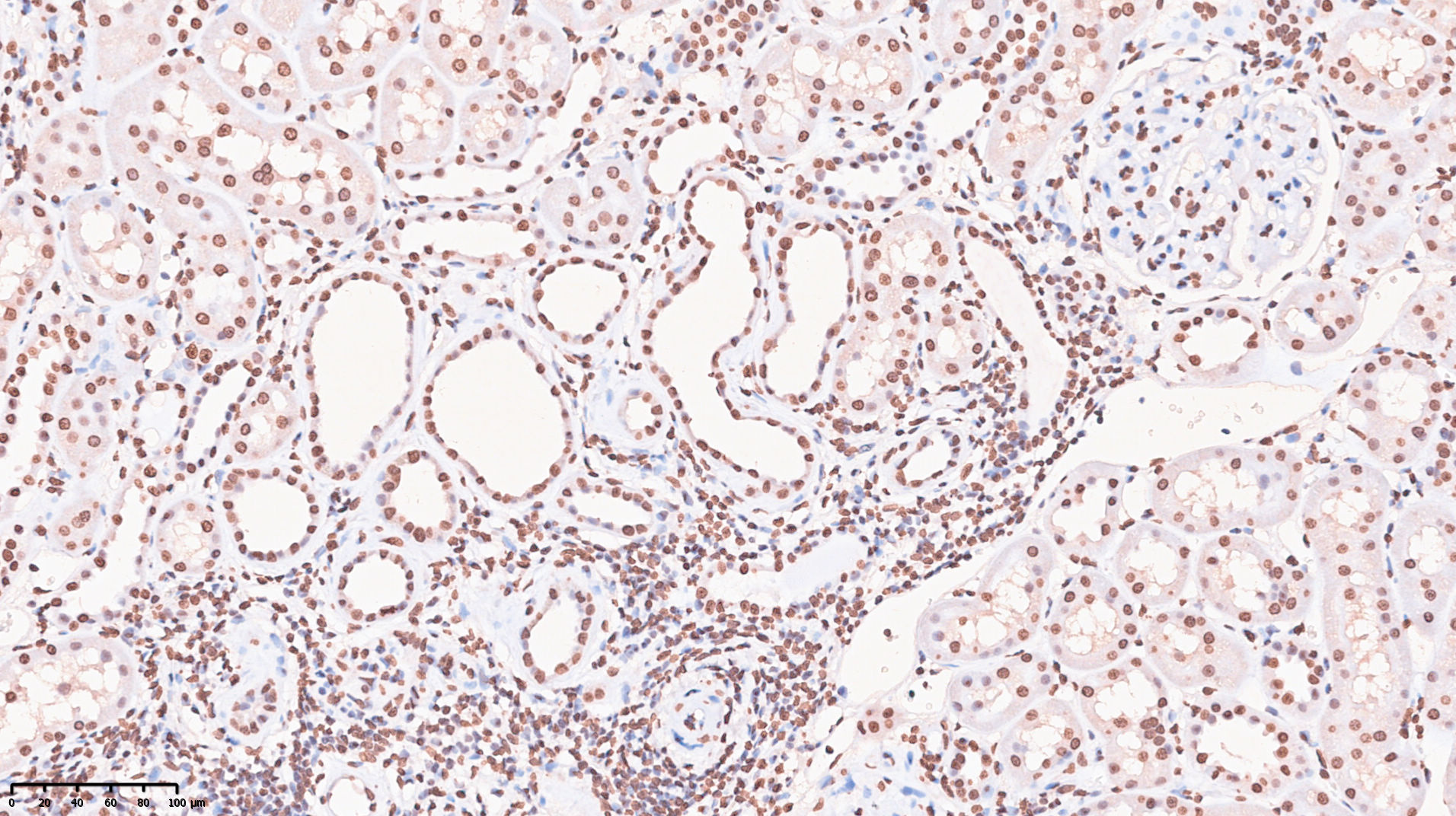

Supplement: Supplementary file 3 [file DataSheet3.zip › IHC staining picture/Twist/Vehicle group.jpg]

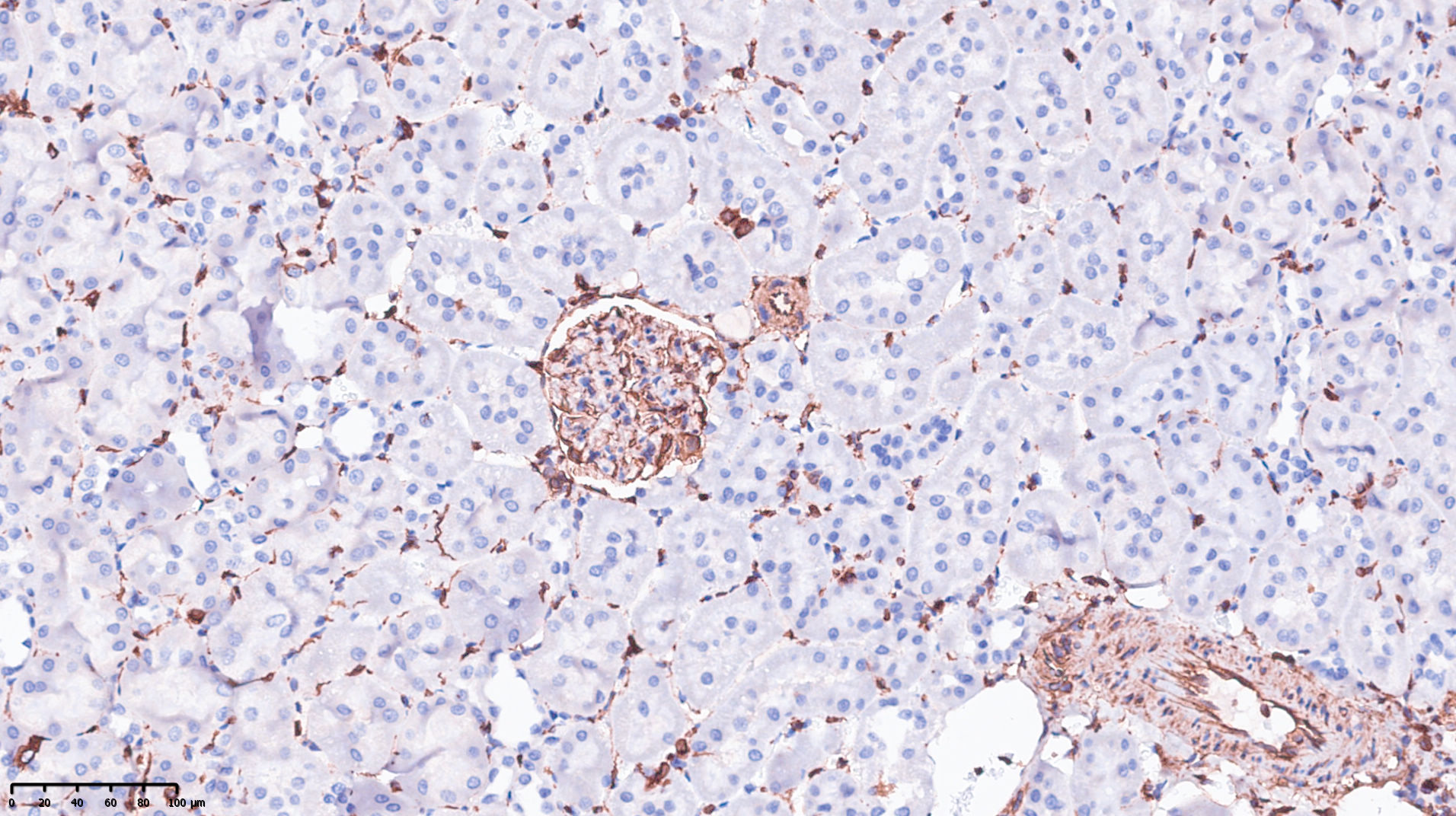

Supplement: Supplementary file 3 [file DataSheet3.zip › IHC staining picture/vimentin/syngenic control.jpg]

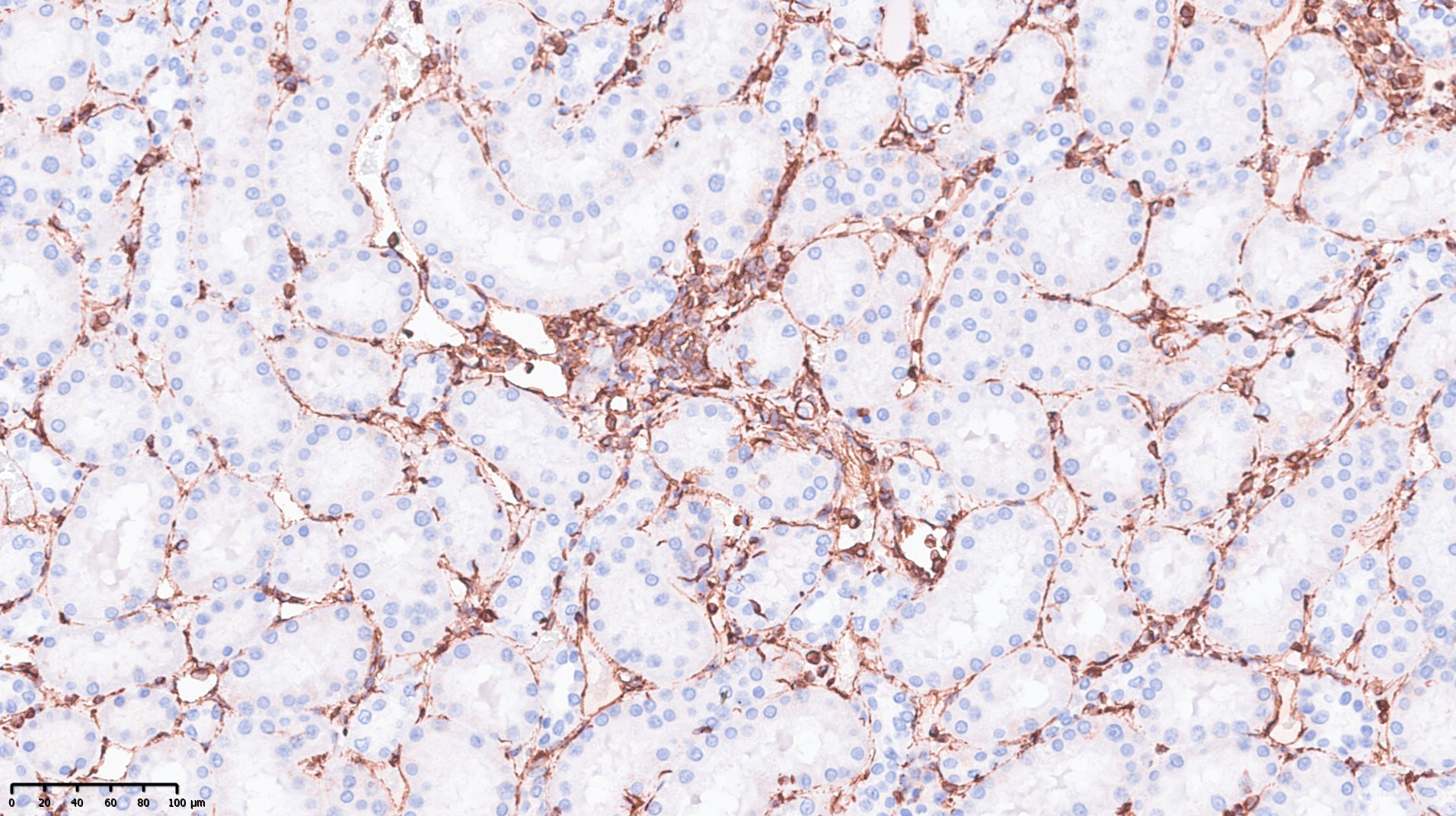

Supplement: Supplementary file 3 [file DataSheet3.zip › IHC staining picture/vimentin/TB001 group.jpg]

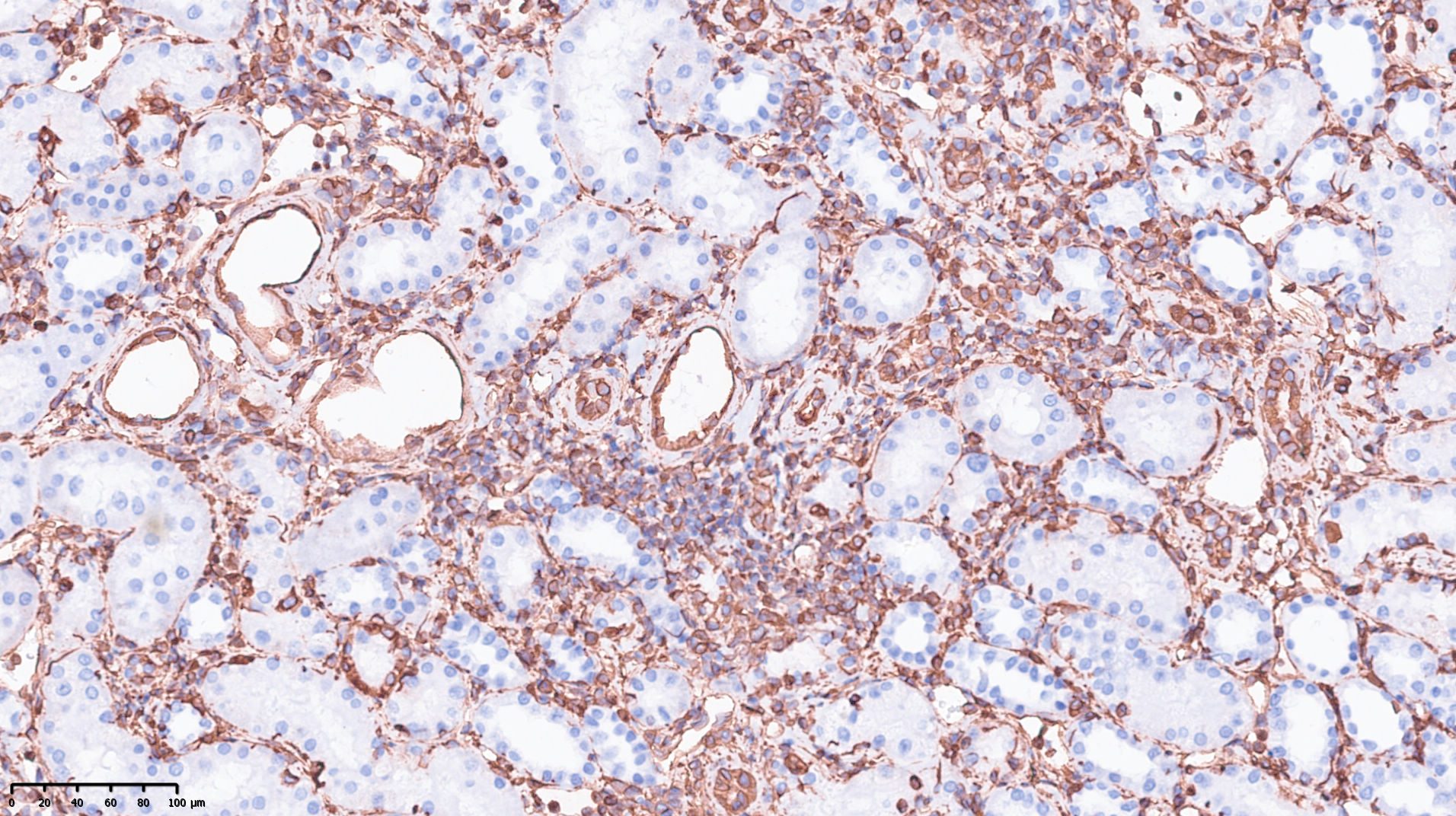

Supplement: Supplementary file 3 [file DataSheet3.zip › IHC staining picture/vimentin/Vechicle group.jpg]

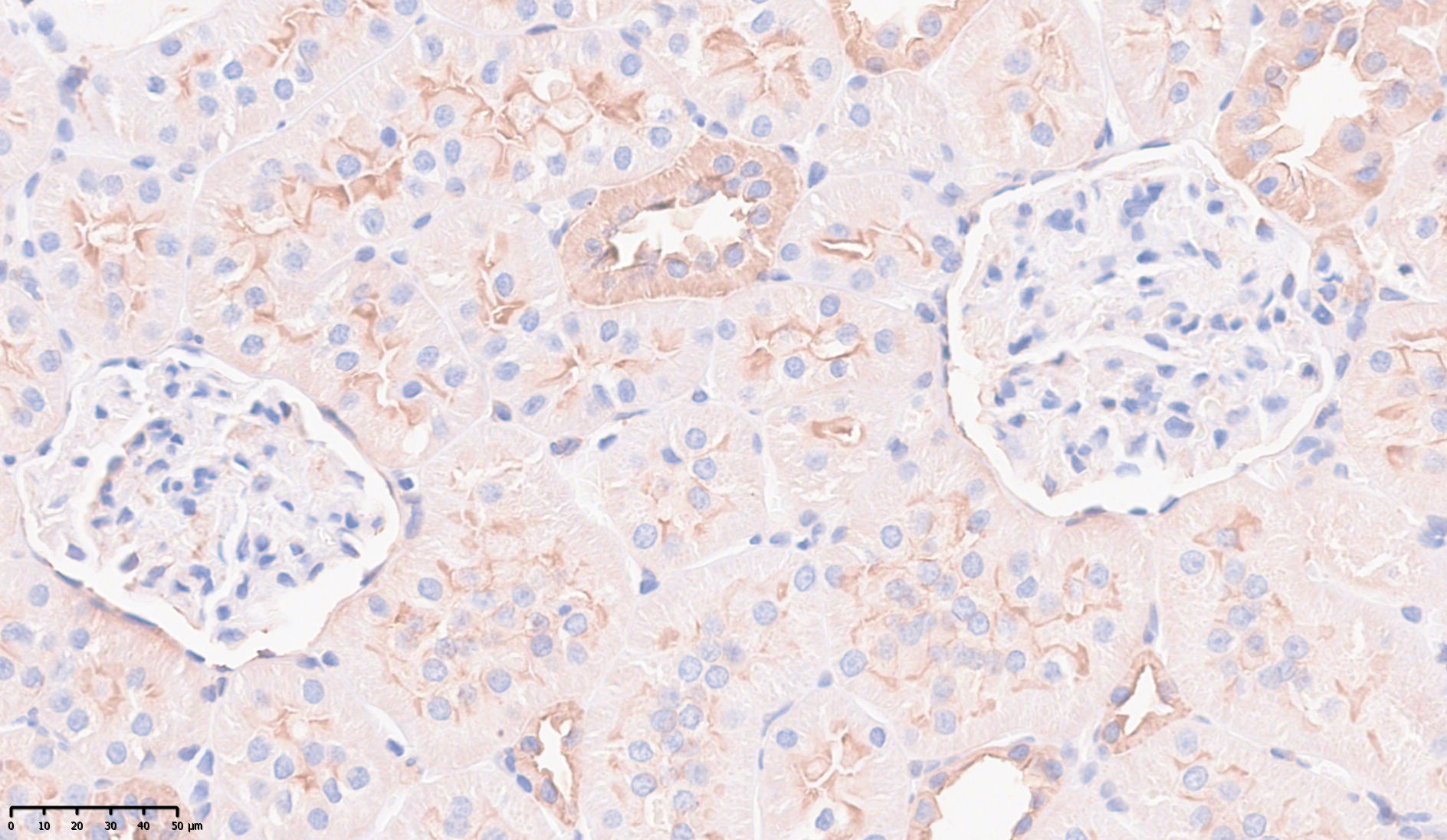

Supplement: Supplementary file 3 [file DataSheet3.zip › IHC staining picture/α-SMA/syngenic control.jpg]

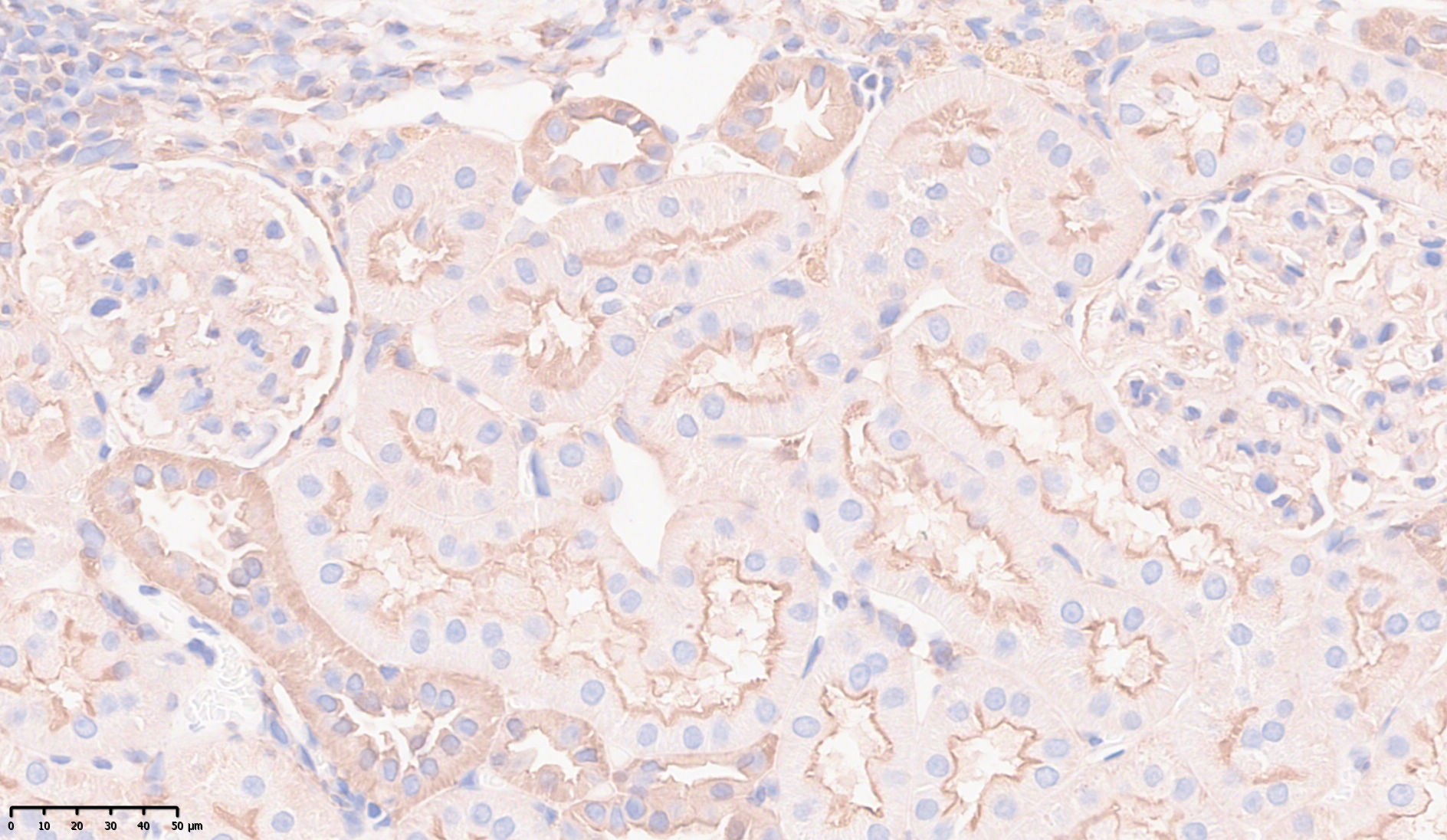

Supplement: Supplementary file 3 [file DataSheet3.zip › IHC staining picture/α-SMA/TB001 group.jpg]

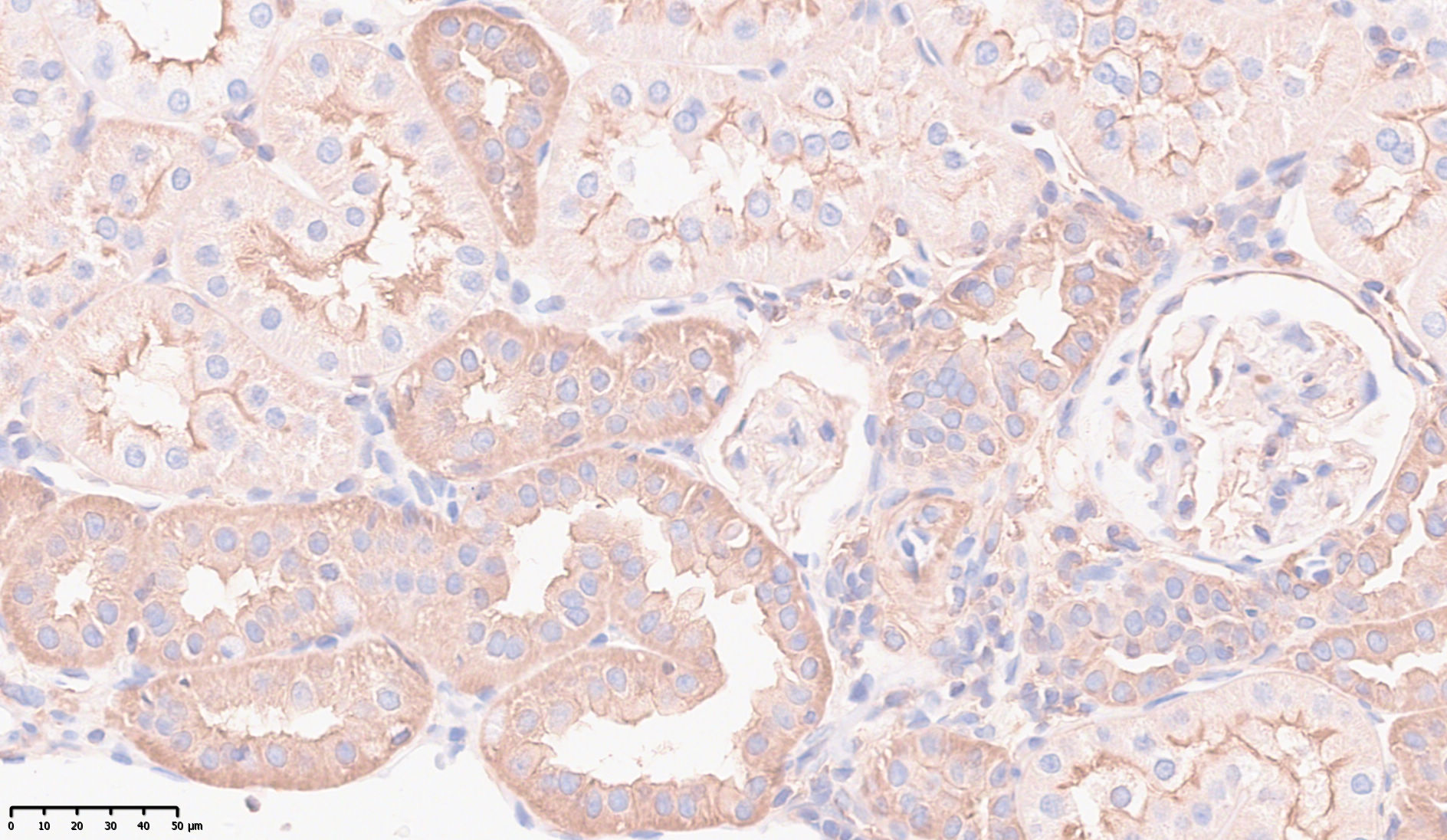

Supplement: Supplementary file 3 [file DataSheet3.zip › IHC staining picture/α-SMA/Vehicle group.jpg]

# PCA 3D

- Chronic
- Syngenic
- ▲ TB001

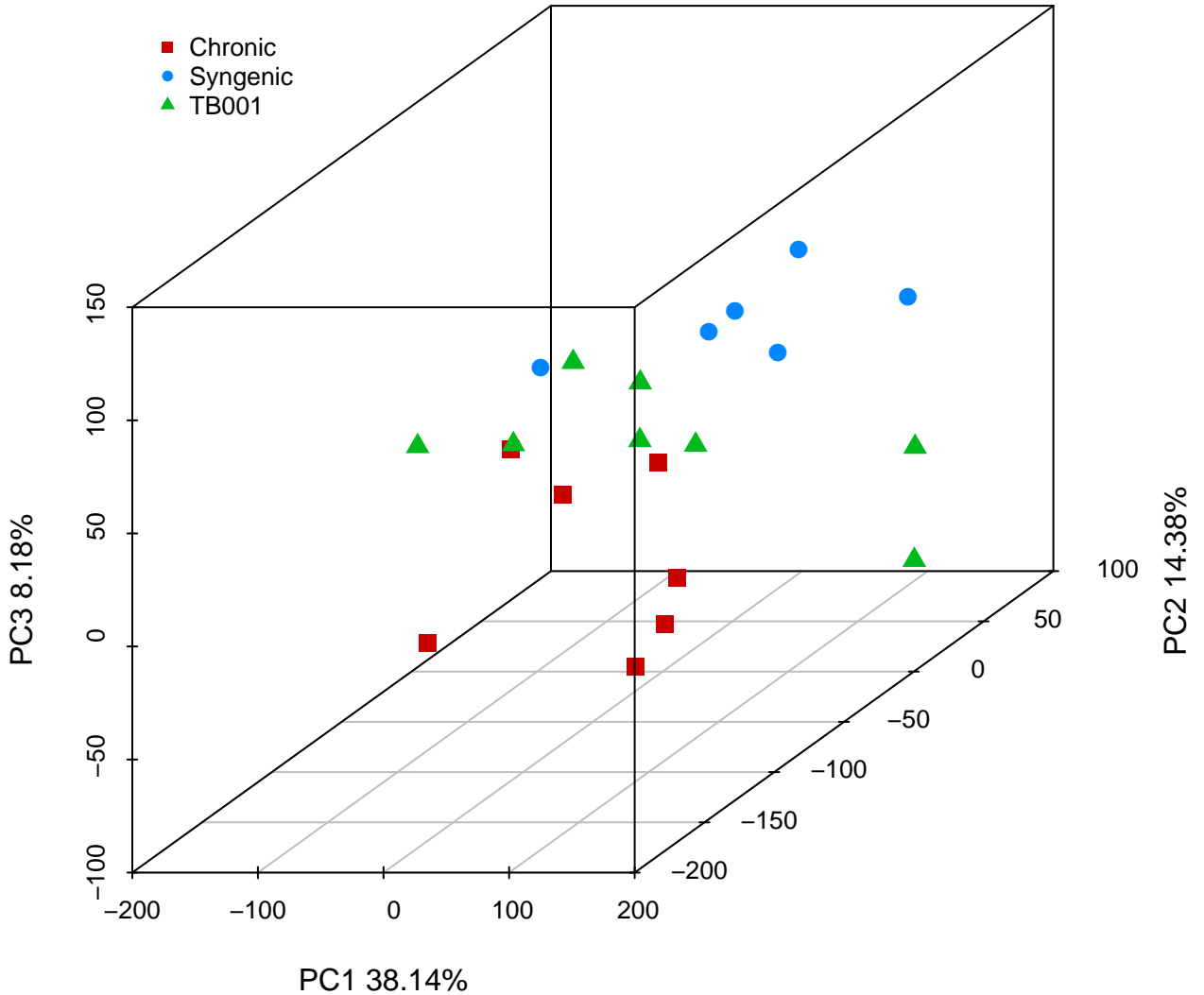

# PCA – PC1 vs PC2

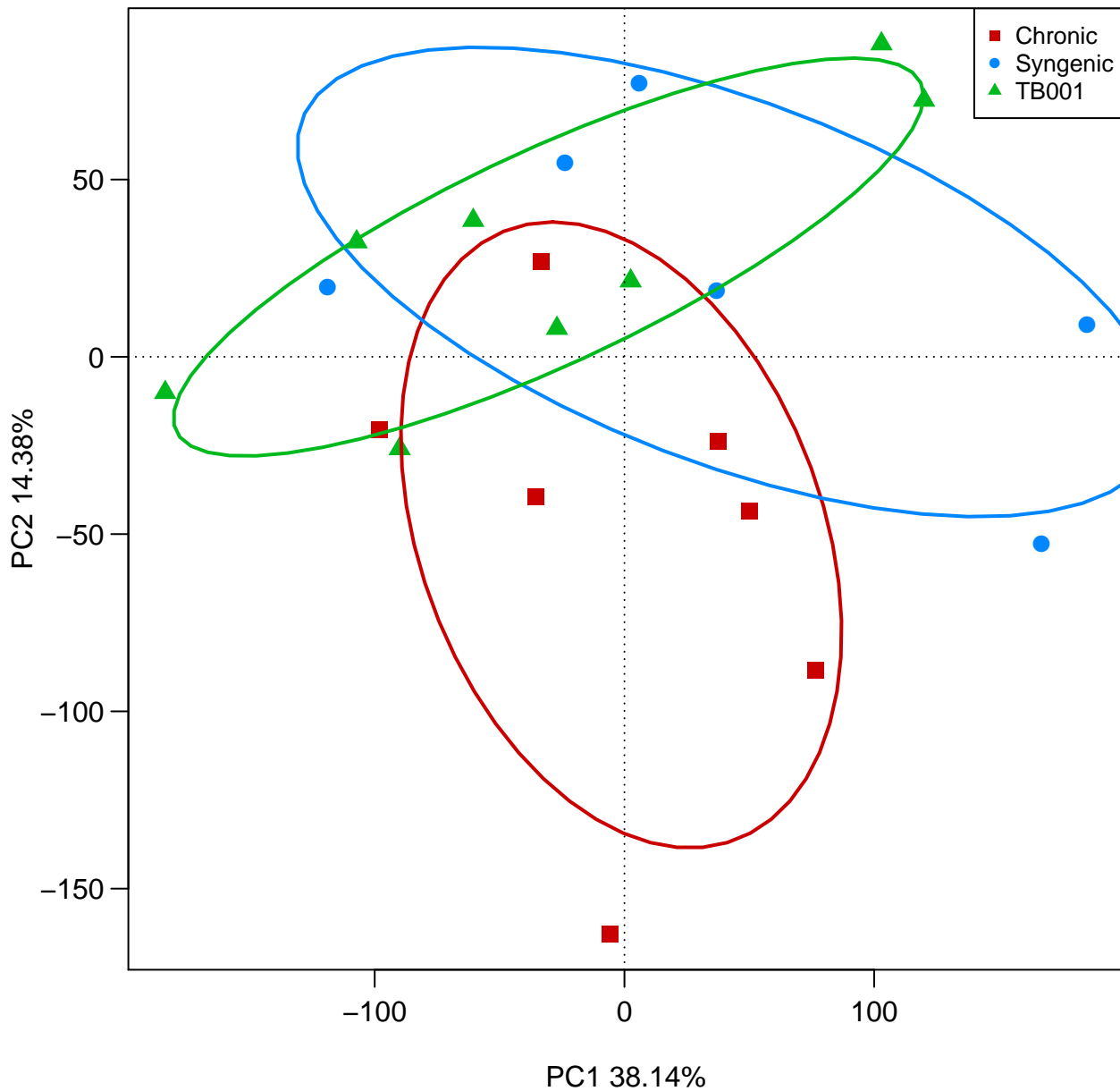

PCA – PC1 vs PC3

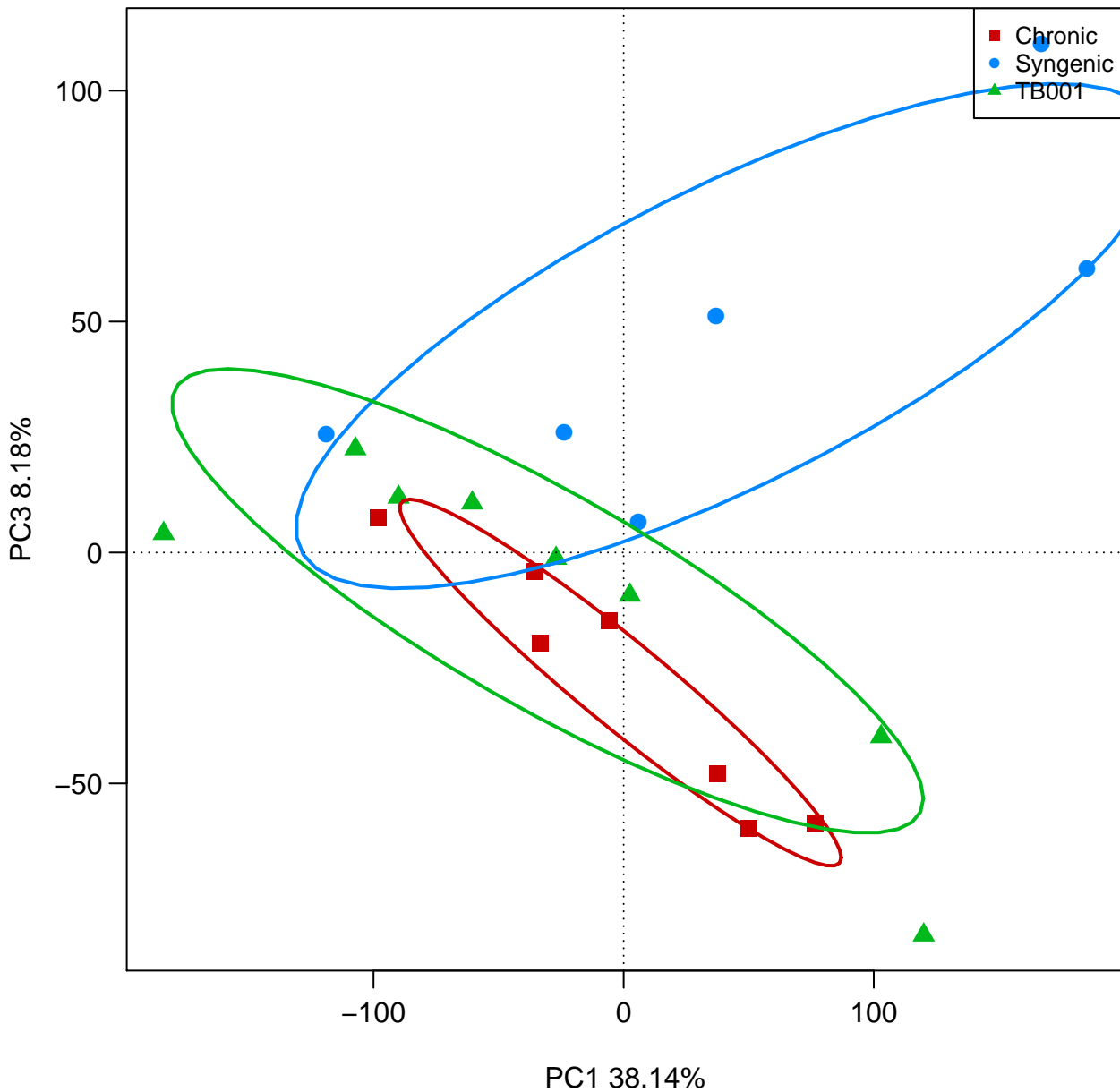

# PCA – PC2 vs PC3

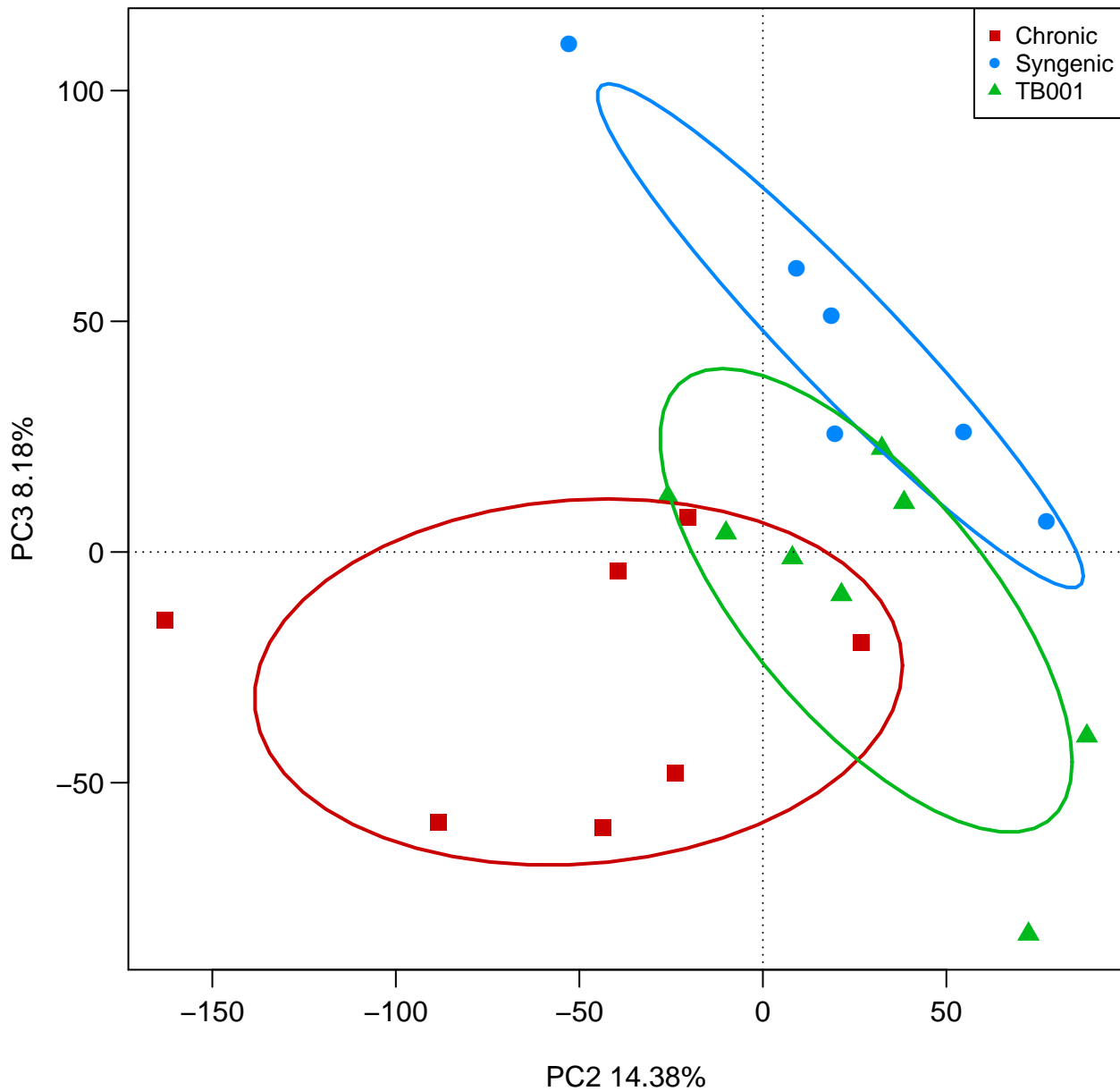

Supplement: Supplementary file 4 [file DataSheet4.pdf]
