# Supplementary material for: GLP-1 and glucagon receptor dual agonism ameliorates kidney allograft fibrosis by improving lipid metabolism
Source: Front Immunol. 2025 Mar 31;16:1551136. doi: 10.3389/fimmu.2025.1551136 (PMC11994718; doi:10.3389/fimmu.2025.1551136)
Supplement: Supplementary file 5 [file Table1.docx]

**Table.1 Primers details for rat qPCR.**

| Genes | Forward primer（5′-3′） | Reverse primer（5′-3′） |
| --- | --- | --- |
| α-SMA | CATCCGACCTTGCTAACGGA | AATAGCCACGCTCAGTCAGG |
| FN | CGGTGGCTGTCAGTCAAAG | AAACCTCGGCTTCCTCCATAA |
| Vimentin | TCAGACAGGATGTTGACAAT | GACATGCTGTTCCTGAATCT |
| E-cad | AGGTCGGTGCCCGTATTG | TGGTCTTGGGGTCTGTGATG |
| PKC-α | GCCGCAGTGTCGTTTATGAAAGTA | GCTCCATGTGTGCCCATTCAATTAG |
| PKC-β | GGCGTTTGGAGTCCTGCTGTA | CCACGTTGTGCTCCATGATTG |
| Snail1 | GGTTCCTGCTTGGCTCTCTT | AGCTGTGTCCAGAGGCTACAC |
| Twist | AGCTGAGCAAGATTCAGACC | AGCTTGCCATCTTGGAGTC |
| β-actin | GCCTTCCTTCCTGGGTATGG | AATAGCCACGCTCAGTCAGG |
