# Supplementary material for: GLP-1 and glucagon receptor dual agonism ameliorates kidney allograft fibrosis by improving lipid metabolism
Source: Front Immunol. 2025 Mar 31;16:1551136. doi: 10.3389/fimmu.2025.1551136 (PMC11994718; doi:10.3389/fimmu.2025.1551136)
Supplement: Supplementary file 6 [file Table2.docx]

**Table.2 Information of different groups in the in vitro experiment.**

|  | PMA group | TB001  group | GO6983  group | PD98059  group |
| --- | --- | --- | --- | --- |
| TGF-β1 | 10ng/mL | 10ng/mL | 10ng/mL | 10ng/mL |
| PMA | 11.7nM | 11.7nM | 11.7nM | 11.7nM |
| TB001 |  | 10μM |  |  |
| GO6983 |  |  | 7nM |  |
| PD98059 |  |  |  | 10μM |
